# Genomic characteristics and epidemic trends of NADC30-like PRRSV in China

**DOI:** 10.1186/s40813-025-00444-7

**Published:** 2025-05-28

**Authors:** Siyu Zhang, Hu Xu, Zhenyang Guo, Lirun Xiang, Chao Li, Bangjun Gong, Jinhao Li, Zixuan Feng, Haonan Kang, Qian Wang, Guohui Zhou, Chaoliang Leng, Kuan Zhao, Yan-Dong Tang, Huairan Liu, Tong-Qing An, Xuehui Cai, Jinmei Peng, Zhi-Jun Tian, Hongliang Zhang

**Affiliations:** 1 State Key Laboratory for Animal Disease Control and Prevention, Harbin Veterinary Research Institute, Chinese Academy of Agricultural Sciences, No. 678 Haping Road, Xiangfang District, Harbin, 150001 China; 2 Henan Provincial Engineering and Technology Center of Animal Disease Diagnosis and Integrated Control, Nanyang Normal University, Nanyang, 473061 China; 3 College of Veterinary Medicine, Hebei Agricultural University, Baoding, 071000 China

**Keywords:** NADC30-like PRRSV, Recombination patterns, Mainstream branch, Genomic characteristics, Epidemic trend

## Abstract

**Background:**

NADC30-like PRRSV was first identified in China in 2012 and had become the predominant circulating strain since 2016. Currently, the recombination patterns of NADC30-like PRRSV in China exhibit a high degree of complexity, characterized by low whole-genome sequence homology. The genomic features and epidemiological trends of these strains remain to be elucidated.

**Results:**

To evaluate the prevalence of NADC30-like PRRSV in China, this study acquired 30 whole-genome sequences of NADC30-like strains via Next-Generation Sequencing (NGS). These sequences were subsequently integrated with 224 whole-genome sequences from China available in the GenBank database. A comprehensive analysis of the genomic characteristics of contemporary NADC30-like PRRSV strains in China was conducted. Recombinant analysis indicated a yearly increase in the number of NADC30-like strains exhibiting recombination signals, whereas nonrecombinant NADC30-like strains have become nearly extinct. Among the recombination events, those involving L1C and L8E as parental strains are most prevalent. Based on the results of recombination and phylogenetic analyses, this study classified 120 Chinese NADC30-like strains with similar recombination characteristics into groups NADC30-R1 to R12. The intra-group genetic distances of the NADC30-R1 to R12 groups approximately 5.73% (SD ± 1.68), while the inter-group genetic distances between different groups are usually stably greater than 10%. The amino acid alignment of Nsp2 demonstrated that all NADC30-R1 to R12 strains exhibit a discontinuous deletion of 131 amino acids. These classifications do not exhibit consistent pathogenic characteristics within groups, with most NADC30-like PRRSVs showing moderate virulence. Geographical distribution analysis indicated that NADC30 whole-genome sequences in China originated from 19 provinces. Notably, the NADC30-R1 and NADC30-R2 strains are the most widely distributed and abundant, suggesting that these variants have established localized epidemics in specific regions.

**Conclusion:**

In summary, the vast majority of NADC30-like strains in our country have undergone recombination, L1C + L8E is the most common recombination mode. The NADC30-like strains in China can be classified into 12 different recombination patterns, NADC30-R1 and NADC30-R2 strains are already showing pandemic trends. These findings provide a critical foundation for future NADC30-like PRRSV prevention and control strategies.

**Supplementary Information:**

The online version contains supplementary material available at 10.1186/s40813-025-00444-7.

## Introduction

Porcine reproductive and respiratory syndrome (PRRS) is among the most significant epidemic diseases impacting the global swine industry, initially identified in the United States in 1987 [1, 2]. The causative agent, porcine reproductive and respiratory syndrome virus (PRRSV), is an enveloped, single-stranded positive-sense RNA virus. Its genome, approximately 15 kb in length, comprises a 5′ untranslated region (UTR), at least eleven open reading frames (ORFs), a 3′ UTR, and a polyadenylated tail at the 3′ end [3, 4]. PRRSV is categorized into two distinct species: PRRSV-1 (*Betaarterivirus suid 1*) and PRRSV-2 (*Betaarterivirus suid 2*). The nucleotide sequence homology between these two species is less than 60% [5–7]. In China, since its initial report in 1996, PRRSV-2 has remained dominant.

Among the structural genes of PRRSV, the ORF5 gene plays a crucial role in viral assembly, infectivity, and the induction of neutralizing antibodies [8, 9]. Given the high genetic diversity of ORF5, it has become a widely utilized tool for phylogenetic analysis. Recently, based on the genetic relationships among ORF5 sequences, PRRSV-2 has been subdivided into 11 genetic lineages (L1‒L11) and 21 sublineages (L1A-L1F, L1H-L1J, L5A-L5B, L8A-L8E, and L9A-L9E) [10]. Lineage 1 of PRRSV-2 is the most prevalent group, exhibiting the highest detection rate and broadest genetic diversity globally [11–13]. Based on the findings of our laboratory’s prior research, lineages 1, 3, 5, and 8 strains have been the most common in China from 1996 to 2023 [14–19].

Chinese NADC30-like PRRSV was reportedly introduced from North America [20]. Subsequently, it primarily disseminated from the mid-southern region of China, particularly Henan and Guangdong provinces, as well as the eastern region, notably Fujian and Shandong provinces, to other parts of the country [21]. This strain exhibits new epidemic characteristics, including moderate clinical symptoms, enhanced immunosuppressive capacity, and a diminished antibody response [11–13]. Our previous studies indicate that NADC30-like strains have replaced HP-PRRSV to become the predominant strain in China since 2016 [22]. It is crucial to monitor and provide early warnings regarding any new changes in the population evolution dynamics of NADC30-like PRRSV.

In recent years, there has been a notable increase in reports documenting complex recombination events involving NADC30-like PRRSV [20, 23, 24]. Notably, the ORF5 gene is comprises only 4% of the viral genome [25, 26]. Therefore, whole-genome sequencing (WGS) studies are imperative to provide a comprehensive understanding of the virus, enabling accurate prediction of genetic variations. By systematically analyzing the genetic relationships and evolutionary patterns, recombination profiles, and Nsp2 insertion/deletion patterns of the latest strains and all previously reported genome-wide sequences of NADC30-like PRRSV in China, this study elucidates the comprehensive genomic characteristics of Chinese NADC30-like strains. It also explores their classification and pathogenicity differences in China through recombination analysis, providing critical insights for enhancing the prevention and control of PRRS.

## Materials and methods

### Next-generation sequencing (NGS)

In order to investigate the genetic variation of NADC30-like PRRSVs in China over the past few years, we used the automatic alignment method in MAFFT v7.407 [27] to perform sequence alignment on the ORF5 sequences collected by our laboratory during the period from 2021 to 2023. And we constructed a phylogenetic tree by using the Maximum Likelihood (ML) method with 1,000 bootstrap replicates in IQ-TREE v1.6.12 [28–30]. Subsequently, we randomly selected 30 samples from the NADC30-like PRRSV as much as possible from different branches were subjected to next-generation sequencing (NGS) using the HiSeq platform (Illumina, San Diego, CA, USA), following the previously described protocol [31, 32]. Specific strain sequences and background information are summarized in Table S1.

### Sequence selection and recombination analysis

To screen the whole-genome sequence of all NADC30-like PRRSV strains from China in the database, we downloaded all whole-genome sequences of PRRSV in China from the GenBank. The sequences were aligned using the automatic alignment method in MAFFT v7.407 [27]. Additionally, a phylogenetic tree was constructed using the software IQ-TREE v1.6.12 [33]. Finally, we collected 224 whole-genome sequences of Chinese NADC30-like PRRSV from GenBank, which, together with the 30 whole-genome sequences determined above, a total of 254 sequences were constituted the dataset of this study. The specific information is presented in Table S2.

In accordance with prior research, five representative strains without recombination signals in lineages 1, 3, 5, and 8 were selected as reference parents. Specifically, the following strains were chosen: L1C (NADC30, Accession: JN654459.1), L1A (IA/2014/NADC34, Accession: MF326985.1), L8E (JXA1, Accession: EF112445.1), L3.5 (QYYZ, Accession: JQ308798.1), and L5A (VR2332, Accession: EF536003.1) [34–36]. Subsequently, we employed RDP software (v4.9.6) to detect potential recombination events and breakpoint locations by seven methods (RDP, GENECONV, BootScan, MasChi, Chimaera, SiScan, and 3Seq) [37]. Recombination events were confirmed when recombination signals were detected by at least four out of seven methods [35, 36]. For the strains determined to be recombinant by the RDP4 software (v4.9.6), Simplot software (v3.5.1, Baltimore, MD, USA) was further used to verify these recombination events [38]. The final determination of recombination events was made after a comprehensive evaluation of both RDP4 and SimPlot results [34, 35]. The analysis results are summarized in Table S3. The whole genome sequences of NADC30-like PRRSV obtained in this study were validated for recombination breakpoints using the Sanger sequencing method.

### Phylogenetic analysis, Nsp2 amino acid sequence and homology comparison

We performed sequence alignment using the automatic alignment method in MAFFT v7.407 [27] and constructed the phylogenetic tree with the application of IQ-TREE [33]. The resulting phylogenetic tree was annotated using the online tool Interactive Tree Of Life (iTOL) available at https://itol.embl.de/ [39].

Sequence analysis was conducted using DNASTAR software (version 18.0.3). To evaluate the Nsp2 deletion patterns of the isolates, amino acid sequence alignments were performed between the isolates and reference strains using Clustal W within the Lasergene software suite. Additionally, MEGA12 was utilized to calculate the average genetic distances both within and between each classification [40]. The recombination model diagram was created using Adobe Illustrator 2019.

### Statistical analysis

Following the geographical distribution analysis of the whole genome sequences in the statistical dataset, a map was generated and color-coded using NB MAP (https://www.nbcharts.com). All bar charts presented in this study were constructed utilizing GraphPad Prism 8 software. The Sankey diagrams were both designed and colorized using Figure Draw (https://www.figdraw.com).

## Results

### The Recombinant NADC30-like strains have increased sharply, with the L1C + L8E branch being the most predominant

**Fig. 1:**
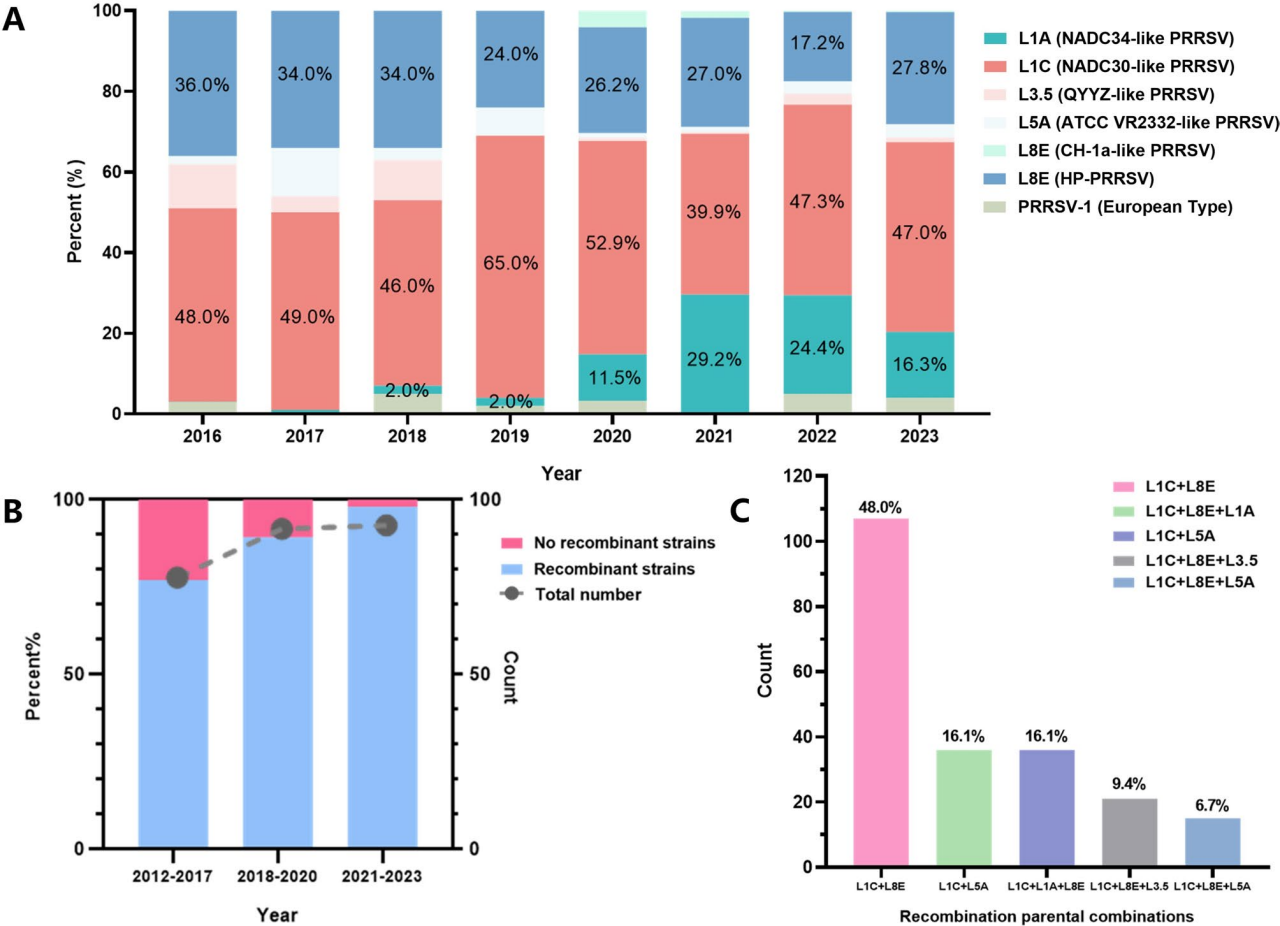
The epidemiology and recombination of NADC30-like PRRSV in China. (**A**) The positive proportion and count of NADC30-like PRRSVs detected from 2017 to 2023, based on ORF5 sequence analysis [14–19]. (**B**) Relative frequency and prevalence of recombinant NADC30-like PRRSVs over three-year intervals. (**C**) Number and proportion of distinct recombinant parental combinations of NADC30-like PRRSVs in China from 2012 to 2023

To analyze the epidemic trends of PRRSV-2 strains in China, this study plots the number and proportion of PRRSV-2 strains detected from 2016 to 2023, based on data from our laboratory’s previous research [14–19]. As depicted in Fig. 1A, since the year 2020, NADC30-like, NADC34-like, and HP-PRRSV have risen to prominence as the three dominant strains in circulation throughout China. Notably, the prevalence of NADC34-like strains has witnessed a significant increase since 2020. Meanwhile, NADC30-like strains have maintained high levels of circulation, consistently accounting for the largest proportion, thereby establishing NADC30-like PRRSV as the dominant strain in China since 2016.

To monitor the current recombination of NADC30-like strains in China, RDP4 and SimPlot software were utilized to evaluate potential recombinant events. Five prevalent clusters of strains circulating in China were selected as representative PRRSV: NADC34-like (IA/2014/NADC34), JXA1-like (JXA1), NADC30-like (NADC30), VR-2332-like (VR-2332), and QYYZ-like (QYYZ). As of 2023, only 12.20% (31 out of 254) of these strains have not undergone recombination (Table S2). The specific recombination sites and fragment sizes are detailed in Table S3.

The number of whole-genome sequences of NADC30-like PRRSV available for the periods 2012–2017, 2018–2020, and 2021–2023 remained relatively stable. However, there was a notable increase in the proportion of recombinant strains over time. Specifically, in the most recent three-year period, only 2.15% (2 out of 93) of the sequenced strains were nonrecombinant, indicating a significant rise in the frequency of recombination among NADC30-like strains, with nearly all strains exhibiting recombinant characteristics (Fig. 1B). NADC30-like strains have predominantly served as the primary parental lineage for eight distinct recombination patterns: L1C + L8E, L1C + L5A, L1C + L1A + L8E, L1C + L8E + L3.5 and L1C + L8E + L5A, with L1C + L8E being the most prevalent at 48.0% (107 out of 223) (Fig. 1C).

### Chinese NADC30-like strains were classified into 12 recombination patterns

**Fig. 2:**
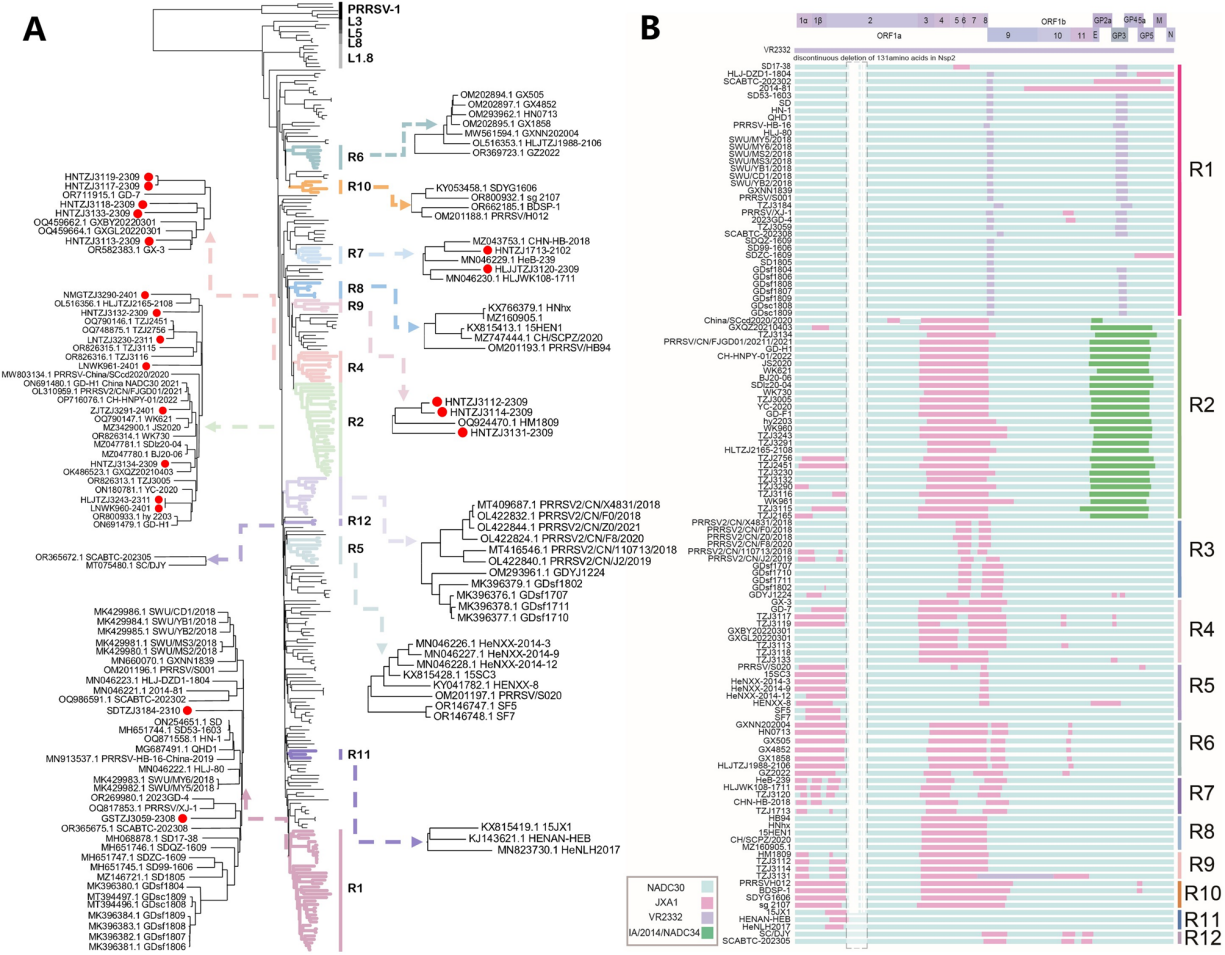
Classification system of NADC30-like PRRSVs based on phylogenetic analysis and recombination patterns. (**A**) Phylogenetic analysis of NADC30-like PRRSVs in China. The phylogenetic tree was constructed based on the complete genome sequences of NADC30-like PRRSVs and reference strains from various lineages. The whole genome sequences acquired through sequencing are denoted by  in this study. (Phylogenetic tree generated using IQ-TREE). (**B**) Genomic maps of parental lineages and recombination patterns of NADC30-like PRRSVs in China. The full-length genome structure is illustrated with reference to VR-2332, highlighting the positions and boundaries of major ORFs and NSPs within ORF1a and ORF1b. A dotted line indicates the characteristic deletion in the NSP2 hypervariable region of NADC30-like PRRSVs. (Recombination analysis performed with RDP4 and Simplot)

To characterize the genetic relationships and evolutionary dynamics of NADC30-like PRRSV over the past decade and to enhance the objectivity and reliability of their classification, a phylogenetic tree was constructed using 254 whole-genome sequences of Chinese NADC30-like strains (Fig. 2A and Figure S1). We classified the strains within the entire dataset according to their similar recombination patterns and phylogenetic clustering on the evolutionary tree. By integrating the results of maximum likelihood phylogenetic analysis with recombination analysis, we determined that 47.24% (120 out of 254) of the strains could be categorized based on their recombination modes. These strains were designated as NADC30-R1 to NADC30-R12, reflecting their epidemiological characteristics. The nonrecombinant strains were designated as NADC30-NR, comprising 12.20% (31 out of 254) of the total. Strains that could not be classified based on recombination patterns were categorized as NADC30-IR, representing 40.55% (103 out of 254) of the total (Table S2).

Based on the similarity plot analysis, we identified that NADC30-R1 contains a nonstructural protein-encoding gene region partially derived from VR2332 strain (Fig. 2B and Table S3). The characteristic recombination breakpoints of this pattern are located within the nucleotide ranges of 7,300 to 7,700 (in the Nsp9 region) and 12,000 to 12,800 (spanning the GP2a to GP3 regions). The HLJ-DZD1-1804 (MN046223.1) and SCABTC-202,302 (OQ986591.1), among others, exhibited additional recombination with JXA1 strain. The NADC30-R2 recombinant viruses possess an NADC30-like PRRSV backbone, with the Nsp3-Nsp9 gene region partially derived from JXA1 and the ORF2-ORF6 gene region partly obtained from IA/2014/NADC34. These viruses exhibit similar recombination patterns to those previously reported as L1A variants (Fig. 2B) [34]. NADC30-R3 contains two recombination fragments similar to those of JXA1 in the Nsp6-Nsp9 region. NADC30-R4 exhibits a recombination breakpoint between nucleotides 4,800 and 8,400 (spanning from Nsp3 to Nsp9). NADC30-R5 is distinguished by a recombinant fragment derived from JXA1 strain in the Nsp1-Nsp2 region. The NADC30-R6 strains was recombined with JXA1, resulting in four recombinant fragments in the Nsp1-Nsp2 and Nsp4-Nsp11 regions. NADC30-R7 features two or three recombinant segments in the Nsp1-Nsp2 region and also exhibits recombination with JXA1 in the Nsp3-Nsp9 region. The characteristic recombination breakpoints of NADC30-R8 are located within the Nsp3-Nsp9 region, derived from JXA1 strain. NADC30-R9 contains two analogous recombination fragments in the Nsp1-Nsp2 region and exhibits recombination with JXA1 in the Nsp3-Nsp9 region. NADC30-R10 generates two recombination fragments in the Nsp1-Nsp9 region that are similar to those derived from JXA1 strain. The recombination breakpoints of NADC30-R11 are located between nucleotides 1,100 and 2,200, spanning the Nsp1 to Nsp2 region. NADC30-R12 exhibits three recombination fragments within the Nsp9-ORF2b region (Fig. 2B, Table S3).

### The homology analysis, Nsp2 deletion patterns and pathogenic characteristics of each recombination pattern

The average pairwise genetic distances both between and within groups provide further evidence supporting the rationality of this classification. The intra-group genetic distances of the NADC30-R1 to R12 groups approximately 5.73% (SD ± 1.68), while the inter-group genetic distances between different groups are usually stably greater than 10%. The average genetic distance between NADC30-IR and the 13 aforementioned types exceeds 9.87% (Table 1).

**Table 1:** Average genetic distance (% nucleotide difference) within and between NADC30 strains

Subgroup	NR	IR	R1	R2	R3	R4	R5	R6	R7	R8	R9	R10	R11	R12
	*N*= 31	*N*= 103	*N* = 35	*N*= 27	*N*= 11	*N* = 9	*N* = 8	*N* = 7	*N* = 5	*N* = 5	*N* = 4	*N* = 4	*N* = 3	*N* = 2
NR	7.66													
IR	12.48	15.93												
R1	8.61	13.18	6.53											
R2	12.04	14.76	12.69	6.68										
R3	8.50	12.62	9.36	12.02	5.98									
R4	11.57	14.46	12.37	9.48	11.53	7.77								
R5	9.33	13.08	10.26	12.22	9.82	11.58	6.59							
R6	11.59	14.07	12.48	12.26	10.66	11.34	10.32	4.15						
R7	10.43	13.53	11.43	12.62	10.44	11.54	10.60	10.59	6.58					
R8	9.15	12.72	10.33	9.85	9.35	9.64	10.08	10.35	10.44	4.56				
R9	11.70	14.54	12.58	11.62	11.69	11.32	11.51	11.12	11.86	9.58	7.21			
R10	11.84	14.06	12.68	11.73	11.28	10.98	10.15	9.08	10.60	9.78	10.53	4.75		
R11	7.86	12.45	9.08	11.90	8.75	11.20	8.05	10.87	10.21	8.58	10.92	10.83	6.58	
R12	9.01	13.05	9.75	12.58	9.10	11.56	10.29	11.47	9.61	10.20	12.08	11.57	9.18	1.39

Amino acid sequence alignment revealed that, in comparison to VR-2332, the NADC30-R1 to NADC30-R12 variant exhibited a discontinuous deletion of 131 amino acids (comprising 111 + 1 + 19 residues) in Nsp2. This deletion pattern is similar to that observed in the NADC30 strain and specifically occurs at positions 322–432, 483, and 504–522. However, we did not identify any distinctive and consistent features within the Nsp2 region across the different classifications. Despite the frequent occurrence of recombination events, all NADC30-R1 to NADC30-R12 variants retained the characteristic 131-amino-acid deletion associated with NADC30.

To investigate potential patterns in the pathogenicity of different recombinant strains, this study systematically analyzed and summarized the pathogenic characteristics of NADC30-like strains (Table 2 and Table S4). Due to the lack of clear standards in pathogenicity research, any strain causing death in infected pigs is classified as highly pathogenic. Strains are considered moderately pathogenic if infected piglets exhibit fever along with pathological (histopathological) lesions. If infected piglets show no obvious clinical symptoms, the strain is classified as having low pathogenicity. The pathogenicity of NADC30-like PRRSVs varies from low to high, with most strains exhibiting moderate virulence (Table 2 and Table S4). Our analysis did not reveal entirely consistent virulence characteristics among the NADC30-R1 to R12 strains. Specifically, the following strains demonstrated high pathogenicity: SD17-38 (NADC30-R1), SD1805 (NADC30-R1), SCcd2020 (NADC30-R2), GXQZ20210403 (NADC30-R2), GD-7 (NADC30-R4), and HNhx (NADC30-R8). The remaining strains exhibited moderate pathogenicity.

**Table 2:** Comparison of pathogenicity of different NADC30-like strains

Infected PRRSV strain	Subtype	The days of inoculation (dpi)	Inoculated dose	Parameters of evaluation	Challenge group	Pathogenicity	Reference
SD53-1603	R1	18	8 × 10^4^ TCID_50_	Clinical symptoms	Medium clinical symptoms	Moderate	[16]
				Days of fever	12 days(≥ 40.5℃)		
				Pathological and histopathological lesions	Obvious pathological changes		
				Viremia	Peaked at 5 dpi, longer than 21 days		
SD17-38	R1	21	2 × 10^5^ TCID_50_	Clinical symptoms	Severe clinical symptoms, 2/5 pigs died	High	[50]
				Days of fever	21 days(≥ 40℃)		
				Pathological and histopathological lesions	Obvious pathological changes		
				Viremia	Peaked at 11 dpi, longer than 21 days		
SD1805	R1	14	1 × 10^5.5^ TCID_50_	Clinical symptoms	Severe clinical symptoms, 1/5 pigs died	High	[51]
				Days of fever	1 day(≥ 40℃)		
				Pathological and histopathological lesions	Obvious pathological changes		
				Viremia	Peaked at 4 dpi, longer than 14 days		
SCcd2020	R2	14	2 × 10^9.19^ TCID_50_	Clinical symptoms	Severe clinical symptoms, 3/5 pigs died	High	[52]
				Days of fever	12 days(≥ 40℃)		
				Pathological and histopathological lesions	Obvious pathological changes		
				Viremia	Peaked at 10 dpi, longer than 14 days		
TZJ2756	R2	21	4 × 10^5^ TCID_50_	Clinical symptoms	Mild clinical symptoms	Moderate	[34]
				Days of fever	3–10 days(≥ 40℃)		
				Pathological and histopathological lesions	Mild pathological changes		
				Viremia	Peaked at 3 dpi, longer than 21 days		
GD-H1	R2	14	1 × 10^5^ TCID_50_	Clinical symptoms	Mild clinical symptoms, Pregnant Sows 1/4 died and 2/4 miscarriage, no piglets died	High	[53]
				Days of fever	3–12 days(≥ 40℃)		
				Pathological and histopathological lesions	Slightly pathogenic to piglets, but highly pathogenic to pregnant sows		
				Viremia	Started at 3 dpi, longer than 14 days		
GD-F1	R2	14	1 × 10^5^ TCID_50_	Clinical symptoms	Severe pathological changes Pregnant Sows 2/4 died and 1/4 miscarriage	High	[53]
				Days of fever	3–12 days(≥ 40℃)		
				Pathological and histopathological lesions	Slightly pathogenic to piglets, but highly pathogenic to pregnant sows		
				Viremia	Started at 3 dpi, longer than 14 days		
YC-2020	R2	14	4 × 10^5^ TCID_50_	Clinical symptoms	Mild clinical symptoms	Moderate	[54]
				Days of fever	2 days(≥ 40℃)		
				Pathological and histopathological lesions	Mild pathological changes		
				Viremia	-		
GXQZ20210403	R2	14	2 × 10^4^ TCID_50_	Clinical symptoms	Severe clinical symptoms, 1/5 pigs died	High	[55]
				Days of fever	4 days(≥ 40℃)		
				Pathological and histopathological lesions	Obvious pathological changes		
				Viremia	Peaked at 7 dpi, longer than 14 days		
GX-3	R4	14	2 × 10^5^ TCID_50_	Clinical symptoms	Mild clinical symptoms	Moderate	[56]
				Days of fever	7 days(≥ 40℃)		
				Pathological and histopathological lesions	Mild pathological changes		
				Viremia	Peaked at 7 dpi, longer than 14 days		
GD-7	R4	14	2 × 10^5^ TCID_50_	Clinical symptoms	Severe clinical symptoms,2/6 pigs died	High	[56]
				Days of fever	12 days(≥ 40℃)		
				Pathological and histopathological lesions	Obvious pathological changes		
				Viremia	Peaked at 7 dpi, longer than 14 days		
HLJWK108-1711	R7	21	1 × 10^5^ TCID_50_	Clinical symptoms	Mild clinical symptoms	Moderate	[57]
				Days of fever	12 days(≥ 40℃)		
				Pathological and histopathological lesions	Mild pathological changes		
				Viremia	Peaked at 5 dpi, longer than 21 days		
CHN-HB-2018	R7	21	3 × 10^6.5^ TCID_50_	Clinical symptoms	Mild clinical symptoms	Moderate	[29]
				Days of fever	8 days(≥ 40℃)		
				Pathological and histopathological lesions	Mild clinical symptoms		
				Viremia	Peaked at 14 dpi, longer than 21 days		
HNhx	R8	28	2 × 10^5^ TCID_50_	Clinical symptoms	Severe clinical symptoms,3/5 pigs died	High	[58]
				Days of fever	14 days(≥ 40℃)		
				Pathological and histopathological lesions	Obvious pathological changes		
				Viremia	Peaked at 17 dpi, longer than 21 days		
SDbz16-2	R8	14	2.5 × 10^6^ TCID_50_	Clinical symptoms	Mild clinical symptoms	Moderate	[59]
				Days of fever	3 days(≥ 40℃)		
				Pathological and histopathological lesions	Mild pathological changes		
				Viremia	Peaked at 7 dpi, longer than14 days		
SCABTC-202,309	R12	15	4 × 10^5^ TCID_50_	Clinical symptoms	Mild clinical symptoms	Moderate	[23]
				Days of fever	5 days(≥ 40℃)		
				Pathological and histopathological lesions	Mild pathological changes		
				Viremia	Peaked at 9 dpi, longer than 15 days		

### NADC30-R1 and NADC30-R2 have become widely prevalent in certain regions of China

**Fig. 3:**
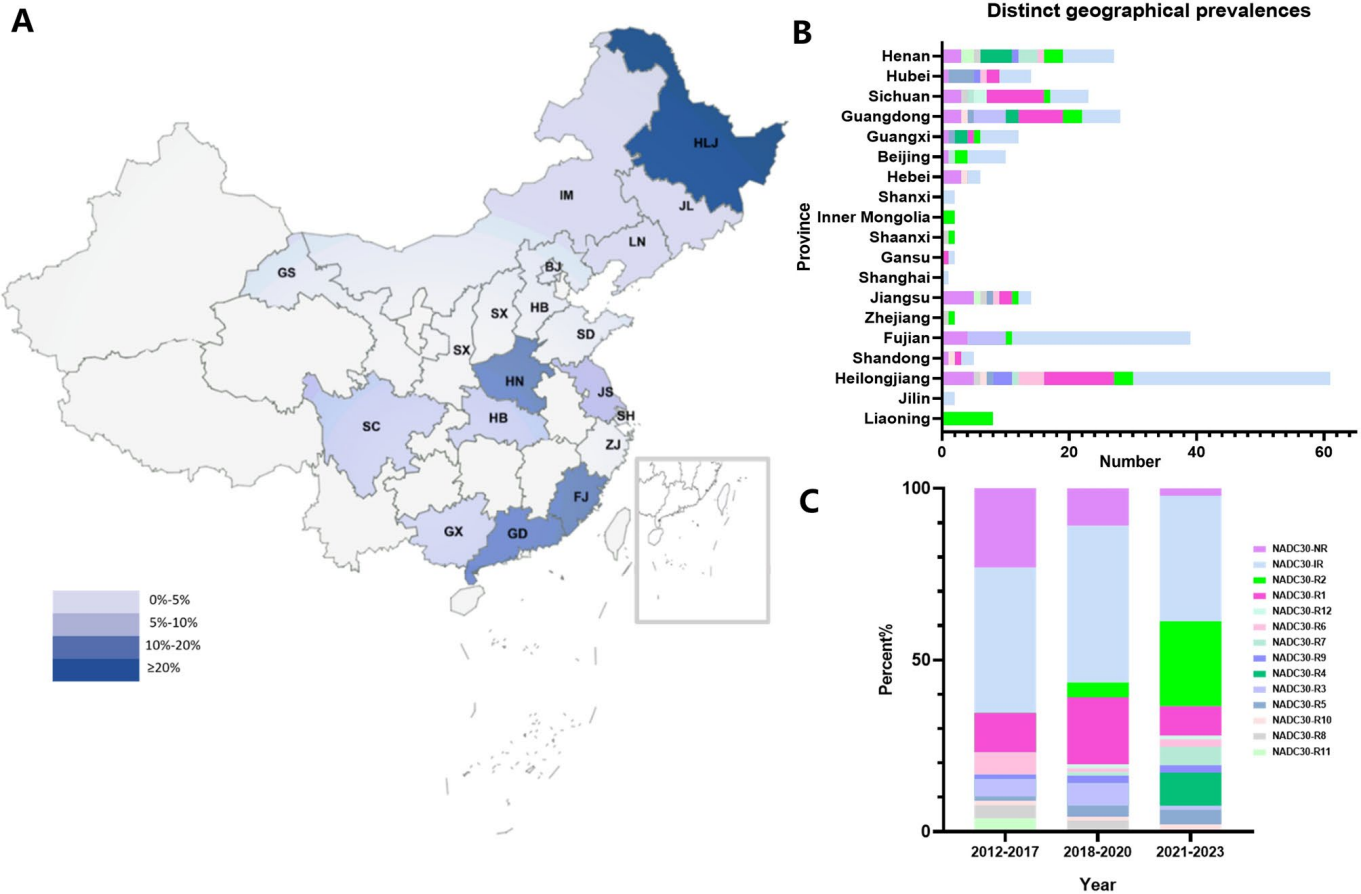
Geographical distribution and proportion of NADC30-like PRRSV whole genome sequences in each group. (**A**) Geographical distribution of NADC30-like PRRSV whole genome sequences across various regions in China. (**B**) The number of distinct NADC30 strains identified in different provinces of China. (**C**) The proportion of NADC30-R1 to R12 strains over varying time periods from 2012 to 2023

We performed a comprehensive statistical analysis of the geographical distribution of 254 NADC30-like strains based on their background information. The results demonstrated that the whole genomes of NADC30-like PRRSV have been sequenced from 19 provinces in China, include Heilongjiang, Fujian, Guangdong, Henan, Sichuan, Jiangsu, Hubei, Guangxi, Beijing, Liaoning, Hebei, Shandong, Jilin, Gansu, Shaanxi, Inner Mongolia, Shanxi, Zhejiang, and Shanghai (Fig. 3A and B). Additionally, Heilongjiang, Fujian, Guangdong and Henan, accounted for 23%, 16%, 12% and 10%, respectively of NADC30-like PRRSV isolates with whole-genome sequences in China (Fig. 3A and B). Notably, after 2018, the number of strains classified as NADC30-R1 ~ R12 (with consistent recombination characteristics) increased (Fig. 3C).

**Fig. 4:**
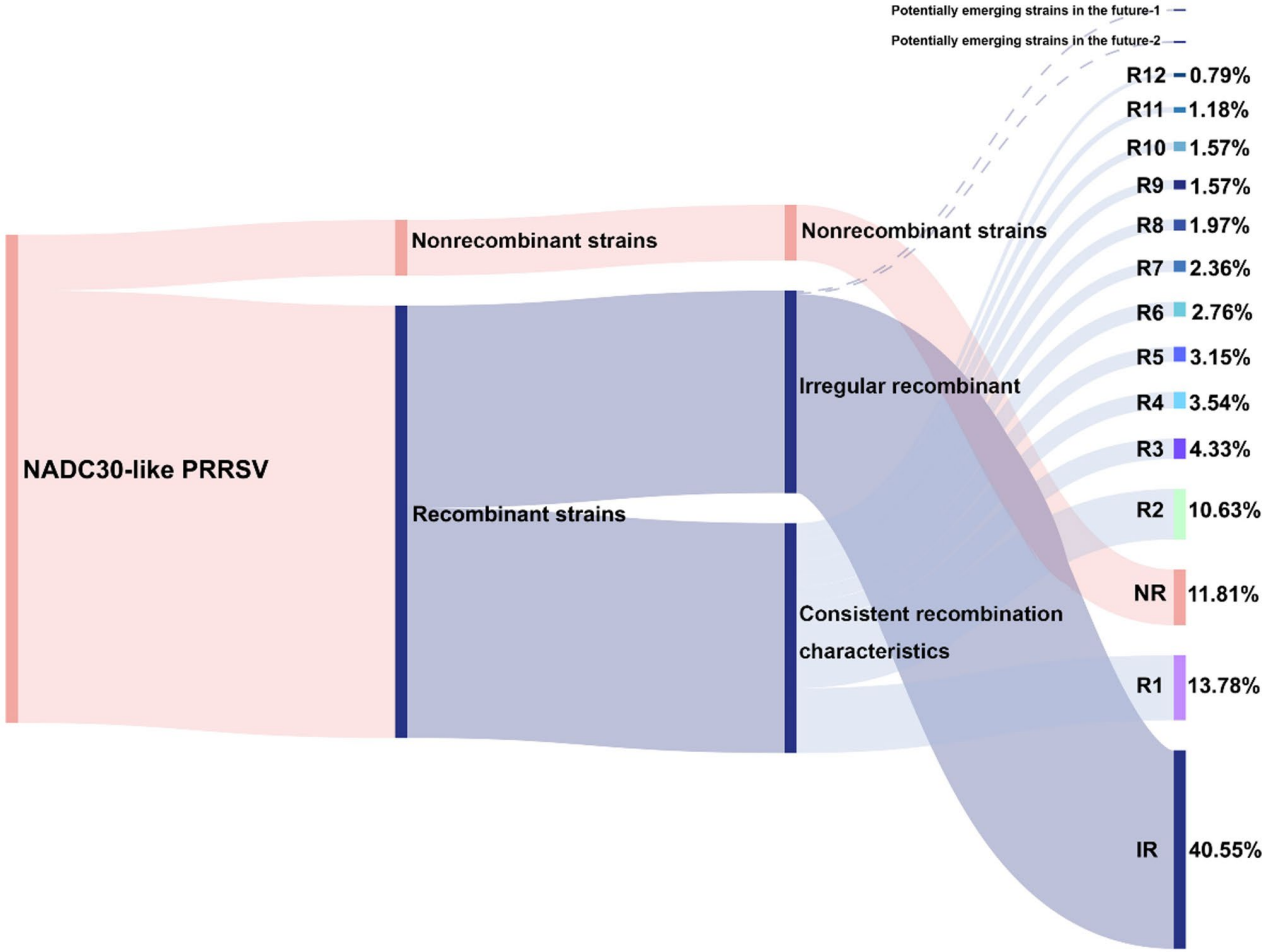
The proportion of NADC30-like PRRSV with different recombination patterns in China. NR: nonrecombinant strains; IR: strains with irregular recombination; R1 to R12: recombinant strains with consistent characteristics. The X-axis represents the classification criteria of this study. The NADC30-like strains in China are divided into nonrecombinant strains and recombinant strains. The recombinant strains are further classified into NADC30-R (recombinant; R1-R12) and NADC30-IR (irregular recombinant). The Y-axis represents the sample sizes of different types

As illustrated in Fig. 4, the NADC30-R1 strains comprised the largest proportion at 13.78% (35 out of 254 cases) and is predominantly found in provinces such as Heilongjiang, Sichuan, and Guangdong. These strains were initially identified in 2014 and have since been circulating for an extended period. In recent years, these strains have consistently been reported in China, necessitating ongoing attention from researchers. The NADC30-R2 variant, which accounted for 10.63% (27 out of 254 cases) (Fig. 4), is the most widely distributed type of NADC30-like PRRSV in China, and has been distributed in at least 12 provinces (Fig. 3B). This pattern first emerged in 2020 and subsequently spread rapidly, demonstrating a steadily increasing detection rate (Fig. 3B). With a consistent L1A + L1C + L8E recombination profile, this pattern exhibits potential epidemic characteristics. These findings suggest that the recombination dynamics of NADC30-like strains in China remain complex; however, certain genotypes sharing identical recombination patterns have spread rapidly in some regions of the country.

## Discussion

PRRS is among the most significant infectious diseases impacting the global pig industry, resulting in considerable economic losses [41]. The PRRSV-2 circulating in China can mainly be classified into four sublineages: L1A, L1C, L3.5 and L8E. Notably, the sublineage L1C (NADC30-like) has become the dominant strain in the country since 2016.

Our recombination analysis has demonstrated a marked rise in the prevalence of recombinant NADC30-like strains in recent years. The ongoing occurrence of recombination events has significantly contributed to the genetic diversity of PRRSV in China, whereas non-recombinant strains have become increasingly rare. Among the recombinant strains, L1C + L8E, L1C + L5A and L1C + L1A + L8E are the three most common patterns, of which L1C + L8E pattern accounts for the highest proportion, reaching 48.0% (107 out of 223). This pronounced recombination preference may contribute significantly to the sustained prevalence of NADC30-like strains. These findings are consistent with previous studies by Yu et al. and Zhao et al. [42, 43], which reported that strains from lineages 1 and 8 exhibit a higher propensity for recombination events compared to other lineages. Studies have shown that recombination fragments of PRRSV gradually accumulate [44], leading to increasingly complex recombination patterns. We speculate that in the future, the proportion of strains with three sublineages or even more complex recombination patterns will further increase.

With the proliferation of recombinant strains, relying solely on ORF5 for typing has become insufficiently rigorous. Consequently, we conducted phylogenetic analysis using whole-genome sequencing and integrated the findings from recombination analysis to classify NADC30-like strains in China. Our classification divides NADC30-like PRRSVs in China into three main categories: NADC30-R (recombinant; R1-R12), NADC30-NR (nonrecombinant), and NADC30-IR (irregular recombinant). The intra-group genetic distances of the NADC30-R1 to R12 groups approximately 5.73% (SD ± 1.68), while the inter-group genetic distances between different groups are usually stably greater than 10%. This further corroborates the high reliability of our classification results. In addition to recombination, Nsp2 polymorphism is another significant contributor to the high variability of the PRRSV genome [45]. Despite frequent recombination events, the NADC30-R1 ~ NADC30-R12 variant all retained the 131-amino-acid deletion characteristic of NADC30 strain. Previous studies have shown that Nsp2 also present on or within the virions [46]. Meanwhile, it is also closely related to the virulence, cellular tropism and viral assembly of PRRSV [47–49]. The role of 131-amino-acid deletion pattern in the PRRSV life cycle remains to be further explored.

Although we observed that the NADC30-R1 ~ R12 strains do not exhibit entirely consistent virulence characteristics [29, 50–68], these highly pathogenic NADC30-like strains all underwent recombination in the Nsp9 region. The PRRSV ORF1b encodes Nsp9, an RNA-dependent RNA polymerase (RdRp), which has been identified as a key virulence factor of HP-PRRSV in vivo [69]. However, not all strains that experienced recombination events in this region exhibited high pathogenicity. For instance, YC-2020 (NADC30-R2) [55] and GX-3 (NADC30-R4) [57] (Table 2) did not display heightened virulence. In summary, the pathogenicity tests indicate that virulence may be associated with specific recombination patterns. Nevertheless, variations in pathogenicity within the same category also suggest that recombination patterns alone are not the sole determinant of pathogenicity.

Whole genome sequences of NADC30-like strains have been sequenced by researchers across 19 provinces in China. Notably, provinces such as Heilongjiang, Fujian, Guangdong, Henan, and Sichuan account for a relatively large proportion of these sequences, possibly due to the high density of pig farming and the presence of numerous research institutions in these regions. According to GenBank data, the NADC30-R1 strain was first detected in 2014 and has been circulating in China for over a decade. Despite a declining detection frequency since 2018, NADC30-R1 remains the most numerous type, accounting for 15.70% (35 out of 223 cases). Our laboratory has consistently detected this variant in recent years (Table S2). In 2023, the sublineage L1A, L1C, and L8E collectively accounted for 92.7% of the PRRSV-2 positive samples. The NADC30-R2 strains happens to be a recombinant virus derived from L1A + L1C + L8E strains, also known as the L1A variant. First identified in 2020, this strain had disseminated across 12 provinces in China by 2023, establishing itself as the most extensively distributed type, accounting for 12.11% (27 out of 223 cases) and indicating its potential as an epidemic strain. After 2018, the proportion of Chinese NADC30-like PRRSV strains sharing the same recombination pattern continued to increase, leading us to speculate that an even larger proportion of strains may be classifiable based on recombination characteristics in the future. Researchers in China should enhance surveillance efforts targeting strains with similar recombination characteristics [34].

The epidemiological situation of NADC30-like PRRSV in China remains complex. A notable trend is the continuous emergence of recombinants, which has significantly increased the genetic diversity of PRRSV in China. It is also important to highlight that NADC30-IR remains the largest group at present. The recombination patterns within this group are relatively diverse and lack consistent characteristics. As the number of full-length sequences in the GenBank database continues to grow, it is plausible that new circulating strains may emerge, as well as potential reclassification into existing categories (NADC30-R1 ~ NADC30-R12) (Fig. 4). This study represents the first comprehensive investigation into the genetic evolution and prevalence of NADC30-like PRRSV from 2012 to 2023, incorporating recombinant analysis. It elucidates the variation and evolutionary trends of epidemic PRRSV strains, providing valuable insights for future prevention and control strategies.

## Conclusion

In summary, NADC30-like strains remain the predominant genotype in China, and according to the statistical results of this study, nonrecombinant strains are rapidly declining. Among recombination events, those involving L1C + L8E as parental strains are most prevalent. By integrating phylogenetic and recombination analyses, we have, for the first time, classified Chinese NADC30-like strains into twelve distinct recombinant types, designated NADC30-R1 to NADC30-R12. Each strain type exhibits a consistent recombination pattern, with NADC30-R1 and NADC30-R2 being the most widespread and abundant, indicating that they have established localized epidemics in certain regions. These findings will enhance our surveillance of predominant PRRSV epidemics in China and contribute to improved preventive and control measures against PRRS.

## Electronic supplementary material

Below is the link to the electronic supplementary material.

**Supplementary Material 1: Fig. S1.** Phylogenetic analysis of NADC30-like PRRSVs in China

**Supplementary Material 2: Table S1.** Information of NADC30-like PRRSV sequences detected by this study

**Supplementary Material 3: Table S2.** Information about NADC30-like PRRSV reference strains used in this study

**Supplementary Material 4: Table S3.** Information on recombination events of NADC30-like PRRSV detected by RPD4 software

**Supplementary Material 5: Table S4.** Comparison of pathogenicity of different NADC30-like strains

## Data Availability

No datasets were generated or analysed during the current study.
