# Supplementary material for: Genomic characteristics and epidemic trends of NADC30-like PRRSV in China
Source: Porcine Health Manag. 2025 May 28;11:30. doi: 10.1186/s40813-025-00444-7 (PMC12121172; doi:10.1186/s40813-025-00444-7)
Supplement: Supplementary file 1 — Supplementary Material 1: Fig. S1. Phylogenetic analysis of NADC30-like PRRSVs in China [file 40813_2025_444_MOESM1_ESM.docx]

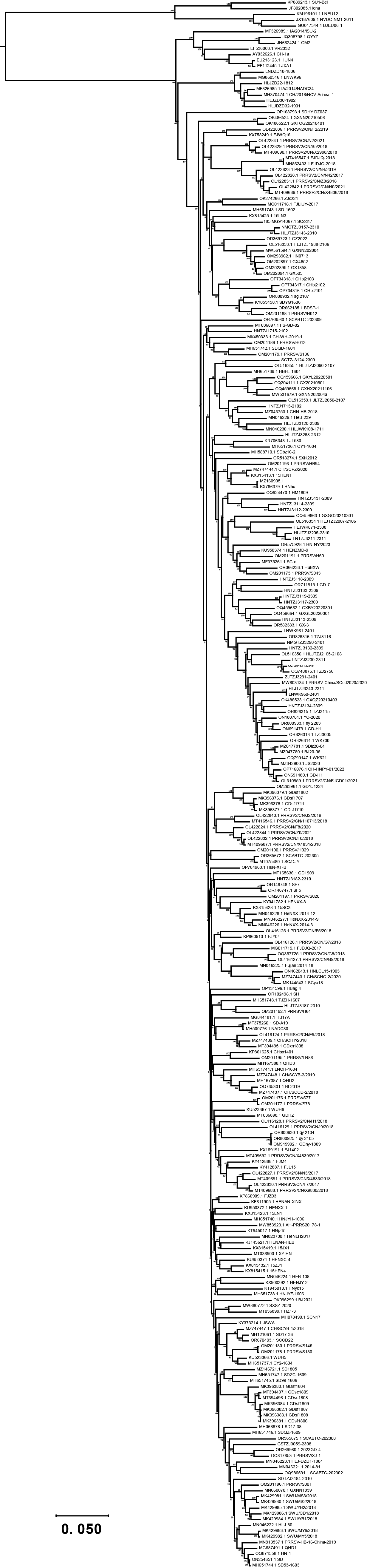


FIG S1. Phylogenetic analysis of NADC30-like PRRSVs in China. The phylogenetic tree was constructed based on the complete genome sequences of Chinese NADC30-like PRRSVs and reference strains from various lineages. (Phylogenetic tree generated using IQ-TREE and was annotated using the online tool Interactive Tree Of Life (iTOL) available at https://itol.embl.de/)
