# Supplementary material for: Genomic characteristics and epidemic trends of NADC30-like PRRSV in China
Source: Porcine Health Manag. 2025 May 28;11:30. doi: 10.1186/s40813-025-00444-7 (PMC12121172; doi:10.1186/s40813-025-00444-7)
Supplement: Supplementary file 3 — Supplementary Material 3: Table S2. Information about NADC30-like PRRSV reference strains used in this study [file 40813_2025_444_MOESM3_ESM.docx]

**TABLE** **S2 Information about NADC30-like PRRSV reference strains used in this study.**

| Taxa | GenBank accession no. | Minor parental strains | Place of isolation | Year of isolation |
| --- | --- | --- | --- | --- |
| NADC30-R1 13.78% (35/254) | | | | |
| SD17-38 | MH068878.1  6880 | VR2332/JXA1 | Jiangsu | 2018 |
| HLJ-DZD1-1804 | MN046223.1 | VR2332/JXA1 | Heilongjiang | 2018 |
| SCABTC-202302 | OQ986591.1 | VR2332/JXA1 | Sichuan | 2022 |
| 2014-81 | MN046221.1 | VR2332/JXA1 | Heilongjiang | 2014 |
| SD53-1603 | MH651744.1 | VR2332 | Heilongjiang | 2016 |
| SD | ON254651.1 | VR2332 | Heilongjiang | 2016 |
| HN-1 | OQ871558.1 | VR2332 | Hubei | 2021 |
| QHD1 | MG687491.1 | VR2332 | Hubei | 2017 |
| PRRSV-HB-16-China-2019 | MN913537.1 | VR2332 | Jilin | 2019 |
| HLJ-80 | MN046222.1 | VR2332 | Heilongjiang | 2016 |
| SWU/MY5/2018 | MK429982.1 | VR2332 | Sichuan | 2018 |
| SWU/MY6/2018 | MK429983.1 | VR2332 | Sichuan | 2018 |
| SWU/MS2/2018 | MK429980.1 | VR2332 | Sichuan | 2018 |
| SWU/MS3/2018 | MK429981.1 | VR2332 | Sichuan | 2018 |
| SWU/YB1/2018 | MK429984.1 | VR2332 | Sichuan | 2018 |
| SWU/CD1/2018 | MK429986.1 | VR2332 | Sichuan | 2018 |
| SWU/YB2/2018 | MK429985.1  1 | VR2332 | Sichuan | 2018 |
| GXNN1839 | MN660070.1 | VR2332 | Guangxi | 2018 |
| PRRSV/S001 | OM201196.1 | VR2332 | Heilongjiang | 2018 |
| SDTZJ3184-2310 |  | VR2332 | Shandong | 2023 |
| PRRSV/XJ-1 | OQ817853.1 | VR2332/JXA1 | Heilongjiang | 2021 |
| 2023GD-4 | OR269980.1 | VR2332/JXA1 | Jiangsu | 2023 |
| GSTZJ3059-2308 |  | VR2332 | Gansu | 2023 |
| SCABTC-202308 | OR365675.1 | VR2332 | Sichuan | 2023 |
| SDQZ-1609 | MH651746.1 | VR2332 | Heilongjiang | 2016 |
| SD99-1606 | MH651745.1 | VR2332 | Heilongjiang | 2016 |
| SDZC-1609 | MH651747.1 | VR2332/JXA1 | Heilongjiang | 2016 |
| SD1805 | MZ146721.1 | VR2332 | Heilongjiang | 2022 |
| GDsf1804 | MK396380.1 | VR2332 | Guangdong | 2018 |
| GDsf1806 | MK396381.1 | VR2332 | Guangdong | 2018 |
| GDsf1808 | MK396383.1 | VR2332 | Guangdong | 2018 |
| GDsf1807 | MK396382.1 | VR2332 | Guangdong | 2018 |
| GDsf1809 | MK396384.1 | VR2332 | Guangdong | 2018 |
| GDsc1808 | MT394496.1 | VR2332 | Guangdong | 2018 |
| GDsc1809 | MT394497.1 | VR2332 | Guangdong | 2018 |
| NADC30-R2 10.63% (27/254) | | | | |
| LNWK961-2401 |  | NADC34/JXA1 | Liaoning | 2024 |
| HNTZJ3132-2309 |  | NADC34/JXA1 | Henan | 2023 |
| TZJ3116 | OR826316.1 | NADC34/JXA1 | Heilongjiang | 2023 |
| NMGTZJ3290-2401 |  | NADC34/JXA1 | Inner Mongolia Autonomous Region | 2024 |
| HLJTZJ2165-2108 | OL516356.1 | NADC34/JXA1 | Heilongjiang | 2021 |
| PRRSV-China/SCcd2020/2020 | MW803134.1 | NADC34/JXA1 | Sichuan | 2020 |
| WK730 | OR826314.1 | NADC34/JXA1 | Liaoning | 2023 |
| WK621 | OQ790147.1 | NADC34/JXA1 | Inner Mongolia Autonomous Region | 2021 |
| JS2020 | MZ342900.1 | NADC34/JXA1 | Jiangsu | 2020 |
| ZJTZJ3291-2401 |  | NADC34/JXA1 | Zhejiang | 2024 |
| CH-HNPY-01/2022 | OP716076.1 | NADC34/JXA1 | Henan | 2022 |
| GD-H1 | ON691479.1 | NADC34/JXA1 | Guangdong | 2021 |
| PRRSV2/CN/FJGD01/2021 | OL310959.1 | NADC34/JXA1 | Fujian | 2021 |
| TZJ3005 | OR826313.1 | NADC34/JXA1 | Liaoning | 2023 |
| YC-2020 | ON180781.1 | NADC34/JXA1 | Shanxi | 2021 |
| HLJTZJ3243-2311 |  | NADC34/JXA1 | Heilongjiang | 2023 |
| LNWK960-2401 |  | NADC34/JXA1 | Liaoning | 2024 |
| hy_2203 | OR800933.1 | NADC34/JXA1 | Guangdong | 2023 |
| GD-H1 | ON691480.1 | NADC34/JXA1 | Guangdong | 2021 |
| SDlz20-04 | MZ047781.1 | NADC34/JXA1 | Beijing | 2020 |
| BJ20-06 | MZ047780.1 | NADC34/JXA1 | Beijing | 2020 |
| HNTZJ3134-2309 |  | NADC34/JXA1 | Henan | 2023 |
| GXQZ20210403 | OK486523.1 | NADC34/JXA1 | Guangxi | 2021 |
| LNTZJ3230-2311 |  | NADC34/JXA1 | Liaoning | 2023 |
| TZJ2756 | OQ748875.1 | NADC34/JXA1 | Liaoning | 2022 |
| TZJ2451 | OQ790146.1 | NADC34/JXA1 | Liaoning | 2022 |
| TZJ3115 | OR826315.1 | NADC34/JXA1 |  | 2023 |
| NADC30-R3 4.33% (11/254) | | | | |
| GDsf1711 | MK396378.1 | JXA1 | Guangdong | 2017 |
| GDsf1710 | MK396377.1 | JXA1 | Guangdong | 2017 |
| GDsf1707 | MK396376.1 | JXA1 | Guangdong | 2017 |
| GDsf1802 | MK396379.1 | JXA1 | Guangdong | 2018 |
| GDYJ1224 | OM293961.1 | JXA1 | Guangdong | 2020 |
| PRRSV2/CN/X4831/2018 | MT409687.1 | JXA1 | Fujian | 2018 |
| PRRSV2/CN/F0/2018 | OL422832.1 | JXA1 | Fujian | 2018 |
| PRRSV2/CN/Z0/2021 | OL422844.1 | JXA1 | Fujian | 2021 |
| PRRSV2/CN/F8/2020 | OL422824.1 | JXA1 | Fujian | 2020 |
| PRRSV2/CN/J2/2019 | OL422840.1 | JXA1 | Fujian | 2019 |
| PRRSV2/CN/110713/2018 | MT416546.1 | JXA1 | Fujian | 2017 |
| NADC30-R4 3.54% (9/254) | | | | |
| HNTZJ3133-2309 |  | JXA1 | Henan | 2023 |
| HNTZJ3118-2309 |  | JXA1 | Henan | 2023 |
| GXBY20220301 | OQ459662.1 | JXA1 | Guangxi | 2022 |
| GXGL20220301 | OQ459664.1 | JXA1 | Guangxi | 2022 |
| GX-3 | OR582383.1 | JXA1 | Guangdong | 2023 |
| HNTZJ3113-2309 |  | JXA1 | Henan | 2023 |
| HNTZJ3119-2309 |  | JXA1 | Henan | 2023 |
| HNTZJ3117-2309 |  | JXA1 | Henan | 2023 |
| GD-7 | OR711915.1 | JXA1 | Guangdong | 2023 |
| NADC30-R5 3.15% (8/254) | | | | |
| HeNXX-2014-3 | MN046226.1 | JXA1 | Heilongjiang | 2014 |
| HeNXX-2014-9 | MN046227.1 | JXA1 | Heilongjiang | 2014 |
| HeNXX-2014-12 | MN046228.1 | JXA1 | Heilongjiang | 2014 |
| 15SC3 | KX815428.1 | JXA1 | Jiangsu | 2015 |
| HeNXX-8 | KY041782.1 | JXA1 | Henan | 2016 |
| SF5 | OR146747.1 | JXA1 | Hubei | 2022 |
| SF7 | OR146748.1 | JXA1 | Hubei | 2022 |
| PRRSV/S020 | OM201197.1 | JXA1 | Heilongjiang | 2018 |
| NADC30-R6 2.76% (7/254) | | | | |
| GX505 | OM202894.1 | JXA1 | Hubei | 2020 |
| GX4852 | OM202897.1 | JXA1 | Hubei | 2021 |
| HN0713 | OM293962.1 | JXA1 | Guangdong | 2021 |
| GX1858 | OM202895.1 | JXA1 | Hubei | 2020 |
| GXNN202004 | MW561594.1 | JXA1 | Guangxi | 2020 |
| HLJTZJ1988-2106 | OL516353.1 | JXA1 | Heilongjiang | 2021 |
| GZ2022 | OR369723.1 | JXA1 | Hubei | 2022 |
| NADC30-R7 1.97% (5/254) | | | | |
| HeB-239 | MN046229.1 | JXA1 | Heilongjiang | 2018 |
| HLJJTZJ3120-2309 |  | JXA1 | Heilongjiang | 2023 |
| HLJWK108-1711 | MN046230.1 | JXA1 | Heilongjiang | 2017 |
| HNTZJ1713-2102 |  | JXA1 | Henan | 2021 |
| CHN-HB-2018 | MZ043753.1 | JXA1 | Hubei | 2018 |
| NADC30-R8 1.97% (5/254) | | | | |
| HNhx | KX766379.1 | JXA1 | Henan | 2016 |
|  | MZ160905.1 | JXA1 | Shananxi | 2020 |
| 15HEN1 | KX815413.1 | JXA1 | Jiangsu | 2015 |
| CH/SCPZ/2020 | MZ747444.1 | JXA1 | Sichuan | 2020 |
| PRRSV/HB94 | OM201193.1 | JXA1 | Heilongjiang | 2019 |
| NADC30-R9 1.57% (4/254) | | | | |
| HM1809 | OQ924470.1 | JXA1 | Beijing | 2018 |
| HNTZJ3131-2309 |  | VR2332/JXA1 | Henan | 2023 |
| HNTZJ3112-2309 |  | JXA1 | Henan | 2023 |
| HNTZJ3114-2309 |  | JXA1 | Henan | 2023 |
| NADC30-R10 1.57% (4/254) | | | | |
| SDYG1606 | KY053458.1 | JXA1 | Shandong | 2016 |
| sg_2107 | OR800932.1 | JXA1 | Guangdong | 2021 |
| PRRSV/H012 | OM201188.1 | JXA1 | Heilongjiang | 2018 |
| BDSP-1 | OR662185.1 | JXA1 | Hebei | 2023 |
| NADC30-R11 1.18% (3/254) | | | | |
| 15JX1 | KX815419.1 | JXA1 | Jiangsu | 2015 |
| HENAN-HEB | KJ143621.1 | JXA1 | Henan | 2012 |
| HeNLH2017 | MN823730.1 | JXA1 | Henan | 2017 |
| NADC30-R12 0.79% (2/254) | | | | |
| SC/DJY | MT075480.1 | JXA1 | Sichuan | 2019 |
| SCABTC-202305 | OR365672.1 | JXA1 | Sichuan | 2022 |
| NADC30-NR 12.20% (31/254) | | | | |
| QHD2 | MH167387.1 | - | Hebei | 2017 |
| 15HEN4 | KX815415.1 | - | Jiangsu | 2015 |
| 15ZJ1 | KX815432.1 | - | Jiangsu | 2015 |
| HENXX-1 | KU950372.1 | - | Henan | 2014 |
| NADC30 | MH500776.1 | - | Shandong | 2017 |
| SD-A19 | MF375260.1 | - | Heilongjiang | 2015 |
| FJZ03 | KP860909.1 | - | Fujian | 2015 |
| FJZ04 | KP860910.1 | - | Fujian | 2015 |
| CH/SCYB-1/2018 | MZ747447.1 | - | Sichuan | 2018 |
| SCCD22 | OR670493.1 | - | Sichuan | 2022 |
| HZ1-3 | MT036899.1 | - | Guangdong | 2017 |
| SXSZ-2020 | MW880772.1 | - | Beijing | 2020 |
| SD17-36 | MH121061.1 | - | Jiangsu | 2017 |
| HNjz15 | KT945017.1 | - | Henan | 2015 |
| GDxn1808 | MT394495.1 | - | Guangdong | 2018 |
| CH/SCHY/2018 | MZ747439.1 | - | Sichuan | 2018 |
| PRRSV2/CN/E9/2018 | OL416124.1 | - | Fujian | 2018 |
| TJZH-1607 | MH651748.1 | - | Heilongjiang | 2016 |
| PRRSV/H64 | OM201192.1 | - | Heilongjiang | 2019 |
| HB17A | MG844181.1 | - | Hebei | 2017 |
| XY-HN | MT036900.1 | - | Guangdong | 2017 |
| GXYL20220501 | OQ459666.1 | - | Guangxi | 2022 |
| QHD3 | MH167388.1 | - | Hebei | 2017 |
| PRRSV2/CN/H1/2018 | OL416128.1 | - | Fujian | 2019 |
| PRRSV/S77 | OM201176.1 | - | Heilongjiang | 2018 |
| PRRSV/S78 | OM201177.1 | - | Heilongjiang | 2018 |
| AH-PRRS20178-1 | MW853923.1 | - | Jiangsu | 2017 |
| SH | OR102498.1 | - | Hubei | 2020 |
| WUH5 | KU523366.1 |  |  | 2015 |
| NADC30-IR 40.6% (103/254) | | | | |
| SDHY_DZ037 | OP168793.1 | NADC34/JXA1 | Shandong | 2020 |
| GXNN20210506 | OK486524.1 | NADC34/JXA1 | Guangxi | 2021 |
| GXFCG20210401 | OK486522.1 | NADC34/JXA1 | Guangxi | 2021 |
| SCABTC-202309 | OR766560.1 | JXA1 | Sichuan | 2023 |
| CH-WH-2019-1 | MK450333.1 | VR2332/JXA1 | Hubei | 2018 |
| PRRSV/H013 | OM201189.1 | VR2332/JXA1 | Heilongjiang | 2018 |
| SDQD-1604 | MH651742.1 | VR2332/JXA1 | Heilongjiang | 2016 |
| PRRSV/S136 | OM201179.1 | VR2332/JXA1 | Heilongjiang | 2019 |
| HNTZJ1715-2102 |  | JXA1 | Henan | 2021 |
| HM1805 | OQ924468.1 | JXA1 | Beijing | 2018 |
| FS-GD-02 | MT036897.1 | JXA1 | Guangdong | 2016 |
| PRRSV2/CN/F2/2019 | OL422836.1 | QYYZ/JXA1 | Fujian | 2019 |
| FJWQ16 | KX758249.1 | QYYZ/JXA1 | Fujian |  |
| PRRSV2/CN/N2/2021 | OL422841.1 | QYYZ/JXA1 | Fujian | 2021 |
| PRRSV2/CN/S5/2018 | OL422829.1 | QYYZ/JXA1 | Fujian | 2018 |
| PRRSV2/CN/X2998/2018 | MT409690.1 | QYYZ/JXA1 | Fujian | 2018 |
| FJDJQ-2018 | MT416547.1 | QYYZ/JXA1 | Fujian | 2020 |
| FJDJQ-2018 | MN862433.1 | QYYZ/JXA1 | Fujian | 2019 |
| PRRSV2/CN/N4/2019 | OL422823.1 | QYYZ/JXA1 | Fujian | 2019 |
| PRRSV2/CN/N42/2017 | OL422828.1 | QYYZ/JXA1 | Fujian | 2017 |
| PRRSV2/CN/Z8/2018 | OL422831.1 | QYYZ/JXA1 | Fujian | 2018 |
| PRRSV2/CN/N0/2021 | OL422842.1 | QYYZ/JXA1 | Fujian | 2021 |
| PRRSV2/CN/X4836/2018 | MT409689.1 | QYYZ/JXA1 | Fujian | 2018 |
| FJLIUY-2017 | MG011718.1 | QYYZ/VR2332/JXA1 | Fujian | 2017 |
| ZJqz21 | OK274266.1 | QYYZ/VR2332/JXA1 | Shanghai | 2021 |
| SD-1602 | MH651743.1 | JXA1 | Heilongjiang | 2016 |
| 15LN3 | KX815425.1 | JXA1 | Jiangsu | 2015 |
| SCcd17 | MG914067.1 | JXA1 | Sichuan | 2017 |
| NMGTZJ3157-2310 |  | JXA1 | Inner Mongolia | 2023 |
| HLJTZJ3143-2310 |  | NADC34/JXA1 | Heilongjiang | 2023 |
| CHbj2103 | OP734318.1 | QYYZ/JXA1 | Beijing | 2021 |
| CHbj2102 | OP734317.1 | QYYZ/JXA1 | Beijing | 2021 |
| CHbj2101 | OP734316.1 | QYYZ/JXA1 | Beijing | 2021 |
| SCN17 | MH078490.1 | VR2332/JXA1 | Sichuan | 2017 |
| SCTZJ3124-2309 |  | NADC34/JXA1 | Sichuan | 2023 |
| HLJTZJ2090-2107 | OL516355.1 | NADC34/JXA1 | Heilongjiang | 2021 |
| HBFL-1604 | MH651739.1 | JXA1 | Heilongjiang | 2016 |
| GX20210501 | OQ204111.1 | JXA1 | Guangxi | 2021 |
| GXHX20211106 | OQ459665.1 | JXA1 | Guangxi | 2021 |
| GXNN202004a | MW531679.1 | JXA1 | Guangxi | 2020 |
| JLTZJ2050-2107 | OL516359.1 | NADC34/JXA1 | Heilongjiang | 2021 |
| HLJTZJ3268-2312 |  | JXA1 | Heilongjiang | 2023 |
| JL580 | KR706343.1 | JXA1 | Jilin | 2013 |
| CY1-1604 | MH651736.1 | JXA1 | Heilongjiang | 2016 |
| SDbz16-2 | MH588710.1 | JXA1 | Zhejiang | 2016 |
| SXht2012 | OR518274.1 | JXA1 | Shanxi | 2020 |
| GXGG20210301 | OQ459663.1 | JXA1 | Guangxi | 2021 |
| HLJTZJ2007-2106 | OL516354.1 | NADC34/JXA1 | Heilongjiang | 2021 |
| HLJWK871-2308 |  | JXA1 | Heilongjiang | 2023 |
| LNTZJ3211-2311 |  | JXA1 | Liaoning | 2023 |
| HN-NY/2023 | OR575928.1 | JXA1 | Henan | 2023 |
| HENZMD-9 | KU950374.1 | JXA1 | Henan | 2015 |
| PRRSV/H60 | OM201191.1 | JXA1 | Heilongjiang | 2018 |
| SC-d | MF375261.1 | JXA1 | Heilongjiang | 2015 |
| HuBXW | OR066233.1 | JXA1 | Hubei | 2020 |
| PRRSV/S043 | OM201173.1 | JXA1 | Heilongjiang | 2018 |
| PRRSV/H029 | OM201190.1 | JXA1 | Heilongjiang | 2018 |
| HuN-XT-B | OP784963.1 | JXA1 | Hebei | 2021 |
| GD1909 | MT165636.1 | JXA1 | Fujian | 2019 |
| HNTZJ3182-2310 |  | JXA1 | Henan | 2023 |
| PRRSV2/CN/F5/2018 | OL416125.1 | JXA1 | Fujian | 2018 |
| PRRSV2/CN/G7/2018 | OL416126.1 | JXA1 | Fujian | 2018 |
| FJDJQ-2017 | MG011719.1 | QYYZ | Fujian | 2017 |
| PRRSV2/CN/G8/2018 | OQ357725.1 | QYYZ/JXA1 | Fujian | 2018 |
| PRRSV2/CN/G9/2018 | OL416127.1 | QYYZ/JXA1 | Fujian | 2018 |
| Fujian-2014-18 | MN046225.1 | VR2332/JXA1 | Fujian | 2014 |
| HNLCL15-1903 | ON462043.1 | QYYZ/JXA1 | Heilongjiang | 2019 |
| CH/SCNC-2/2020 | MZ747443.1 | JXA1 | Sichuan | 2020 |
| SCya18 | MK144543.1 | QYYZ/JXA1 | Sichuan | 2018 |
| CHsx1401 | KP861625.1 | VR2332 | Beijing | 2014 |
| PRRSV/LN86 | OM201195.1 | VR2332 | Heilongjiang | 2018 |
| LNCH-1604 | MH651741.1 | NADC34 | Heilongjiang | 2016 |
| CH/SCYB-2/2019 | MZ747448.1 | VR2332 | Sichuan | 2019 |
| BL2019 | OQ735301.1 | JXA1 | Guangdong | 2019 |
| CH/SCCD-2/2018 | MZ747437.1 | JXA1 | Sichuan | 2018 |
| HENAN-XINX | KF611905.1 | VR2332 | Henan | 2013 |
| HNJYH-1606 | MH651740.1 | JXA1 | Heilongjiang | 2016 |
| HNjz15 | KT945017.1 | NADC34 | Henan | 2015 |
| HLJTZJ3187-2310 |  | NADC34 | Heilongjiang | 2023 |
| HEB-108 | MN046224.1 | JXA1 | Heilongjiang | 2017 |
| HENJY-2 | KX900392.1 | JXA1 | Henan | 2015 |
| HNJYF-1606 | MH651738.1 | JXA1 | Heilongjiang | 2016 |
| WUH6 | KU523367.1 | JXA1 | Hubei | 2016 |
| GDHZ | MT036898.1 | JXA1 | Guangdong | 2017 |
| PRRSV2/CN/I9/2018 | OL416129.1 | QYYZ/JXA1 | Fujian | 2019 |
| qy 2104 | OR800930.1 | QYYZ | Guangdong | 2021 |
| qy 2105 | OR800925.1 | QYYZ | Guangdong | 2021 |
| GDhy-1809 | OM949992.1 | QYYZ | Guangdong | 2018 |
| PRRSV2/CN/X4839/2017 | MT409692.1 | JXA1 | Fujian | 2017 |
| FJ1402 | KX169191.1 | JXA1 | Jiangsu | 2014 |
| FJM4 | KY412888.1 | JXA1 | Fujian | 2014 |
| FJL15 | KY412887.1 | JXA1 | Fujian | 2014 |
| PRRSV2/CN/N3/2017 | OL422827.1 | JXA1 | Fujian | 2017 |
| PRRSV2/CN/X4833/2018 | MT409691.1 | JXA1 | Fujian | 2018 |
| PRRSV2/CN/F7/2017 | OL422830.1 | JXA1 | Fujian | 2017 |
| PRRSV2/CN/X9830/2018 | MT409688.1 | JXA1 | Fujian | 2018 |
| HBag-4 | OP131596.1 | JXA1 | Hebei | 2020 |
| BJ2021 | OK095299.1 | JXA1 | Beijing | 2021 |
| JSWA | KY373214.1 | VR2332 | Shandong | 2014 |
| CY2-1604 | MH651737.1 | VR2332 | Heilongjiang | 2016 |
| PRRSV/S145 | OM201180.1 | VR2332 | Heilongjiang | 2019 |
| PRRSV/S130 | OM201178.1 | VR2332 | Heilongjiang | 2019 |
| HLJTZJ3205-2310 |  | NADC34/JXA1 | Heilongjiang | 2023 |
