# Supplementary material for: Genomic characteristics and epidemic trends of NADC30-like PRRSV in China
Source: Porcine Health Manag. 2025 May 28;11:30. doi: 10.1186/s40813-025-00444-7 (PMC12121172; doi:10.1186/s40813-025-00444-7)
Supplement: Supplementary file 4 — Supplementary Material 4: Table S3. Information on recombination events of NADC30-like PRRSV detected by RPD4 software [file 40813_2025_444_MOESM4_ESM.docx]

**TABLE** **S3 Information on recombination events of NADC30-like PRRSV detected by RPD4 software.**

| Strains | Breakpoints | | Parental Sequence | | Detection Methods (p-Value) | | | | | | |
| --- | --- | --- | --- | --- | --- | --- | --- | --- | --- | --- | --- |
|  | Beginning | Ending | Minor | Major | RDP | GENECO | BootScan | MaxChi | Chimaera | SiScan | 3Seq |
| NADC30-R1 | | | | | | | | | | | |
| SD17-38 | 6229 | 6880 | JXA1 | NADC30 | 6.82×10^-27^ | 9.77×10^-13^ | 2.45×10^-23^ | 3.01×10^-10^ | 1.38×10^-11^ | 1.89×10^-8^ | 1.94×10^-14^ |
|  | 12305 | 12775 | VR2332 | NADC30 | 3.98×10^-24^ | 2.01×10^-10^ | 2.20×10^-23^ | 6.06×10^-10^ | 1.31×10^-11^ | 8.75×10^-6^ | 1.94×10^-14^ |
| HLJ-DZD1-1804 | 7384 | 7660 | VR2332 | NADC30 | 2.481×10^-6^ | 1.19×10^-4^ | 1.39×10^-06^ | 1.18×10^-03^ | 5.94×10^-4^ | - | - |
|  | 12320 | 12772 | VR2332 | NADC30 | 6.13×10^-29^ | 1.51×10^-15^ | 5.31×10^-26^ | 2.17×10^-13^ | 1.14×10^-15^ | 4.55×10^-11^ | 1.78×10^-15^ |
|  | 13177 | 14991 | JXA1 | NADC30 | 2.16×10^-109^ | 5.90×10^-102^ | 5.28×10^-110^ | 1.81×10^-30^ | 2.59×10^-23^ | 5.23×10^-37^ | 2.22×10^-15^ |
| SCABTC-202302 | 7374 | 7616 | VR2332 | NADC30 | 8.60×10^-5^ | - | 4.54×10^-5^ | 2.32×10^-2^ | 3.66×10^-3^ | - | 6.53×10^-5^ |
|  | 11688 | 14500 | JXA1 | NADC30 | 2.48×10^-150^ | 4.18×10^-143^ | 5.05×10^-152^ | 2.09×10^-40^ | 4.64×10^-40^ | 1.15×10^-51^ | 1.78×10^-15^ |
| 2014-81 | 7359 | 7623 | VR2332 | NADC30 | 6.16×10^-07^ | - | 3.64×10^-07^ | 1.99×10^-4^ | 7.81×10^-5^ | 1.79×10^-3^ | 1.61×10^-7^ |
|  | 9081 | 15013 | JXA1 | NADC30 | 8.68×10^-136^ | 3.17×10^-97^ | 3.61×10^-136^ | 4.74×10^-43^ | 6.91×10^-49^ | 5.61×10^-49^ | 1.78×10^-15^ |
| SD53-1603 | 7365 | 7629 | VR2332 | NADC30 | 3.84×10^-10^ | 1.99×10^-4^ | 3.21×10^-10^ | 1.20×10^-5^ | 5.64×10^-6^ | - | 2.14×10^-8^ |
|  | 12305 | 12776 | VR2332 | NADC30 | 5.00×10^-29^ | 4.81×10^-21^ | 7.40×10^-30^ | 2.71×10^-13^ | 2.13×10^-14^ | - | 5.55×10^-16^ |
| SD | 7365 | 7629 | VR2332 | NADC30 | 3.78×10^-10^ | 1.97×10^-4^ | 3.15×10^-10^ | 1.20×10^-5^ | 5.64×10^-6^ | - | 2.07×10^-8^ |
|  | 12305 | 12774 | VR2332 | NADC30 | 1.21×10^-27^ | 4.07×10^-19^ | 1.88×10^-28^ | 2.37×10^-13^ | 2.07×10^-14^ | - | 5.55×10^-16^ |
| HN-1 | 7354 | 7630 | VR2332 | NADC30 | 6.42×10^-09^ | 1.17×10^-3^ | 2.01×10^-10^ | 6.34×10^-6^ | 1.97×10^-6^ | - | 7.58×10^-9^ |
|  | 12306 | 12774 | VR2332 | NADC30 | 8.66×10^-27^ | 4.23×10^-17^ | 1.84×10^-27^ | 4.48×10^-13^ | 4.22×10^-14^ | - | 5.55×10^-16^ |
| QHD1 | 7365 | 7614 | VR2332 | NADC30 | 2.77×10^-8^ | 7.98×10^-6^ | 2.86×10^-9^ | 2.99×10^-4^ | 1.31×10^-6^ | - | 5.84×10^-8^ |
|  | 12312 | 12776 | VR2332 | NADC30 | 3.51×10^-26^ | 1.38×10^-11^ | 8.94×10^-26^ | 4.49×10^-12^ | 4.82×10^-13^ | - | 5.55×10^-16^ |
| PRRSV-HB-16-China-2019 | 7137 | 7386 | VR2332 | NADC30 | 2.76×10^-7^ | - | 4.28×10^-7^ | 2.57×10^-3^ | 2.15×10^-5^ | - | 1.83×10^-6^ |
|  | 12077 | 12548 | VR2332 | NADC30 | 3.93×10^-27^ | 6.20×10^-16^ | 8.90×10^-29^ | 2.26×10^-13^ | 3.35×10^-14^ | - | 5.55×10^-16^ |
| HLJ-80 | 7365 | 7629 | VR2332 | NADC30 | 1.50×10^-7^ | - | 9.06×10^-8^ | 7.11×10^-5^ | 2.87×10^-5^ | - | 3.35×10^-8^ |
|  | 12294 | 12776 | VR2332 | NADC30 | 4.21×10^-29^ | 9.80×10^-19^ | 1.03×10^-30^ | 1.38×10^-13^ | 2.16×10^-14^ | - | 5.55×10^-16^ |
| SWU/MY5/2018 | 7327 | 7622 | VR2332 | NADC30 | 1.54×10^-3^ | - | 1.84×10^-3^ | 8.40×10^-3^ | 3.22×10^-3^ | - | 2.36×10^-4^ |
|  | 12267 | 12736 | VR2332 | NADC30 | 5.15×10^-28^ | 6.13×10^-14^ | 4.39×10^-25^ | 3.62×10^-12^ | 1.46×10^-13^ | - | 5.55×10^-16^ |
| SWU/MY6/2018 | 7365 | 7629 | VR2332 | NADC30 | 3.29×10^-5^ | - | 5.03×10^-4^ | 3.87×10^-3^ | 3.51×10^-4^ | - | 2.31×10^-5^ |
|  | 12305 | 12774 | VR2332 | NADC30 | 3.63×10^-28^ | 4.60×10^-14^ | 3.38×10^-25^ | 3.63×10^-12^ | 1.50×10^-13^ | - | 5.55×10^-16^ |
| SWU/MS2/2018 | 7377 | 7626 | VR2332 | NADC30 | 4.20×10^-9^ | 3.96×10^-02^ | 3.79×10^-9^ | 2.39×10^-4^ | 8.00×10^-5^ | - | 1.17×10^-7^ |
|  | 12317 | 12773 | VR2332 | NADC30 | 1.53×10^-26^ | 1.31×10^-10^ | 1.03×10^-25^ | 1.10×10^-11^ | 2.48×10^-13^ | - | 5.55×10^-16^ |
| SWU/MS3/2018 | 7371 | 7626 | VR2332 | NADC30 | 9.54×10^-10^ | 1.11×10^-3^ | 8.13×10^-10^ | 6.31×10^-4^ | 1.40×10^-5^ | - | 1.10×10^-8^ |
|  | 12317 | 12773 | VR2332 | NADC30 | 1.17×10^-27^ | 2.95×10^-14^ | 2.60×10^-27^ | 6.81×10^-11^ | 1.51×10^-13^ | - | 5.55×10^-16^ |
| SWU/YB1/2018 | 7377 | 7629 | VR2332 | NADC30 | 1.57×10^-8^ | 3.84×10^-2^ | 3.44×10^-09^ | 1.70×10^-5^ | 7.96×10^-5^ | - | 1.16×10^-7^ |
|  | 12321 | 12776 | VR2332 | NADC30 | 1.23×10^-27^ | 1.82×10^-14^ | 6.06×10^-27^ | 7.71×10^-12^ | 2.47×10^-13^ | - | 5.55×10^-16^ |
| SWU/CD1/2018 | 7332 | 7584 | VR2332 | NADC30 | 1.49×10^-07^ | - | 4.59×10^-8^ | 1.69×10^-5^ | 7.94×10^-5^ | - | 2.82×10^-7^ |
|  | 12275 | 12731 | VR2332 | NADC30 | 2.29×10^-25^ | 2.41×10^-11^ | 3.82×10^-25^ | 1.49×10^-10^ | 6.56×10^-13^ | - | 5.55×10^-16^ |
| SWU/YB2/2018 | 7342 | 7594 | VR2332 | NADC30 | 1.34×10^-8^ | - | 3.48×10^-8^ | 1.46×10^-4^ | 2.22×10^-4^ | - | 3.00×10^-7^ |
|  | 12285 | 12741 | VR2332 | NADC30 | 1.54×10^-26^ | 5.88×10^-16^ | 2.61×10^-25^ | 1.04×10^-11^ | 3.19×10^-13^ | - | 5.55×10^-16^ |
| GXNN1839 | 7374 | 7629 | VR2332 | NADC30 | 2.93×10^-8^ | - | 2.68×10^-8^ | 3.58×10^-3^ | 7.94×10^-5^ | - | 1.44×10^-7^ |
|  | 12320 | 12776 | VR2332 | NADC30 | 4.85×10^-25^ | 2.19×10^-14^ | 4.68×10^-23^ | 5.72×10^-12^ | 8.83×10^-13^ | - | 5.55×10^-16^ |
| PRRSV/S001 | 7365 | 7617 | VR2332 | NADC30 | 1.05×10^-7^ | - | 5.57×10^-8^ | 1.70×10^-5^ | 6.33×10^-5^ | - | 1.45×10^-8^ |
|  | 12308 | 12764 | VR2332 | NADC30 | 2.46×10^-23^ | 1.12×10^-11^ | 6.96×10^-24^ | 1.14×10^-9^ | 4.75×10^-14^ | - | 5.55×10^-16^ |
| TZJ3184 | 7657 | 8053 | VR2332 | NADC30 | 4.59×10^-3^ | - | 3.73×10^-4^ | 3.16×10-^2^ | 3.07×10^-2^ | 4.53×10^-10^ | - |
|  | 12816 | 13271 | VR2332 | NADC30 | 7.51×10^-23^ | 1.51×10^-10^ | 2.96×10^-23^ | 2.22×10^-10^ | 1.08×10^-11^ | 7.89×10^-9^ | 1.55×10^-14^ |
| PRRSV/XJ-1 | 7283 | 7568 | VR2332 | NADC30 | 7.78×10^-07^ | - | 4.01×10^-7^ | 2.74×10^-6^ | 7.67×10^-6^ | - | 1.82×10^-7^ |
|  | 10610 | 11040 | JXA1 | NADC30 | 4.31×10^-18^ | 9.44×10^-11^ | 9.95×10^-19^ | 6.80×10^-9^ | 2.56×10^-10^ | 1.29×10^-7^ | 1.17×10^-2^ |
|  | 12217 | 12673 | VR2332 | NADC30 | 1.40×10^-25^ | 1.08×10^-10^ | 2.23×10^-24^ | 7.21×10^-9^ | 6.29×10^-11^ | 2.20×10^-12^ | 2.22×10^-15^ |
| 2023GD-4 | 7385 | 7671 | VR2332 | NADC30 | 1.13×10^-2^ | - | 1.06×10^-2^ | 3.13×10^-7^ | 6.24×10^-5^ | - | 5.57×10^-6^ |
|  | 10749 | 11129 | JXA1 | NADC30 | 3.00×10^-15^ | 3.78×10^-5^ | 7.65×10^-16^ | 3.13×10^-8^ | 3.18×10^-8^ | 5.60×10^-7^ | 8.39×10^-14^ |
|  | 12346 | 12776 | VR2332 | NADC30 | 1.24×10^-22^ | 2.50×10^-10^ | 8.10×10^-22^ | 2.06×10^-9^ | 2.43×10^-5^ | 1.35×10^-9^ | 1.78×10^-15^ |
| TZJ3059 | 7328 | 7613 | VR2332 | NADC30 | 1.03×10^-5^ | - | 1.27×10^-5^ | 1.37×10^-3^ | 1.26×10^-4^ | - | 6.62×10^-5^ |
|  | 12219 | 12689 | VR2332 | NADC30 | 1.03×10^-19^ | 1.40×10^-3^ | 3.85×10^-17^ | 7.44×10^-10^ | 5.09×10^-11^ | - | 6.66×10^-16^ |
| SCABTC-202308 | 7909 | 8162 | VR2332 | NADC30 | 1.33×10^-4^ | 8.68×10^-4^ | 2.43×10^-4^ | 3.03×10^-6^ | 2.78×10^-3^ | 1.08×10^-11^ | - |
|  | 12270 | 12790 | VR2332 | NADC30 | 1.41×10^-22^ | 3.14×10^-6^ | 5.69×10^-22^ | 7.50×10^-10^ | 3.85×10^-11^ | 5.80×10^-11^ | 1.55×10^-14^ |
| SDQZ-1609 | 7377 | 7677 | VR2332 | NADC30 | 5.38×10^-9^ | 2.47×10^-4^ | 2.91×10^-9^ | 9.21×10^-4^ | 3.57×10^-4^ | - | 3.38×10^-8^ |
| SD99-1606 | 7377 | 7650 | VR2332 | NADC30 | 3.60×10^-10^ | 1.15×10^-6^ | 1.96×10^-10^ | 4.69×10^-5^ | 2.39×10^-5^ | - | 2.37×10^-9^ |
| SDZC-1609 | 7375 | 7651 | VR2332 | NADC30 | 2.30×10^-8^ | 7.92×10^-4^ | 1.26×10^-8^ | 3.70×10^-6^ | 2.18×10^-5^ | 6.95×10^-6^ | 1.04×10^-7^ |
|  | 13451 | 15022 | JXA1 | NADC30 | 1.17×10^-114^ | 1.53×10^-111^ | 1.43×10^-116^ | 1.43×10^-22^ | 5.60×10^-13^ | 1.92×10^-36^ | 2.22×10^-15^ |
| SD1805 | 7378 | 7653 | VR2332 | NADC30 | 1.57×10^-9^ | 4.98×10^-5^ | 9.24×10^-10^ | 2.53×10^-6^ | 2.99×10^-5^ | - | 6.60×10^-7^ |
| GDsf1804 | 7384 | 7660 | VR2332 | NADC30 | 2.48×10^-6^ | 1.19×10^-4^ | 1.39×10^-6^ | 1.18×10^-3^ | 5.94×10^-4^ | - | - |
|  | 12320 | 12782 | VR2332 | NADC30 | 1.21×10^-25^ | 1.71×10^-14^ | 8.03×10^-23^ | 1.12×10^-13^ | 8.96×10^-5^ | - | - |
| GDsf1806 | 7377 | 7671 | VR2332 | NADC30 | 1.36×10^-6^ | - | 1.74×10^-7^ | 3.06×10^-5^ | 3.74×10^-6^ | - | 1.24×10^-7^ |
|  | 12435 | 12751 | VR2332 | NADC30 | 5.25×10^-23^ | 4.94×10^-11^ | 1.28×10^-20^ | 3.54×10^-9^ | 7.98×10^-10^ | - | 5.55×10^-16^ |
| GDsf1808 | 7377 | 7671 | VR2332 | NADC30 | 2.44×10^-6^ | - | 3.64×10^-7^ | 2.95×10^-5^ | 3.74×10^-6^ | - | 1.30×10^-7^ |
|  | 12435 | 12751 | VR2332 | NADC30 | 5.40×10^-23^ | 5.12×10^-11^ | 1.26×10^-20^ | 3.55×10^-9^ | 7.98×10^-10^ | - | 5.55×10^-16^ |
| GDsf1807 | 7384 | 7678 | VR2332 | NADC30 | 1.30×10^-6^ | - | 1.63×10^-7^ | 3.06×10^-5^ | 3.73×10^-6^ | - | 1.22×10^-7^ |
|  | 12442 | 12759 | VR2332 | NADC30 | 4.72×10^-23^ | 5.01×10^-11^ | 1.67×10^-20^ | 3.55×10^-9^ | 7.97×10^-10^ | - | 5.55×10^-16^ |
| GDsf1809 | 7377 | 7671 | VR2332 | NADC30 | 6.91×10^-6^ | - | 1.19×10^-6^ | 1.45×10^-4^ | 1.73×10^-5^ | - | 1.20×10^-6^ |
|  | 12435 | 12751 | VR2332 | NADC30 | 4.01×10^-22^ | 3.84×10^-9^ | 7.03×10^-22^ | 7.36×10^-9^ | 1.86×10^-9^ | - | 5.55×10^-6^ |
| GDsc1808 | 7377 | 7671 | VR2332 | NADC30 | 1.17×10^-7^ | - | 9.91×10^-9^ | 7.62×10^-5^ | 1.90×10^-5^ | - | 9.08×10^-8^ |
|  | 12435 | 12751 | VR2332 | NADC30 | 6.37×10^-23^ | 2.02×10^-10^ | 1.28×10^-20^ | 4.13×10^-9^ | 7.97×10^-10^ | - | 5.55×10^-16^ |
| GDsc1809 | 7377 | 7671 | VR2332 | NADC30 | 1.21×10^-7^ | - | 1.03×10^-8^ | 6.14×10^-5^ | 3.74×10^-6^ | - | 9.35×10^-8^ |
|  | 12435 | 12751 | VR2332 | NADC30 | 1.92×10^-22^ | 2.07×10^-10^ | 3.11×10^-20^ | 2.02×10^-8^ | 7.98×10^-10^ | - | 5.55×10^-16^ |
| NADC30-R2 | | | | | | | | | | | |
| WK961 | 5158 | 8684 | JXA1 | NADC30 | 3.07×10^-13^ | - | 1.13×10^-13^ | 5.74×10^-15^ | 1.47×10^-22^ | 7.60×10^-9^ | 1.33×10^-15^ |
|  | 11719 | 13948 | IA/2014/NADC34 | NADC30 | 1.52×10^-8^ | - | 1.30×10^-11^ | 1.45×10^-11^ | 2.73×10^-12^ | 1.33×10^-7^ | 1.30×10^-10^ |
| TZJ3132 | 4834 | 7208 | JXA1 | NADC30 | 3.32×10^-71^ | 3.89×10^-25^ | 1.64×10^-75^ | 9.54×10^-29^ | 2.87×10^-15^ | 9.90×10^-32^ | 1.15×10^-14^ |
|  | 11688 | 14134 | IA/2014/NADC34 | NADC30 | 8.09×10^-41^ | 4.87×10^-21^ | 1.25×10^-42^ | 4.16×10^-18^ | 3.69×10^-10^ | 6.31×10^-24^ | 1.33×10^-15^ |
| TZJ3116 | 1463 | 2001 | JXA1 | NADC30 | 3.68×10^-36^ | 8.22×10^-10^ | 7.25×10^-36^ | 1.404×10^-12^ | 3.97×10^-19^ | - | 1.09×10^-11^ |
|  | 4910 | 7679 | JXA1 | NADC30 | 6.38×10^-70^ | 6.69×10^-27^ | 1.34×10^-69^ | 2.02×10^-31^ | 6.86×10^-36^ | - | 4.44×10^-16^ |
|  | 11670 | 14051 | IA/2014/NADC34 | NADC30 | 4.44×10^-54^ | 2.14×10^-27^ | 3.82×10^-54^ | 6.34×10^-22^ | 2.10×10^-17^ | - | 3.33×10^-16^ |
| TZJ3115 | 660 | 2020 | JXA1 | NADC30 | 8.13×10^-100^ | 8.27×10^-101^ | 1.41×10^-96^ | 5.80×10^-37^ | 1.56×10^-2^ | - | - |
|  | 4884 | 7668 | JXA1 | NADC30 | 2.97×10^-46^ | 1.00×10^-14^ | 4.23×10^-46^ | 2.84×10^-20^ | 7.34×10^-22^ | - | 4.44×10^-16^ |
|  | 12305 | 14051 | IA/2014/NADC34 | NADC30 | 4.40×10^-27^ | 1.51×10^-13^ | 4.45×10^-26^ | 5.51×10^-10^ | 9.34×10^-8^ | 9.70×10^-13^ | 1.33×10^-15^ |
| TZJ3290 | 1 | 541 | JXA1 | NADC30 | 1.51×10^-5^ | - | 5.40×10^-5^ | 7.44×10^-5^ | 1.18×10^-3^ | 1.16×10^-03^ | - |
|  | 5189 | 7967 | JXA1 | NADC30 | 5.07×10^-73^ | 4.11×10^-41^ | 1.26×10^-71^ | 3.74×10^-30^ | 1.15×10^-9^ | 1.16×10^-35^ | 1.33×10^-15^ |
|  | 11708 | 14120 | IA/2014/NADC34 | NADC30 | 1.90×10^-36^ | 7.12×10^-20^ | 1.69×10^-34^ | 7.64×10^-18^ | 1.44×10^-19^ | 2.48×10^-21^ | 8.88×10^-16^ |
| HLJTZJ2165-2108 | 5219 | 7967 | JXA1 | NADC30 | 2.82×10^-70^ | 1.31×10^-37^ | 1.32×10^-72^ | 9.56×10^-30^ | 1.71×10^-15^ | 1.30×10^-33^ | 1.33×10^-15^ |
|  | 11737 | 14017 | IA/2014/NADC34 | NADC30 | 9.27×10^-48^ | 1.30×10^-42^ | 2.03×10^-46^ | 3.02×10^-20^ | 1.26×10^-21^ | 1.58×10^-25^ | 1.33×10^-15^ |
| PRRSV-China/SCcd2020/2020 | 3583 | 4086 | JXA1 | NADC30 | 1.54×10^-32^ | 2.14×10^-24^ | 1.10×10^-32^ | 5.10×10^-13^ | 8.30×10^-14^ | - | 4.44×10^-16^ |
|  | 4923 | 7618 | JXA1 | NADC30 | 4.54×10^-54^ | 1.64×10^-40^ | 3.23×10^-54^ | 3.22×10^-20^ | 3.58×10^-23^ | - | 4.44×10^-16^ |
|  | 11675 | 14163 | IA/2014/NADC34 | NADC30 | 4.27×10^-57^ | 8.06×10^-38^ | 7.96×10^-58^ | 8.23×10^-24^ | 2.62×10^-17^ | - | 3.33×10^-16^ |
| WK730 | 5095 | 7681 | JXA1 | NADC30 | 6.51×10^-48^ | 2.60×10^-31^ | 2.11×10^-46^ | 4.70×10^-17^ | 2.85×10^-20^ | 4.994×10^-18^ | 1.33×10^-15^ |
|  | 11675 | 14056 | IA/2014/NADC34 | NADC30 | 1.24×10^-49^ | 1.51×10^-21^ | 6.22×10^-51^ | 1.95×10^-21^ | 4.26×10^-16^ | - | 3.33×10^-16^ |
| WK621 | 4923 | 7678 | JXA1 | NADC30 | 1.294×10^-45^ | 3.698×10^-27^ | 6.91×10^-45^ | 3.20×10^-18^ | 1.23×10^-12^ | 2.274×10^-18^ | 1.77×10^-15^ |
|  | 11982 | 14059 | IA/2014/NADC34 | NADC30 | 1.22×10^-46^ | 2.82×10^-23^ | 1.68×10^-48^ | 4.45×10^-21^ | 2.42×10^-17^ | - | 3.33×10^-16^ |
| JS2020 | 4921 | 7664 | JXA1 | NADC30 | 1.00×10^-51^ | - | 4.59×10^-50^ | 6.77×10^-22^ | 8.94×10^-15^ | - | 1.11×10^-16^ |
|  | 11670 | 14051 | IA/2014/NADC34 | NADC30 | 6.43×10^-57^ | 2.04×10^-36^ | 4.45×10^-58^ | 1.26×10^-05^ | 3.10×10^-2^ | - | 3.33×10^-16^ |
| TZJ3291 | 5210 | 7719 | JXA1 | NADC30 | 5.43×10^-43^ | 1.92×10^-16^ | 4.29×10^-44^ | 6.26×10^-17^ | 8.95×10^-18^ | 6.04×10^-19^ | 2.62×10^-13^ |
|  | 11771 | 13945 | IA/2014/NADC34 | NADC30 | 6.43×10^-38^ | 1.62×10^-19^ | 1.08×10^-40^ | 6.51×10^-3^ | 3.75×10^-13^ | 1.36×10^-23^ | 8.88×10^-16^ |
| CH-HNPY-01/2022 | 4919 | 7665 | JXA1 | NADC30 | 4.05×10^-47^ | 2.68×10^-30^ | 5.47×10^-47^ | 2.35×10^-20^ | 6.86×10^-23^ | - | 1.11×10^-16^ |
|  | 11671 | 14052 | IA/2014/NADC34 | NADC30 | 1.31×10^-52^ | 1.60×10^-32^ | 6.00×10^-54^ | 1.83×10^-21^ | 1.40×10^-3^ | - | 3.33×10^-16^ |
| GD-F1 | 4887 | 7677 | JXA1 | NADC30 | 2.01×10^-82^ | 1.62×10^-27^ | 6.39×10^-84^ | 8.02×10^-34^ | 9.93×10^-14^ | 2.26×10^-34^ | 1.77×10^-15^ |
|  | 11736 | 14055 | IA/2014/NADC34 | NADC30 | 8.57×10^-56^ | 4.04×10^-36^ | 1.46×10^-57^ | 9.35×10^-21^ | 3.95×10^-22^ | - | 4.440×10^-16^ |
| PRRSV2/CN/FJGD01/2021 | 4922 | 7668 | JXA1 | NADC30 | 5.35×10^-50^ | 1.13×10^-29^ | 1.34×10^-46^ | 4.83×10^-21^ | 2.68×10^-13^ | - | 1.110×10^-16^ |
|  | 11674 | 14055 | IA/2014/NADC34 | NADC30 | 1.14×10^-49^ | 5.08×10^-28^ | 1.13×10^-50^ | 3.99×10^-20^ | 7.77×10^-4^ | - | 3.33×10^-16^ |
| TZJ3005 | 4923 | 7658 | JXA1 | NADC30 | 2.47×10^-43^ | - | 2.81×10^-43^ | 1.24×10^-17^ | 8.18×10^-21^ | 3.53×10^-18^ | 1.77×10^-15^ |
|  | 11737 | 14039 | IA/2014/NADC34 | NADC30 | 8.22×10^-52^ | 1.50×10^-30^ | 1.54×10^-52^ | 9.69×10^-23^ | 4.44×10^-24^ | - | 3.33×10^-16^ |
| YC-2020 | 4925 | 7668 | JXA1 | NADC30 | 2.45×10^-96^ | 1.33×10^-36^ | 3.05×10^-95^ | 1.50×10^-33^ | 1.04×10^-21^ | - | 4.44×10^-16^ |
|  | 11737 | 14056 | IA/2014/NADC34 | NADC30 | 2.28×10^-65^ | 1.50×10^-49^ | 3.21×10^-67^ | 2.97×10^-23^ | 1.01×10^-24^ | - | 4.44×10^-16^ |
| TZJ3243 | 4907 | 8388 | JXA1 | NADC30 | 9.70×10^-94^ | 4.06×10^-63^ | 2.94×10^-94^ | 1.18×10^-34^ | 5.70×10^-38^ | 6.12×10^-39^ | 1.33×10^-15^ |
|  | 11810 | 14145 | IA/2014/NADC34 | NADC30 | 4.09×10^-46^ | 7.74×10^-34^ | 6.66×10^-45^ | 5.84×10^-20^ | 8.92×10^-21^ | 6.71×10^-24^ | 8.88×10^-16^ |
| WK960 | 4907 | 8388 | JXA1 | NADC30 | 1.09×10^-93^ | 4.25×10^-63^ | 3.29×10^-94^ | 1.18×10^-34^ | 5.70×10^-38^ | 6.12×10^-39^ | 1.33×10^-15^ |
|  | 11810 | 14136 | IA/2014/NADC34 | NADC30 | 4.09×10^-46^ | 7.74×10^-34^ | 6.66×10^-45^ | 5.84×10^-20^ | 8.92×10^-21^ | 6.71×10^-24^ | 8.88×10^-16^ |
| hy 2203 | 5209 | 7969 | JXA1 | NADC30 | 1.95×10^-73^ | 6.27×10^-29^ | 5.81×10^-74^ | 2.25×10^-29^ | 1.70×10^-33^ | 3.31×10^-35^ | 1.78×10^-15^ |
|  | 11658 | 14124 | IA/2014/NADC34 | NADC30 | 4.28×10^-45^ | 1.40×10^-40^ | 3.29×10^-46^ | 7.72×10^-20^ | 4.38×10^-21^ | 8.97×10^-26^ | 1.33×10^-15^ |
| GD-H1 | 4922 | 7668 | JXA1 | NADC30 | 3.969×10^-47^ | 1.05×10^-24^ | 6.57×10^-44^ | 3.32×10^-20^ | 5.20×10^-13^ | - | 1.11×10^-16^ |
|  | 11674 | 14114 | IA/2014/NADC34 | NADC30 | 1.662×10^-47^ | 7.42×10^-27^ | 1.23×10^-49^ | 9.05×10^-4^ | 2.35×10^-4^ | - | 3.33×10^-16^ |
| SDlz20-04 | 4920 | 7677 | JXA1 | NADC30 | 1.46×10^-97^ | 1.63×10^-40^ | 3.89×10^-96^ | 2.13×10^-24^ | 2.62×10^-32^ | - | 4.44×10^-16^ |
|  | 11674 | 14217 | IA/2014/NADC34 | NADC30 | 6.96×10^-61^ | 4.33×10^-41^ | 6.87×10^-61^ | 2.25×10^-23^ | 2.46×10^-16^ | - | 4.44×10^-16^ |
| BJ20-06 | 4920 | 7677 | JXA1 | NADC30 | 1.01×10^-96^ | 1.43×10^-41^ | 2.83×10^-97^ | 6.61×10^-32^ | 6.92×10^-37^ | 2.04×10^-33^ | 1.77×10^-15^ |
|  | 11685 | 14217 | IA/2014/NADC34 | NADC30 | 1.64×10^-61^ | 5.40×10^-42^ | 1.20×10^-61^ | 1.00×10^-5^ | 1.12×10^-18^ | - | 4.44×10^-16^ |
| TZJ3134 | 5209 | 7941 | JXA1 | NADC30 | 1.06×10^-69^ | 1.42×10^-15^ | 2.76×10^-71^ | 1.50×10^-26^ | 2.04×10^-30^ | 3.45×10^-30^ | 1.78×10^-15^ |
|  | 11810 | 14249 | IA/2014/NADC34 | NADC30 | 3.09×10^-37^ | 5.23×10^-23^ | 7.14×10^-38^ | 3.94×10^-17^ | 1.34×10^-10^ | 3.90×10^-21^ | 1.33×10^-15^ |
| GXQZ20210403 | 651 | 1331 | JXA1 | NADC30 | 1.03×10^-49^ | 1.71×10^-38^ | 1.22×10^-50^ | 2.95×10^-21^ | 3.37×10^-22^ | - | - |
|  | 4905 | 7667 | JXA1 | NADC30 | 2.21×10^-43^ | 2.82×10^-36^ | 1.81×10^-44^ | 6.04×10^-32^ | 2.70×10^-8^ | - | 4.44×10^-16^ |
|  | 11704 | 14162 | IA/2014/NADC34 | NADC30 | 8.12×10^-63^ | 7.63×10^-50^ | 1.47×10^-57^ | 7.81×10^-22^ | 3.73×10^-25^ | - | 1.11×10^-16^ |
| TZJ3230 | 5195 | 7939 | JXA1 | NADC30 | 2.06×10^-39^ | 4.38×10^-5^ | 1.38×10^-39^ | 2.53×10^-24^ | 2.56×10^-25^ | 8.57×10^-33^ | 4.44×10^-16^ |
|  | 11673 | 14055 | IA/2014/NADC34 | NADC30 | 1.57×10^-59^ | - | 4.02×10^-58^ | 3.20×10^-34^ | 2.66×10^-15^ | 4.59×10^-37^ | 1.78×10^-15^ |
| TZJ2756 | 271 | 1968 | JXA1 | NADC30 | 6.32×10^-72^ | 1.72×10^-57^ | 1.25×10^-73^ | 7.69×10^-30^ | 3.19×10^-36^ | - | 4.40×10^-8^ |
|  | 5066 | 7694 | JXA1 | NADC30 | 1.49×10^-37^ | 1.08×10^-21^ | 5.96×10^-36^ | 2.85×10^-15^ | 4.77×10^-6^ | - | 3.33×10^-16^ |
|  | 11672 | 14215 | IA/2014/NADC34 | NADC30 | 3.99×10^-53^ | 8.19×10^-33^ | 5.18×10^-55^ | 6.49×10^-22^ | 1.918×10^-18^ | - | 3.33×10^-16^ |
| TZJ2451 | 141 | 2097 | JXA1 | NADC30 | 9.62×10^-78^ | 1.23×10^-67^ | 2.03×10^-77^ | 5.58×10^-34^ | 1.77×10^-36^ | 1.31×10^-34^ | 1.33×10^-15^ |
|  | 4905 | 7664 | JXA1 | NADC30 | 1.48×10^-62^ | 2.96×10^-23^ | 2.41×10^-64^ | 2.59×10^-24^ | 2.79×10^-16^ | 2.94×10^-37^ | 6.21×10^-15^ |
|  | 11743 | 14285 | IA/2014/NADC34 | NADC30 | 3.89×10^-22^ | 3.97×10^-13^ | 4.31×10^-22^ | 2.66×10^-5^ | 1.75×10^-2^ | 9.37×10^-35^ | - |
| NADC30-R3 | | | | | | | | | | | |
| GDsf1711 | 6453 | 6970 | JXA1 | NADC30 | 3.93×10^-25^ | 3.48×10^-9^ | 1.13×10^-23^ | 1.06×10^-10^ | 1.61×10^-12^ | - | 5.55×10^-16^ |
|  | 7401 | 8247 | JXA1 | NADC30 | 3.59×10^-32^ | - | 9.05×10^-33^ | 7.05×10^-15^ | 2.96×10^-15^ | - | 1.32×10^-9^ |
| GDsf1710 | 6453 | 6970 | JXA1 | NADC30 | 7.50×10^-26^ | 1.14×10^-10^ | 2.54×10^-24^ | 4.32×10^-11^ | 7.32×10^-13^ | - | 5.55×10^-16^ |
|  | 7401 | 8247 | JXA1 | NADC30 | 6.52×10^-33^ | - | 1.76×10^-33^ | 4.36×10^-15^ | 1.61×10^-15^ | - | 5.55×10^-16^ |
| GDsf1707 | 6453 | 6970 | JXA1 | NADC30 | 2.57×10^-25^ | 1.67×10^-10^ | 8.15×10^-24^ | 4.29×10^-11^ | 7.31×10^-13^ | - | 5.55×10^-16^ |
|  | 7401 | 8247 | JXA1 | NADC30 | 9.20×10^-34^ | 1.46×10^-9^ | 2.42×10^-34^ | 1.70×10^-15^ | 4.52×10^-16^ | - | 5.55×10^-16^ |
| GDsf1802 | 1170 | 1236 | JXA1 | NADC30 | 1.23×10^-6^ | 3.14×10^-3^ | - | 1.10×10^-3^ | 8.89×10^-4^ | - | 1.49×10^-5^ |
|  | 6452 | 6970 | JXA1 | NADC30 | 2.64×10^-22^ | 5.77×10^-5^ | 4.08×10^-21^ | 4.22×10^-10^ | 7.55×10^-12^ | - | 5.55×10^-16^ |
|  | 7400 | 8247 | JXA1 | NADC30 | 1.14×10^-31^ | 2.31×10^-6^ | 5.30×10^-32^ | 3.47×10^-15^ | 2.47×10^-15^ | - | 5.55×10^-16^ |
| GDYJ1224 | 463 | 1065 | JXA1 | NADC30 | 4.67×10^-28^ | 2.89×10^-24^ | 1.94×10^-28^ | 5.93×10^-16^ | 1.05×10^-12^ | - | - |
|  | 6015 | 6943 | JXA1 | NADC30 | 5.08×10^-25^ | - | 2.66×10^-24^ | 1.87×10^-12^ | 7.75×10^-16^ | - | 4.44×10^-16^ |
|  | 7373 | 8220 | JXA1 | NADC30 | 4.20×10^-22^ | - | 3.04×10^-22^ | 1.59×10^-14^ | 2.03×10^-15^ | - | 4.44×10^-16^ |
|  | 12551 | 12757 | JXA1 | NADC30 | 6.58×10^-18^ | 5.93×10^-13^ | 6.31×10^-14^ | 1.87×10^-6^ | 2.31×10^-3^ | - | 1.99×10^-3^ |
|  | 12875 | 13095 | JXA1 | NADC30 | 5.09×10^-14^ | 9.27×10^-11^ | 5.57×10^-13^ | 1.50×10^-2^ | 2.60×10^-6^ | - | 2.66×10^-11^ |
|  | 463 | 1065 | JXA1 | NADC30 | 4.67×10^-28^ | 2.89×10^-24^ | 1.94×10^-28^ | 5.93×10^-16^ | 1.05×10^-12^ | - | - |
| PRRSV2/CN/X4831/2018 | 6364 | 6988 | JXA1 | NADC30 | 7.39×10^-21^ | - | 7.89×10^-24^ | 9.07×10^-9^ | 9.59×10^-10^ | - | 6.66×10^-16^ |
|  | 7278 | 7761 | JXA1 | NADC30 | 1.09×10^-13^ | - | 7.69×10^-14^ | 1.79×10^-8^ | 1.55×10^-8^ | - | 3.33×10^-15^ |
| PRRSV2/CN/F0/2018 | 6364 | 6988 | JXA1 | NADC30 | 9.91×10^-21^ | - | 1.10×10^-23^ | 9.07×10^-9^ | 9.59×10^-10^ | - | 6.66×10^-16^ |
|  | 7278 | 7761 | JXA1 | NADC30 | 1.36×10^-13^ | - | 9.53×10^-14^ | 1.79×10^-8^ | 1.55×10^-8^ | - | 3.89×10^-15^ |
| PRRSV2/CN/Z0/2021 | 6364 | 6988 | JXA1 | NADC30 | 3.22×10^-16^ | - | 7.00×10^-17^ | 2.25×10^-6^ | 6.91×10^-8^ | - | 6.66×10^-16^ |
|  | 7157 | 7761 | JXA1 | NADC30 | 2.46×10^-11^ | - | 1.85×10^-11^ | 3.45×10^-8^ | 2.71×10^-8^ | - | 6.52×10^-11^ |
| PRRSV2/CN/F8/2020 | 6450 | 6536 | JXA1 | NADC30 | 9.05×10^-3^ | 6.95×10^-5^ | 9.56×10^-4^ | 1.65×10^-2^ | - | - | - |
|  | 6840 | 6988 | JXA1 | NADC30 | 6.02×10^-5^ | - | - | 6.20×10^-3^ | 3.10×10^-2^ | - | 4.49×10^-4^ |
|  | 7278 | 7761 | JXA1 | NADC30 | 7.97×10^-12^ | - | 4.79×10^-11^ | 1.28×10^-8^ | 1.61×10^-8^ | - | 3.44×10^-14^ |
| PRRSV2/CN/J2/2019 | 130 | 810 | JXA1 | NADC30 | 6.27×10^-14^ | - | 2.17×10^-12^ | 1.42×10^-9^ | 2.10×10^-10^ | - | 9.39×10^-13^ |
|  | 1638 | 1920 | JXA1 | NADC30 | 2.52×10^-14^ | 4.01×10^-18^ | 2.71×10^-18^ | 1.12×10^-9^ | 3.22×10^-10^ | - | - |
|  | 6472 | 6988 | JXA1 | NADC30 | 3.43×10^-15^ | 3.34×10^-8^ | 1.31×10^-15^ | 1.87×10^-6^ | 7.96×10^-8^ | - | 5.55×10^-16^ |
|  | 7474 | 8016 | JXA1 | NADC30 | 2.40×10^-14^ | - | 1.12×10^-14^ | 3.19×10^-6^ | 2.38×10^-8^ | - | 7.96×10^-14^ |
| PRRSV2/CN/110713/2018 | 139 | 774 | JXA1 | NADC30 | 2.80×10^-6^ | 6.31×10^-3^ | 1.03×10^-6^ | 1.21×10^-7^ | 7.47×10^-6^ | - | 8.87×10^-7^ |
|  | 1729 | 1897 | JXA1 | NADC30 | 1.97×10^-9^ | 2.02×10^-9^ | 1.48×10^-9^ | 8.48×10^-3^ | 6.31×10^-5^ | - | 4.52×10^-7^ |
|  | 6456 | 6955 | JXA1 | NADC30 | 2.79×10^-9^ | 3.32×10^-7^ | 2.88×10^-8^ | 2.46×10^-5^ | 7.83×10^-6^ | - | 1.73×10^-7^ |
|  | 7216 | 7622 | JXA1 | NADC30 | - | - | 2.34×10^-2^ | 6.37×10^-3^ | 3.26×10^-2^ | - | 4.98×10^-2^ |
| NADC30-R4 | | | | | | | | | | | |
| TZJ3133 | 4908 | 7667 | JXA1 | NADC30 | 2.60×10^-77^ | 1.40×10^-30^ | 1.06×10^-80^ | 2.69×10^-29^ | 4.09×10^-33^ | - | 4.44×10^-16^ |
|  | 12514 | 12778 | JXA1 | NADC30 | 7.99×10^-15^ | - | 9.04×10^-17^ | 4.30×10^-8^ | 2.53×10^-3^ | - | 4.44×10^-16^ |
| TZJ3118 | 4930 | 7675 | JXA1 | NADC30 | 2.69×10^-55^ | 8.68×10^-8^ | 3.36×10^-55^ | 2.11×10^-17^ | 1.97×10^-21^ | - | 5.55×10^-16^ |
| GXBY20220301 | 4901 | 6473 | JXA1 | NADC30 | 6.54×10^-51^ | 3.33×10^-15^ | 3.89×10^-53^ | 3.74×10^-20^ | 3.08×10^-8^ | - | 4.44×10^-16^ |
|  | 6877 | 8388 | JXA1 | NADC30 | 1.76×10^-64^ | 9.92×10^-55^ | 5.99×10^-63^ | 9.39×10^-22^ | 9.15×10^-24^ | - | 4.44×10^-16^ |
| GXGL20220301 | 4920 | 6456 | JXA1 | NADC30 | 1.40×10^-57^ | 3.94×10^-19^ | 7.49×10^-59^ | 5.33×10^-21^ | 3.08×10^-11^ | - | 4.44×10^-16^ |
|  | 6878 | 8388 | JXA1 | NADC30 | 4.40×10^-69^ | 1.52×10^-58^ | 3.33×10^-67^ | 1.90×10^-24^ | 6.95×10^-26^ | - | 4.44×10^-16^ |
| GX-3 | 4921 | 6457 | JXA1 | NADC30 | 8.54×10^-58^ | 4.01×10^-23^ | 1.96×10^-58^ | 2.69×10^-22^ | 9.17×10^-25^ | - | 5.55×10^-16^ |
|  | 6879 | 8389 | JXA1 | NADC30 | 4.54×10^-70^ | 8.73×10^-61^ | 4.91×10^-68^ | 3.78×10^-24^ | 1.46×10^-26^ | - | 1.18×10^-7^ |
|  | 10663 | 10819 | JXA1 | NADC30 | 1.07×10^-7^ | 9.53×10^-8^ | 1.15×10^-8^ | 1.46×10^-2^ | 2.43×10^-2^ | - | 1.18×10^-6^ |
| TZJ3113 | 4917 | 6457 | JXA1 | NADC30 | 4.72×10^-46^ | - | 3.68×10^-47^ | 4.79×10^-19^ | 1.25×10^-21^ | - | 4.44×10^-16^ |
|  | 6887 | 8398 | JXA1 | NADC30 | 5.34×10^-62^ | 1.12×10^-44^ | 2.08×10^-61^ | 1.45×10^-22^ | 6.74×10^-25^ | - | 3.62×10^-9^ |
|  | 10563 | 10829 | JXA1 | NADC30 | 2.75×10^-9^ | 8.08×10^-3^ | 1.05×10^-9^ | 1.67×10^-4^ | 2.86×10^-4^ | - | 1.39×10^-6^ |
| TZJ3119 | 1 | 1995 | JXA1 | NADC30 | 5.13×10^-3^ | 7.99×10^-86^ | 1.37×10^-90^ | 5.23×10^-38^ | 4.39×10^-22^ | - | 3.33×10^-16^ |
|  | 4908 | 5766 | JXA1 | NADC30 | 4.83×10^-51^ | 4.09×10^-29^ | 5.80×10^-52^ | 2.21×10^-19^ | 1.00×10^-12^ | - | 1.58×10^-13^ |
|  | 6872 | 8385 | JXA1 | NADC30 | 2.52×10^-62^ | 1.94×10^-51^ | 1.01×10^-58^ | 2.08×10^-24^ | 2.56×10^-28^ | - | 1.11×10^-16^ |
|  | 10554 | 10772 | JXA1 | NADC30 | 7.83×10^-8^ | - | 4.97×10^-8^ | 3.18×10^-5^ | 2.52×10^-4^ | - | 2.49×10^-7^ |
|  | 12600 | 12766 | JXA1 | NADC30 | 9.83×10^-18^ | 3.81×10^-15^ | 4.52×10^-18^ | 1.19×10^-8^ | 1.26×10^-8^ | - | - |
| TZJ3117 | 1 | 1996 | JXA1 | NADC30 | - | 1.33×10^-82^ | 4.09×10^-90^ | 8.26×10^-38^ | 4.50×10^-39^ | - | 3.33×10^-16^ |
|  | 4887 | 6438 | JXA1 | NADC30 | 1.82×10^-4^ | 8.51×10^-28^ | 3.75×10^-51^ | 7.20×10^-21^ | 9.56×10^-25^ | - | 4.44×10^-16^ |
|  | 6877 | 8390 | JXA1 | NADC30 | 1.36×10^-59^ | 2.27×10^-48^ | 4.17×10^-56^ | 7.15×10^-25^ | 6.67×10^-28^ | - | 1.11×10^-16^ |
|  | 10559 | 10777 | JXA1 | NADC30 | 7.29×10^-8^ | - | 4.51×10^-8^ | 3.55×10^-5^ | 2.52×10^-4^ | - | 2.38×10^-7^ |
|  | 12605 | 12757 | JXA1 | NADC30 | 1.91×10^-17^ | 1.14×10^-13^ | 8.33×10^-18^ | 6.57×10^-9^ | 8.00×10^-9^ | - | 1.26×10^-12^ |
| GD-7 | 662 | 2045 | JXA1 | NADC30 | 7.01×10^-66^ | 1.10×10^-52^ | 5.45×10^-68^ | 2.48×10^-24^ | 1.64×10^-2^ | 9.06×10^-33^ | - |
|  | 4905 | 8200 | JXA1 | NADC30 | 5.33×10^-72^ | 1.73×10^-27^ | 1.91×10^-72^ | 2.23×10^-2^ | 9.55×10^-17^ | 9.60×10^-28^ | 4.44×10^-16^ |
| NADC30-R5 | | | | | | | | | | | |
| H×10NXX-2014-3 | 1 | 2010 | JXA1 | NADC30 | 3.19×10^-114^ | 1.31×10^-94^ | 1.43×10^-113^ | 3.55×10^-33^ | 8.47×10^-6^ | - | 5.55×10^-16^ |
|  | 7324 | 7669 | JXA1 | NADC30 | 7.06×10^-17^ | 1.86×10^-9^ | 2.14×10^-16^ | 1.15×10^-6^ | 1.34×10^-2^ | - | 3.55×10^-15^ |
| H×10NXX-2014-9 | 1 | 2010 | JXA1 | NADC30 | 7.29×10^-131^ | 2.58×10^-84^ | 2.65×10^-131^ | 1.79×10^-42^ | 1.10×10^-44^ | - | 4.44×10^-16^ |
|  | 7321 | 7667 | JXA1 | NADC30 | 1.03×10^-15^ | 8.41×10^-7^ | 2.42×10^-15^ | 7.40×10^-9^ | 3.49×10^-2^ | - | 3.55×10^-15^ |
| H×10NXX-2014-12 | 450 | 2012 | JXA1 | NADC30 | 3.11×10^-116^ | 8.42×10^-108^ | 2.46×10^-119^ | 1.34×10^-35^ | 3.45×10^-37^ | - | 5.55×10^-16^ |
|  | 7273 | 7669 | JXA1 | NADC30 | 7.66×10^-15^ | 1.68×10^-7^ | 1.76×10^-14^ | 4.08×10^-9^ | 8.41×10^-9^ | - | 3.77×10^-15^ |
| 15SC3 | 1 | 2010 | JXA1 | NADC30 | 1.10×10^-126^ | 1.79×10^-116^ | 4.27×10^-128^ | 3.53×10^-34^ | 4.06×10^-4^ | - | 1.37×10^-5^ |
|  | 7304 | 7658 | JXA1 | NADC30 | 1.19×10^-18^ | 2.10×10^-13^ | 4.28×10^-19^ | 1.28×10^-8^ | 1.04×10^-9^ | - | 7.77×10^-16^ |
| H×10NXX-8 | 58 | 1506 | JXA1 | NADC30 | 1.69×10^-63^ | 8.38×10^-35^ | 7.98×10^-65^ | 1.78×10^-24^ | 1.13×10^-12^ | - | 5.55×10^-16^ |
|  | 7278 | 7672 | JXA1 | NADC30 | 6.28×10^-17^ | 1.84×10^-12^ | 7.87×10^-17^ | 2.62×10^-9^ | 9.97×10^-10^ | - | 1.67×10^-15^ |
|  | 11685 | 12880 | JXA1 | NADC30 | 1.62×10^-58^ | 3.28×10^-53^ | 1.25×10^-63^ | 2.43×10^-23^ | 1.03×10^-23^ | - | - |
| SF5 | 419 | 1822 | JXA1 | NADC30 | 1.25×10^-5^ | 3.09×10^-27^ | 2.35×10^-71^ | 3.06×10^-25^ | 4.66×10^-29^ | 4.20×10^-27^ | 2.22×10^-15^ |
| SF7 | 419 | 1822 | JXA1 | NADC30 | 7.58×10^-6^ | 5.03×10^-25^ | 4.34×10^-70^ | 1.83×10^-23^ | 4.48×10^-29^ | 5.50×10^-26^ | 2.22×10^-15^ |
| PRRSV/S020 | 164 | 1984 | JXA1 | NADC30 | 7.21×10^-116^ | 7.71×10^-106^ | 8.06×10^-115^ | 2.51×10^-31^ | 8.96×10^-36^ | - | 4.44×10^-16^ |
|  | 6177 | 6457 | JXA1 | NADC30 | 4.96×10^-6^ | - | 6.75×10^-5^ | 7.89×10^-3^ | 1.36×10^-2^ | - | 3.80×10^-4^ |
|  | 7361 | 7615 | JXA1 | NADC30 | 4.83×10^-9^ | 3.25×10^-4^ | 5.53×10^-9^ | 2.64×10^-6^ | 8.78×10^-8^ | - | 2.50×10^-8^ |
|  | 13632 | 13884 | JXA1 | NADC30 | 6.30×10^-15^ | 4.82×10^-9^ | 1.93×10^-13^ | 2.52×10^-8^ | 1.01×10^-8^ | - | 3.24×10^-12^ |
| NADC30-R6 | | | | | | | | | | | |
| GX505 | 18 | 1981 | JXA1 | NADC30 | 2.63×10^-77^ | 5.52×10^-74^ | 6.92×10^-77^ | 5.47×10^-36^ | 8.54×10^-22^ | - | - |
|  | 5297 | 7605 | JXA1 | NADC30 | 1.71×10^-99^ | 1.71×10^-92^ | 4.29×10^-103^ | 2.87×10^-33^ | 7.13×10^-23^ | - | 2.12×10^-8^ |
|  | 7765 | 8342 | JXA1 | NADC30 | 1.31×10^-32^ | 5.73×10^-24^ | 6.91×10^-34^ | 2.63×10^-11^ | 4.14×10^-13^ | - | 1.42×10^-2^ |
|  | 10766 | 10951 | JXA1 | NADC30 | 9.31×10^-8^ | 1.49×10^-5^ | 1.97×10^-7^ | 6.72×10^-3^ | 4.86×10^-3^ | - | 1.20×10^-6^ |
| GX4852 | 1 | 1961 | JXA1 | NADC30 | 2.95×10^-91^ | 5.25×10^-74^ | 7.36×10^-90^ | 6.37×10^-34^ | 1.01×10^-19^ | - | 3.33×10^-16^ |
|  | 5207 | 7585 | JXA1 | NADC30 | 2.75×10^-70^ | 8.98×10^-68^ | 2.31×10^-73^ | 3.22×10^-28^ | 6.91×10^-5^ | - | 2.21×10^-11^ |
|  | 7745 | 8322 | JXA1 | NADC30 | 7.89×10^-30^ | 5.87×10^-20^ | 1.49×10^-30^ | 9.32×10^-12^ | 6.85×10^-13^ | - | 2.10×10^-3^ |
|  | 10746 | 10931 | JXA1 | NADC30 | 4.18×10^-7^ | 3.07×10^-5^ | 1.38×10^-6^ | 2.09×10^-2^ | 1.09×10^-2^ | - | 3.75×10^-6^ |
| HN0713 | 1 | 2011 | JXA1 | NADC30 | - | 2.37×10^-71^ | 2.50×10^-76^ | 2.73×10^-27^ | 6.58×10^-37^ | - | - |
|  | 5330 | 7636 | JXA1 | NADC30 | 4.57×10^-91^ | 1.53×10^-75^ | 4.84×10^-91^ | 6.25×10^-32^ | 4.01×10^-21^ | - | 4.29×10^-10^ |
|  | 7794 | 8442 | JXA1 | NADC30 | 3.64×10^-33^ | 1.26×10^-23^ | 5.28×10^-33^ | 3.50×10^-13^ | 5.73×10^-8^ | - | 4.44×10^-16^ |
|  | 10821 | 10981 | JXA1 | NADC30 | 2.51×10^-6^ | 4.26×10^-2^ | 8.77×10^-6^ | 1.46×10^-2^ | 3.73×10^-2^ | - | 2.72×10^-4^ |
| GX1858 | 1 | 1956 | JXA1 | NADC30 | 1.98×10^-77^ | 1.95×10^-73^ | 9.05×10^-78^ | 1.57×10^-35^ | 6.21×10^-21^ | - | - |
|  | 5273 | 7581 | JXA1 | NADC30 | 1.54×10^-101^ | 1.83×10^-93^ | 2.10×10^-105^ | 1.03×10^-33^ | 6.65×10^-23^ | - | 4.44×10^-16^ |
|  | 7741 | 8333 | JXA1 | NADC30 | 4.32×10^-32^ | 4.56×10^-21^ | 8.04×10^-33^ | 1.39×10^-12^ | 1.95×10^-8^ | - | 4.44×10^-16^ |
|  | 10767 | 10971 | JXA1 | NADC30 | 9.24×10^-7^ | 2.02×10^-5^ | 5.86×10^-8^ | 5.82×10^-3^ | 8.07×10^-3^ | - | 1.57×10^-5^ |
| GXNN202004 | 1 | 2020 | JXA1 | NADC30 | - | 1.02×10^-75^ | 2.57×10^-95^ | 1.80×10^-33^ | 3.92×10^-36^ | - | 4.44×10^-16^ |
|  | 5326 | 7643 | JXA1 | NADC30 | 3.58×10^-78^ | 1.16×10^-76^ | 1.09×10^-79^ | 3.46×10^-34^ | 6.68×10^-36^ | - | 1.20×10^-12^ |
|  | 7772 | 8443 | JXA1 | NADC30 | 4.73×10^-33^ | 5.84×10^-23^ | 7.16×10^-33^ | 1.17×10^-12^ | 9.79×10^-15^ | - | 4.44×10^-16^ |
|  | 10822 | 10982 | JXA1 | NADC30 | 4.35×10^-7^ | 1.77×10^-5^ | 9.23×10^-7^ | 1.47×10^-2^ | 2.79×10^-2^ | - | 1.53×10^-5^ |
| HLJTZJ1988-2106 | 1 | 1998 | JXA1 | NADC30 | - | 1.89×10^-69^ | 4.23×10^-74^ | 1.55×10^-34^ | 7.56×10^-38^ | - | 7.47×10^-9^ |
|  | 5324 | 7644 | JXA1 | NADC30 | 8.54×10^-89^ | 2.28×10^-68^ | 8.38×10^-91^ | 2.15×10^-31^ | 5.77×10^-20^ | - | 6.46×10^-14^ |
|  | 7814 | 8442 | JXA1 | NADC30 | 2.43×10^-30^ | 4.44×10^-19^ | 2.75×10^-30^ | 2.11×10^-12^ | 8.89×10^-12^ | - | 4.44×10^-16^ |
|  | 10820 | 11024 | JXA1 | NADC30 | 1.78×10^-4^ | 4.06×10^-2^ | 1.38×10^-4^ | 2.74×10^-3^ | 3.11×10^-2^ | - | 3.08×10^-4^ |
| GZ2022 | 1 | 1612 | JXA1 | NADC30 | 1.99×10^-60^ | 3.48×10^-43^ | 9.85×10^-60^ | 1.41×10^-28^ | 9.46×10^-7^ | - | 5.39×10^-10^ |
|  | 5100 | 6188 | JXA1 | NADC30 | 6.29×10^-42^ | 5.85×10^-27^ | 1.76×10^-41^ | 2.77×10^-18^ | 9.30×10^-20^ | - | 1.03×10^-7^ |
|  | 7059 | 8244 | JXA1 | NADC30 | 2.97×10^-44^ | 8.69×10^-24^ | 9.34×10^-45^ | 9.86×10^-17^ | 1.13×10^-18^ | - | 4.44×10^-16^ |
|  | 10594 | 10874 | JXA1 | NADC30 | 4.40×10^-9^ | 2.84×10^-4^ | 4.27×10^-10^ | 1.64×10^-4^ | 9.96×10^-5^ | - | 1.18×10^-8^ |
| NADC30-R7 | | | | | | | | | | | |
| H×10B-239 | 1 | 487 | JXA1 | NADC30 | - | - | 8.51×10^-12^ | 2.83×10^-5^ | 8.85×10^-5^ | - | 2.14×10^-10^ |
|  | 672 | 1065 | JXA1 | NADC30 | 1.04×10^-29^ | 1.80×10^-22^ | 2.43×10^-31^ | 4.21×10^-8^ | 1.62×10^-4^ | - | 4.44×10^-16^ |
|  | 1309 | 1809 | JXA1 | NADC30 | 5.59×10^-31^ | 9.54×10^-18^ | 3.36×10^-32^ | 3.89×10^-12^ | 5.50×10^-14^ | - | 5.55×10^-16^ |
|  | 5182 | 6298 | JXA1 | NADC30 | 8.92×10^-40^ | 4.60×10^-22^ | 2.17×10^-42^ | 4.14×10^-19^ | 2.04×10^-21^ | - | 4.44×10^-16^ |
|  | 7477 | 8370 | JXA1 | NADC30 | 2.80×10^-37^ | 5.65×10^-30^ | 2.95×10^-37^ | 2.23×10^-16^ | 1.35×10^-19^ | - | 4.44×10^-16^ |
| TZJ3120 | 223 | 504 | JXA1 | NADC30 | 1.79×10^-2^ | - | - | 2.25×10^-2^ | 2.90×10^-3^ | - | 4.76×10^-4^ |
|  | 642 | 1054 | JXA1 | NADC30 | 5.84×10^-24^ | 3.40×10^-13^ | 1.95×10^-23^ | 1.16×10^-4^ | 5.98×10^-6^ | - | 4.44×10^-16^ |
|  | 1283 | 1786 | JXA1 | NADC30 | 7.61×10^-19^ | 6.74×10^-4^ | 1.65×10^-19^ | 2.98×10^-12^ | 2.97×10^-5^ | - | 4.44×10^-16^ |
|  | 5145 | 6338 | JXA1 | NADC30 | 1.24×10^-33^ | 5.98×10^-18^ | 7.44×10^-35^ | 1.91×10^-18^ | 3.30×10^-19^ | - | 2.33×10^-6^ |
|  | 7442 | 8345 | JXA1 | NADC30 | 2.41×10^-34^ | 2.62×10^-17^ | 2.32×10^-33^ | 1.04×10^-16^ | 1.22×10^-18^ | - | 4.44×10^-16^ |
| HLJWK108-1711 | 1 | 476 | JXA1 | NADC30 | - | 3.01×10^-4^ | 6.82×10^-12^ | 7.16×10^-6^ | 2.08×10^-6^ | - | 6.02×10^-10^ |
|  | 630 | 1066 | JXA1 | NADC30 | 2.00×10^-33^ | 1.13×10^-29^ | 1.15×10^-34^ | 5.99×10^-9^ | 1.55×10^-5^ | - | 4.44×10^-16^ |
|  | 1310 | 1810 | JXA1 | NADC30 | 9.88×10^-30^ | 2.83×10^-16^ | 4.94×10^-31^ | 7.11×10^-14^ | 1.09×10^-14^ | - | 4.44×10^-16^ |
|  | 5184 | 6380 | JXA1 | NADC30 | 2.99×10^-45^ | 1.14×10^-30^ | 3.25×10^-45^ | 1.46×10^-20^ | 3.12×10^-23^ | - | 4.44×10^-16^ |
|  | 7478 | 8386 | JXA1 | NADC30 | 1.45×10^-40^ | 9.60×10^-35^ | 6.02×10^-40^ | 5.05×10^-19^ | 2.93×10^-10^ | - | 4.44×10^-16^ |
| TZJ1713 | 1 | 482 | JXA1 | NADC30 | 3.13×10^-15^ | 5.30×10^-5^ | 2.51×10^-16^ | 4.70×10^-5^ | 3.75×10^-6^ | - | 9.16×10^-12^ |
|  | 1335 | 1495 | JXA1 | NADC30 | 4.68×10^-19^ | 1.87×10^-6^ | 6.22×10^-17^ | 1.47×10^-8^ | 1.60×10^-9^ | - | 6.33×10^-15^ |
|  | 5169 | 6353 | JXA1 | NADC30 | 1.06×10^-45^ | 5.25×10^-14^ | 8.07×10^-45^ | 1.18×10^-19^ | 4.95×10^-22^ | - | 5.55×10^-16^ |
|  | 7475 | 8428 | JXA1 | NADC30 | 3.97×10^-48^ | 6.63×10^-36^ | 9.72×10^-50^ | 1.23×10^-18^ | 8.29×10^-21^ | - | 3.23×10^-11^ |
| CHN-HB-2018 | 45 | 475 | JXA1 | NADC30 | 1.75×10^-11^ | 2.94×10^-4^ | 4.47×10^-11^ | 1.54×10^-5^ | 7.54×10^-6^ | - | 2.33×10^-9^ |
|  | 630 | 1064 | JXA1 | NADC30 | 1.68×10^-32^ | 1.72×10^-21^ | 3.74×10^-33^ | 1.52×10^-11^ | 2.24×10^-15^ | - | 0.010183651 |
|  | 5167 | 6363 | JXA1 | NADC30 | 3.96×10^-42^ | 2.67×10^-26^ | 3.58×10^-41^ | 3.61×10^-20^ | 1.03×10^-20^ | - | 4.44×10^-16^ |
|  | 7462 | 8370 | JXA1 | NADC30 | 5.23×10^-45^ | 6.40×10^-31^ | 3.33×10^-45^ | 1.41×10^-18^ | 6.39×10^-12^ | - | 5.55×10^-16^ |
| NADC30-R8 | | | | | | | | | | | |
| HNhx | 4888 | 7602 | JXA1 | NADC30 | 9.82×10^-124^ | 2.81×10^-90^ | 9.19×10^-127^ | 9.87×10^-36^ | 3.57×10^-38^ | - | 5.55×10^-16^ |
| 15H×10N1 | 4888 | 7602 | JXA1 | NADC30 | 4.21×10^-151^ | 3.79×10^-132^ | 6.25×10^-152^ | 9.41×10^-40^ | 1.26×10^-41^ | - | 5.55×10^-16^ |
| CH/SCPZ/2020 | 4888 | 7602 | JXA1 | NADC30 | 1.39×10^-125^ | 9.19×10^-87^ | 1.40×10^-126^ | 5.52×10^-36^ | 5.00×10^-39^ | - | 5.55×10^-16^ |
| PRRSV/HB94 | 5012 | 7626 | JXA1 | NADC30 | 1.66×10^-99^ | 4.36×10^-48^ | 1.68×10^-96^ | 6.16×10^-31^ | 8.14×10^-11^ | - | 5.55×10^-16^ |
| MZ160905.1 | 4888 | 7602 | JXA1 | NADC30 | 1.61×10^-127^ | 2.82×10^-97^ | 9.79×10^-131^ | 3.62×10^-33^ | 1.22×10^-38^ | - | 5.55×10^-16^ |
| NADC30-R9 | | | | | | | | | | | |
| HM1809 | 48 | 540 | JXA1 | NADC30 | 2.39×10^-14^ | 4.79×10^-8^ | 1.98×10^-15^ | 6.96×10^-7^ | 4.69×10^-3^ | - | 6.88×10^-15^ |
|  | 1356 | 1499 | JXA1 | NADC30 | 2.19×10^-13^ | 1.48×10^-4^ | 6.30×10^-14^ | 4.53×10^-7^ | 2.30×10^-4^ | - | 6.42×10^-10^ |
|  | 4895 | 7654 | JXA1 | NADC30 | 1.62×10^-89^ | 1.20×10^-38^ | 7.85×10^-90^ | 8.71×10^-31^ | 1.55×10^-34^ | - | 4.44×10^-16^ |
| TZJ3112 | 1 | 563 | JXA1 | NADC30 | 7.30×10^-19^ | - | 9.03×10^-20^ | 1.35×10^-8^ | 1.76×10^-9^ | - | 4.44×10^-16^ |
|  | 1358 | 2001 | JXA1 | NADC30 | 3.64×10^-53^ | 7.75×10^-23^ | 5.65×10^-50^ | 8.25×10^-19^ | 1.11×10^-11^ | - | 4.44×10^-16^ |
|  | 4980 | 7646 | JXA1 | NADC30 | 2.17×10^-75^ | 3.55×10^-30^ | 1.56×10^-39^ | 2.06×10^-31^ | 1.21×10^-9^ | - | 4.44×10^-16^ |
| TZJ3114 | 45 | 532 | JXA1 | NADC30 | 1.13×10^-12^ | 5.13×10^-5^ | 1.04×10^-12^ | 1.15×10^-7^ | 8.72×10^-4^ | - | 2.34×10^-02^ |
|  | 1346 | 2002 | JXA1 | NADC30 | 2.86×10^-39^ | 3.63×10^-11^ | 7.78×10^-38^ | 1.00×10^-16^ | 6.82×10^-19^ | - | 4.44×10^-16^ |
|  | 4978 | 7644 | JXA1 | NADC30 | 2.87×10^-70^ | 2.83×10^-22^ | 5.80×10^-71^ | 3.71×10^-29^ | 1.91×10^-32^ | - | 4.44×10^-16^ |
| TZJ3131 | 1 | 559 | JXA1 | NADC30 | 1.86×10^-16^ | 2.65×10^-11^ | 2.18×10^-17^ | 2.60×10^-8^ | 3.22×10^-9^ | 4.90×10^-8^ | 2.62×10^-14^ |
|  | 1358 | 2013 | JXA1 | NADC30 | 5.75×10^-33^ | 4.92×10^-20^ | 1.00×10^-29^ | 2.98×10^-15^ | 2.59×10^-18^ | 2.45×10^-15^ | 8.88×10^-16^ |
|  | 4980 | 7199 | JXA1 | NADC30 | 1.56×10^-18^ | 1.45×10^-40^ | 3.13×10^-45^ | 5.51×10^-23^ | 1.25×10^-14^ | 9.35×10^-36^ | 4.44×10^-16^ |
|  | 7208 | 10234 | VR2332 | NADC30 | 6.06×10^-113^ | 1.87×10^-129^ | 6.93×10^-111^ | 3.42×10^-34^ | 5.77×10^-27^ | 6.69×10^-55^ | 9.77×10^-15^ |
|  | 10254 | 11658 | JXA1 | NADC30 | 9.53×10^-31^ | 2.75×10^-17^ | 9.31×10^-32^ | 2.01×10^-19^ | 5.44×10^-7^ | 2.29×10^-22^ | 9.20×10^-13^ |
| NADC30-R10 | | | | | | | | | | | |
| SDYG1606 | 1 | 2010 | JXA1 | NADC30 | - | 3.45×10-^107^ | 2.74×10^-117^ | 9.47×10^-37^ | 1.50×10^-38^ | - | 4.44×10^-16^ |
|  | 4994 | 8378 | JXA1 | NADC30 | 1.89×10^-106^ | 1.08×10^-109^ | 9.47×10^-106^ | 4.93×10^-43^ | 1.85×10^-45^ | - | 3.33×10^-16^ |
| sg_2107 | 1 | 1845 | JXA1 | NADC30 | 4.12×10^-86^ | 3.10×10^-60^ | 1.31×10^-85^ | 9.32×10^-31^ | 3.65×10^-33^ | - | 5.43×10^-10^ |
|  | 4829 | 8327 | JXA1 | NADC30 | 3.71×10^-93^ | 5.37×10^-85^ | 2.48×10^-94^ | 1.38×10^-39^ | 7.32×10^-22^ | - | 2.22×10^-16^ |
| PRRSV/H012 | 1 | 2000 | JXA1 | NADC30 | 9.61×10^-95^ | 6.93×10^-77^ | 3.27×10^-94^ | 2.31×10^-33^ | 1.63×10^-35^ | - | 4.44×10^-16^ |
|  | 4984 | 8640 | JXA1 | NADC30 | 3.06×10^-77^ | 2.13×10^-65^ | 3.60×10^-77^ | 9.26×10^-40^ | 2.05×10^-42^ | - | 2.22×10^-16^ |
|  | 13547 | 13722 | JXA1 | NADC30 | 6.66×10^-14^ | 1.02×10^-8^ | 1.29×10^-10^ | 7.77×10^-7^ | 3.85×10^-3^ | - | 7.28×10^-12^ |
| BDSP-1 | 1 | 2020 | JXA1 | NADC30 | 5.95×10^-57^ | 1.49×10^-10^ | 9.15×10^-58^ | 3.60×10^-30^ | 2.68×10^-15^ | - | 3.33×10^-16^ |
|  | 4996 | 8536 | JXA1 | NADC30 | 4.63×10^-50^ | 1.90×10^-13^ | 1.91×10^-40^ | 4.72×10^-32^ | 2.74×10^-36^ | - | 2.22×10^-16^ |
|  | 13544 | 13760 | JXA1 | NADC30 | 3.21×10^-9^ | 6.46×10^-4^ | 9.28×10^-10^ | 2.99×10^-4^ | 1.23×10^-6^ | - | 1.22×10^-8^ |
| NADC30-R11 | | | | | | | | | | | |
| 15JX1 | 1192 | 2064 | JXA1 | NADC30 | 3.10×10^-88^ | 1.94×10^-74^ | 4.59×10^-88^ | 9.19×10^-28^ | 1.09×10^-28^ | - | 5.55×10^-16^ |
| H×10NAN-H×10B | 1359 | 2136 | JXA1 | NADC30 | 4.88×10^-74^ | 1.02×10^-53^ | 2.27×10^-73^ | 1.17×10^-24^ | 3.43×10^-26^ | - | 6.66×10^-16^ |
| H×10NLH2017 | 1190 | 1930 | JXA1 | NADC30 | 6.15×10^-98^ | 3.36×10^-99^ | 9.74×10^-101^ | 1.74×10^-21^ | 2.51×10^-27^ | - | 5.55×10^-16^ |
| NADC30-R12 | | | | | | | | | | | |
| SC/DJY | 7378 | 8387 | JXA1 | NADC30 | 6.42×10^-54^ | 2.67×10^-49^ | 1.05×10^-53^ | 5.05×10^-19^ | 1.71×10^-19^ | - | 5.55×10^-16^ |
|  | 10566 | 11072 | JXA1 | NADC30 | 1.87×10^-25^ | 3.91×10^-21^ | 2.05×10^-25^ | 1.33×10^-11^ | 6.05×10^-12^ | - | 5.55×10^-16^ |
|  | 11352 | 11790 | JXA1 | NADC30 | 2.05×10^-25^ | 1.01×10^-23^ | 5.06×10^-27^ | 5.71×10^-9^ | 1.31×10^-9^ | - | 5.55×10^-16^ |
| SCABTC-202305 | 7449 | 8359 | JXA1 | NADC30 | 2.65×10^-54^ | 1.40×10^-49^ | 4.22×10^-54^ | 2.16×10^-17^ | 2.97×10^-19^ | - | 5.55×10^-16^ |
|  | 10539 | 11044 | JXA1 | NADC30 | 7.93×10^-28^ | 5.48×10^-26^ | 1.16×10^-27^ | 1.92×10^-12^ | 9.30×10^-13^ | - | 5.55×10^-16^ |
|  | 11325 | 11763 | JXA1 | NADC30 | 4.69×10^-27^ | 4.39×10^-25^ | 1.18×10^-28^ | 1.09×10^-11^ | 2.81×10^-12^ | - | 5.55×10^-16^ |
| NADC30-IR | | | | | | | | | | | |
| SDHY_DZ037 | 6978 | 7623 | IA/2014/NADC34 | NADC30 | 3.44×10^-30^ | - | 5.02×10^-18^ | 1.23×10^-19^ | 6.36×10^-17^ | 5.21×10^-40^ | 4.44×10^-16^ |
|  | 7830 | 8146 | JXA1 | NADC30 | 2.19×10^-18^ | 5.00×10^-12^ | 1.91×10^-16^ | 3.81×10^-9^ | 1.00×10^-10^ | 4.00×10^-17^ | - |
| GXNN20210506 | 1 | 506 | JXA1 | NADC30 | 4.14×10-^49^ | 1.50×10^-25^ | 1.83×10^-51^ | 4.95×10^-17^ | 6.25×10^-16^ | 1.51×10^-39^ | 4.44×10^-16^ |
|  | 394 | 2016 | JXA1 | NADC30 | 9.36×10^-46^ | 1.78×10^-22^ | 1.72×10^-42^ | 3.43×10^-25^ | 6.55×10^-29^ | 1.95×10^-25^ | 9.68×10^-11^ |
|  | 7934 | 8889 | JXA1 | NADC30 | 1.30×10^-19^ | 2.36×10^-35^ | 4.47×10^-40^ | 1.26×10^-14^ | 4.43×10^-11^ | 7.53×10^-3^ | 1.33×10^-15^ |
|  | 12244 | 15547 | IA/2014/NADC34 | NADC30 | 8.70×10^-10^ | - | 1.09×10^-9^ | - | - | 4.57×10^-3^ | 2.97×10^-8^ |
| GXFCG20210401 | 1 | 502 | JXA1 | NADC30 | 7.38×10^-12^ | 2.21×10^-3^ | 1.21×10^-11^ | - | - | 5.95×10^-4^ | 2.22×10^-2^ |
|  | 694 | 2015 | JXA1 | NADC30 | 3.37×10^-46^ | - | 9.45×10^-43^ | 2.53×10^-25^ | 4.38×10^-29^ | 3.89×10^-25^ | 1.84×10^-12^ |
|  | 7934 | 8948 | JXA1 | NADC30 | 1.47×10^-42^ | 6.44×10^-40^ | 2.00×10^-43^ | 1.22×10^-18^ | 4.21×10^-4^ | 2.17×10^-19^ | 1.63×10^-4^ |
|  | 23334 | 15547 | IA/2014/NADC34 | NADC30 | 4.00×10^-50^ | 7.03×10^-29^ | 3.06×10^-51^ | 7.91×10^-33^ | 1.96×10^-8^ | 9.50×10^-40^ | 4.44×10^-16^ |
| SCABTC-202309 | 1 | 568 | JXA1 | NADC30 | 7.05×10^-13^ | 1.19×10^-3^ | 1.03×10^-13^ | 1.82×10^-6^ | 1.76×10^-7^ | 1.43×10^-6^ | 6.19×10^-12^ |
|  | 1358 | 2016 | JXA1 | NADC30 | 4.14×10^-36^ | 2.05×10^-16^ | 8.78×10^-35^ | 1.17×10^-15^ | 6.01×10^-17^ | 9.59×10^-13^ | 1.55×10^-14^ |
|  | 7260 | 8276 | JXA1 | NADC30 | 3.00×10^-38^ | 8.02×10^-24^ | 6.21×10^-41^ | 2.06×10^-11^ | 8.11×10^-16^ | 1.13×10^-19^ | 1.55×10^-14^ |
| CH-WH-2019-1 | 6731 | 8138 | JXA1 | NADC30 | 2.16×10^-74^ | 5.10×10^-55^ | 1.39×10^-73^ | 1.32×10^-19^ | 5.64×10^-22^ | 8.12×10^-30^ | 3.52×10^-3^ |
|  | 9282 | 9544 | VR2332 | NADC30 | 1.85×10^-16^ | 1.45×10^-15^ | 1.29×10^-12^ | 4.19×10^-7^ | 5.83×10^-3^ | 2.95×10^-5^ | 4.41×10^-11^ |
| PRRSV/H013 | 5876 | 6577 | VR2332 | NADC30 | 5.77×10^-35^ | 7.21×10^-21^ | 2.28×10^-36^ | 2.23×10^-10^ | 6.02×10^-14^ | 1.37×10^-16^ | 1.78×10^-15^ |
|  | 7092 | 8148 | JXA1 | NADC30 | 6.67×10^-65^ | 5.59×10^-48^ | 7.25×10^-65^ | 6.02×10^-20^ | 6.41×10^-21^ | 1.55×10^-22^ | 2.22×10^-15^ |
| SDQD-1604 | 5876 | 6421 | VR2332 | NADC30 | 3.04×10^-34^ | 1.88×10^-30^ | 1.80×10^-35^ | 2.93×10^-12^ | 2.68×10^-11^ | 1.16×10^-13^ | 1.55×10^-14^ |
|  | 6720 | 8145 | JXA1 | NADC30 | 7.67×10^-87^ | 4.15×10^-76^ | 2.33×10^-86^ | 4.77×10^-23^ | 4.66×10^-12^ | 6.63×10^-32^ | 2.80×10^-3^ |
| PRRSV/S136 | 5876 | 6421 | VR2332 | NADC30 | 2.47×10^-25^ | 2.58×10^-13^ | 2.90×10^-25^ | 3.29×10^-9^ | 7.52×10^-10^ | 5.44×10^-12^ | 1.55×10^-14^ |
|  | 6770 | 8138 | JXA1 | NADC30 | 2.01×10^-50^ | 1.02×10^-19^ | 3.64×10^-43^ | 2.30×10^-16^ | 6.15×10^-3^ | 2.90×10^-15^ | 6.53×10^-6^ |
| HNTZJ1715-2102 | 7262 | 8946 | JXA1 | NADC30 | 1.83×10^-95^ | 2.01×10^-81^ | 5.87×10^-97^ | 4.08×10^-25^ | 3.62×10^-7^ | - | 5.55×10^-16^ |
| HM1805 | 7238 | 8748 | JXA1 | NADC30 | 1.08×10^-100^ | 1.16×10^-98^ | 2.23×10^-100^ | 1.21×10^-27^ | 1.87×10^-28^ | - | 5.55×10^-16^ |
|  | 10862 | 11178 | JXA1 |  | 1.37×10^-13^ | 1.10×10^-12^ | 2.87×10^-14^ | 1.10×10^-7^ | 3.87×10^-8^ | - | 1.40×10^-11^ |
| FS-GD-02 | 6730 | 9801 | JXA1 | NADC30 | 1.57×10^-129^ | 1.29×10^-105^ | 5.18×10^-132^ | 5.06×10^-36^ | 6.17×10^-38^ | - | 5.55×10^-16^ |
|  | 11054 | 11382 | JXA1 |  | 2.08×10^-16^ | 6.60×10^-13^ | 3.78×10^-16^ | 5.33×10^-8^ | 7.73×10^-8^ | - | 3.54×10^-11^ |
| PRRSV2/CN/F2/2019 | 5778 | 8889 | JXA1 | NADC30 | 2.49×10^-155^ | 6.28×10^-158^ | 4.04×10^-159^ | 2.37×10^-43^ | 7.53×10^-44^ | 2.32×10^-57^ | 1.33×10^-15^ |
|  | 12043 | 13608 | QYYZ |  | 6.04×10^-36^ | - | 7.56×10^-39^ | 1.64×10^-18^ | 3.03×10^-11^ | 1.12×10^-15^ | 1.78×10^-15^ |
| FJWQ16 | 6967 | 8889 | JXA1 | NADC30 | 7.42×10^-101^ | 1.48×10^-88^ | 2.59×10^-97^ | 6.73×10^-29^ | 5.32×10^-31^ | 6.43×10^-40^ | - |
|  | 12438 | 12880 | QYYZ |  | 7.22×10^-17^ | 1.09×10^-4^ | 2.14×10^-29^ | 4.01×10^-4^ | - | 2.05×10^-4^ | - |
| PRRSV2/CN/N2/2021 | 5896 | 6818 | QYYZ | NADC30 | 1.83×10^-28^ | - | 9.22×10^-28^ | 1.00×10^-15^ | 2.19×10^-15^ | 2.31×10^-13^ | 1.33×10^-15^ |
|  | 8244 | 9384 | JXA1 |  | 1.60×10^-22^ | 6.02×10^-19^ | 6.18×10^-18^ | 1.59×10^-3^ | 7.41×10^-12^ | 1.03×10^-17^ | 1.33×10^-15^ |
| PRRSV2/CN/S5/2018 | 43 | 428 | JXA1 | NADC30 | 4.29×10^-7^ | - | 2.70×10^-7^ | 2.61×10^-4^ | 1.88×10^-2^ | 8.82×10^-9^ | 7.33×10^-6^ |
|  | 1154 | 1711 | JXA1 | NADC30 | 2.97×10^-11^ | 8.23×10^-7^ | 2.82×10^-11^ | 5.38×10^-7^ | 1.43×10^-8^ | 1.34×10^-2^ | 3.45×10^-10^ |
|  | 5598 | 5823 | JXA1 | NADC30 | 1.29×10^-8^ | 1.76×10^-7^ | 2.38×10^-10^ | 6.71×10^-5^ | 5.63×10^-6^ | - | 3.47×10^-9^ |
|  | 5824 | 6442 | QYYZ | NADC30 | 3.75×10^-18^ | 5.72×10^-12^ | 1.22×10^-15^ | 8.50×10^-3^ | 3.71×10^-3^ | 3.91×10^-7^ | 1.78×10^-15^ |
|  | 6681 | 6972 | QYYZ | NADC30 | 2.20×10^-6^ | - | 3.38×10^-7^ | 3.98×10^-4^ | 1.16×10^-4^ | - | 1.83×10^-8^ |
|  | 7653 | 9864 | JXA1 | NADC30 | 1.26×10^-69^ | 1.03×10^-68^ | 3.64×10^-47^ | 2.71×10^-17^ | 4.85×10^-20^ | 7.51×10^-39^ | 1.33×10^-15^ |
|  | 9959 | 10190 | JXA1 | NADC30 | 1.33×10^-10^ | 2.41×10^-15^ | 9.44×10^-13^ | 8.01×10^-5^ | 2.92×10^-4^ | 9.22×10^-5^ | 6.79×10^-10^ |
|  | 12746 | 12960 | JXA1 | NADC30 | 2.25×10^-8^ | 2.57×10^-5^ | 8.28×10^-9^ | 1.84×10^-7^ | 1.29×10^-7^ | 8.38×10^-5^ | 4.19×10^-9^ |
|  | 13094 | 13380 | JXA1 | NADC30 | 7.05×10^-15^ | 1.10×10^-13^ | 3.21×10^-17^ | 3.08×10^-4^ | - | 3.59×10^-10^ | - |
| PRRSV2/CN/X2998/2018 | 1 | 576 | JXA1 | NADC30 | 8.88×10^-10^ | - | 1.16×10^-9^ | 2.64×10^-4^ | 3.90×10^-8^ | - | - |
|  | 1154 | 1711 | JXA1 | NADC30 | 6.12×10^-22^ | - | 3.71×10^-23^ | 3.38×10^-17^ | 1.73×10^-17^ | 2.24×10^-13^ | 1.33×10^-15^ |
|  | 5598 | 5856 | JXA1 | NADC30 | 5.66×10^-10^ | 5.69×10^-8^ | 3.18×10^-11^ | 1.14×10^-3^ | 1.11×10^-4^ | 2.88×10^-2^ | 2.25×10^-9^ |
|  | 5869 | 6550 | QYYZ | NADC30 | 9.56×10^-23^ | 7.13×10^-12^ | 3.53×10^-21^ | 1.29×10^-4^ | 4.21×10^-4^ | 5.38×10^-9^ | 1.78×10^-15^ |
|  | 6681 | 6972 | QYYZ | NADC30 | 3.80×10^-7^ | - | 4.16×10^-8^ | 3.20×10^-4^ | 6.24×10^-4^ | - | 8.19×10^-9^ |
|  | 7653 | 10190 | JXA1 | NADC30 | 6.20×10^-64^ | 1.86×10^-63^ | 2.66×10^-43^ | 1.92×10^-17^ | 1.89×10^-23^ | 7.51×10^-39^ | 1.33×10^-15^ |
|  | 12746 | 13380 | JXA1 | NADC30 | 3.24×10^-9^ | 1.14×10^-5^ | 1.04×10^-9^ | 5.41×10^-6^ | 1.28×10^-5^ | 8.38×10^-5^ | 5.04×10^-10^ |
|  | 13907 | 14208 | JXA1 | NADC30 | 1.69×10^-5^ | 1.49×10^-3^ | 1.39×10^-3^ | 1.64×10^-2^ | - | 1.49×10^-5^ | 1.02×10^-2^ |
| FJDJQ-2018 | 1 | 1290 | JXA1 | NADC30 | 5.28×10^-41^ | 4.33×10^-25^ | 7.06×10^-42^ | 1.35×10^-20^ | 7.47×10^-3^ | 3.12×10^-22^ | - |
|  | 3960 | 6819 | QYYZ | NADC30 | 6.24×10^-49^ | - | 5.94×10^-44^ | 4.73×10^-22^ | 4.48×10^-25^ | 2.43×10^-22^ | 1.33×10^-15^ |
|  | 7456 | 9742 | JXA1 | NADC30 | 2.36×10^-34^ | - | 9.56×10^-35^ | 3.19×10^-19^ | 1.79×10^-10^ | 1.71×10^-17^ | 1.78×10^-15^ |
|  | 13064 | 13854 | JXA1 | NADC30 | 6.22×10^-11^ | 2.37×10^-9^ | 2.69×10^-9^ | 6.32×10^-13^ | 3.47×10^-10^ | - | - |
| FJDJQ-2018 | 1 | 1290 | JXA1 | NADC30 | 5.28×10^-41^ | 4.74×10^-25^ | 7.06×10^-42^ | 1.35×10^-20^ | 7.47×10^-3^ | 3.12×10^-22^ | - |
|  | 3960 | 6819 | QYYZ | NADC30 | 6.24×10^-49^ | - | 5.94×10^-44^ | 4.73×10^-22^ | 4.48×10^-25^ | 2.44×10^-22^ | 1.33×10^-15^ |
|  | 7456 | 8670 | JXA1 | NADC30 | 2.36×10^-34^ | - | 9.56×10^-35^ | 3.19×10^-19^ | 1.79×10^-10^ | 1.71×10^-17^ | 1.78×10^-15^ |
|  | 8671 | 9742 | QYYZ | NADC30 | 6.22×10^-11^ | 2.37×10^-9^ | 2.69×10-9 | 6.32×10^-13^ | 3.47×10^-10^ | - | - |
| PRRSV2/CN/N4/2019 | 4327 | 6521 | QYYZ | NADC30 | 1.53×10^-36^ | 9.09×10^-16^ | 9.47×10^-21^ | 1.24×10^-30^ | 7.78×10^-11^ | 1.41×10^-57^ | 1.18×10^-7^ |
|  | 7341 | 8278 | JXA1 | NADC30 | 1.08×10^-2^ | - | 2.48×10^-2^ | 1.04×10^-4^ | 5.56×10^-4^ | 2.49×10^-2^ | - |
|  | 8368 | 8654 | JXA1 | NADC30 | 3.43×10^-6^ | 1.15×10^-5^ | 1.37×10^-6^ | 6.34×10^-8^ | 1.70×10^-3^ | 8.15×10^-12^ | - |
| PRRSV2/CN/N42/2017 | 1062 | 1255 | JXA1 | NADC30 | 6.42×10^-12^ | 4.09×10^-10^ | 1.67×10^-11^ | 1.83×10^-5^ | 3.92×10^-6^ | 2.77×10^-2^ | - |
|  | 4333 | 6644 | QYYZ | NADC30 | 2.61×10^-15^ | - | 3.32×10^-10^ | 7.01×10^-35^ | 1.44×10^-35^ | 1.96×10^-13^ | 1.78×10^-15^ |
|  | 7456 | 8529 | JXA1 | NADC30 | 1.14×10^-8^ | - | 2.82×10^-9^ | 4.02×10^-11^ | 2.55×10^-6^ | 1.01×10^-13^ | 9.39×10^-5^ |
|  | 12636 | 12829 | QYYZ | NADC30 | 9.04×10^-9^ | 2.22×10^-5^ | 1.79×10^-12^ | 2.54×10^-2^ | 1.16×10^-3^ | - | 1.44×10^-11^ |
|  | 12915 | 13237 | QYYZ | NADC30 | 9.74×10^-13^ | - | 4.87×10^-21^ | - | - | 6.15×10^-4^ | 1.78×10^-15^ |
| PRRSV2/CN/Z8/2018 | 4327 | 6481 | QYYZ | NADC30 | 7.67×10^-40^ | 2.53×10^-16^ | 8.07×10^-28^ | 4.50×10^-35^ | 1.86×10^-34^ | 2.94×10^-57^ | 2.51×10^-8^ |
|  | 7456 | 8941 | JXA1 | NADC30 | 3.55×10^-34^ | 1.17×10^-16^ | 1.00×10^-35^ | 1.90×10^-14^ | 3.50×10^-21^ | 4.34×10^-32^ | 4.44×10^-16^ |
|  | 12438 | 12882 | QYYZ | NADC30 | 1.83×10^-12^ | 2.30×10^-6^ | 1.33×10^-12^ | 1.16×10^-8^ | 2.03×10^-9^ | 6.10×10^-10^ | - |
| PRRSV2/CN/N0/2021 | 4321 | 6819 | QYYZ | NADC30 | 6.31×10^-18^ | 2.79×10^-8^ | 1.39×10^-17^ | 7.85×10^-19^ | 1.94×10^-11^ | 7.57×10^-19^ | 2.22×10^-15^ |
|  | 7446 | 8774 | JXA1 | NADC30 | 4.04×10^-46^ | 2.07×10^-36^ | 5.85×10^-26^ | 2.52×10^-21^ | 1.04×10^-10^ | 3.47×10^-56^ | 3.47×10^-8^ |
|  | 8806 | 9366 | QYYZ | NADC30 | 2.84×10^-36^ | 4.13×10^-17^ | 5.88×10^-38^ | 9.44×10^-25^ | 1.28×10^-21^ | 8.64×10^-32^ | 8.88×10^-16^ |
| PRRSV2/CN/X4836/2018 | 4330 | 6562 | QYYZ | NADC30 | 6.54×10^-31^ | 1.91×10^-15^ | 1.72×10^-32^ | 1.33×10^-23^ | 4.91×10^-22^ | 8.64×10^-32^ | 4.44×10^-16^ |
|  | 6800 | 8537 | JXA1 | NADC30 | 4.99×10^-5^ | 5.94×10^-3^ | 2.97×10^-4^ | 4.46×10^-4^ | 1.29×10^-4^ | 3.47×10^-6^ | - |
| FJLIUY-2017 | 15512 | 744 | JXA1 | NADC30 | 6.78×10^-20^ | 4.05×10^-9^ | 3.06×10^-21^ | 1.66×10^-10^ | 3.45×10^-13^ | 2.37×10^-11^ | 3.33×10^-15^ |
|  | 1286 | 1828 | JXA1 | NADC30 | 1.55×10^-26^ | 2.27×10^-16^ | 7.38×10^-28^ | 2.81×10^-16^ | 2.17×10^-16^ | 6.29×10^-15^ | 3.33×10^-15^ |
|  | 6865 | 9536 | JXA1 | NADC30 | 1.30×10^-45^ | 4.86×10^-19^ | 2.54×10^-25^ | 1.70×10^-28^ | 4.67×10^-31^ | 4.02×10^-22^ | 2.22×10^-15^ |
|  | 11091 | 10588 | JXA1 | NADC30 | 8.98×10^-16^ | 1.53×10^-8^ | 1.81×10^-15^ | 1.09×10^-9^ | 4.34×10^-2^ | 5.58×10^-7^ | 3.06×10^-13^ |
|  | 12062 | 12623 | QYYZ | NADC30 | 8.41×10^-16^ | 4.14×10^-7^ | 8.67×10^-16^ | 1.66×10^-7^ | 6.36×10^-8^ | 3.09×10^-8^ | 4.44×10^-15^ |
|  | 12624 | 13280 | VR2332 | NADC30 | 2.80×10^-35^ | 8.31×10^-33^ | 6.47×10^-30^ | 3.88×10^-14^ | 1.06×10^-4^ | 2.31×10^-18^ | 3.33×10^-15^ |
|  | 8763 | 8997 | VR2332 | JXA1 | 9.20×10^-18^ | 1.53×10^-16^ | 1.15×10^-12^ | 9.46×10^-8^ | 5.01×10^-6^ | 3.66×10^-3^ | 2.59×10^-11^ |
|  | 13856 | 15220 | QYYZ | NADC30 | 6.46×10^-25^ | - | 2.71×10^-22^ | 2.54×10^-11^ | 1.93×10^-2^ | 8.56×10^-12^ | 4.44×10^-15^ |
| ZJqz21 | 1 | 1812 | JXA1 | NADC30 | 2.03×10^-64^ | 7.37×10^-57^ | 6.55×10^-61^ | 4.87×10^-34^ | 1.06×10^-33^ | 2.60×10^-35^ | - |
|  | 5890 | 6809 | VR2332 | NADC30 | 1.66×10^-39^ | 6.16×10^-27^ | 4.48×10^-38^ | 1.35×10^-16^ | 4.27×10^-18^ | 2.45×10^-24^ | 3.33×10^-15^ |
|  | 7980 | 8426 | JXA1 | NADC30 | 2.74×10^-6^ | - | 1.16×10^-5^ | 6.66×10^-3^ | 5.66×10^-3^ | 1.19×10^-2^ | 1.84×10^-3^ |
|  | 8477 | 9548 | JXA1 | NADC30 | 8.12×10^-40^ | 1.87×10^-20^ | 4.12×10^-39^ | 1.61×10^-16^ | 1.33×10^-18^ | 9.37×10^-15^ | 4.44×10^-15^ |
|  | 11098 | 11496 | JXA1 | NADC30 | 1.38×10^-17^ | 1.94×10^-9^ | 4.90×10^-17^ | 7.78×10^-10^ | 2.08×10^-10^ | 5.59×10^-7^ | 8.88×10^-15^ |
|  | 11973 | 12187 | JXA1 | NADC30 | 7.14×10^-8^ | 5.07×10^-5^ | 6.93×10^-8^ | 4.78×10^-3^ | 1.24×10^-3^ | - | 6.94×10^-7^ |
| SD-1602 | 1 | 2028 | JXA1 | NADC30 | - | 3.29×10^-92^ | 5.52×10^-96^ | 6.03×10^-34^ | 2.48×10^-39^ | - | 3.33×10^-16^ |
|  | 5616 | 7960 | JXA1 | NADC30 | 5.95×10^-72^ | 1.68×10^-17^ | 6.32×10^-73^ | 2.25×10^-27^ | 7.97×10^-10^ | - | 4.44×10^-16^ |
| 15LN3 | 1 | 700 | JXA1 | NADC30 | 1.89×10^-37^ | 2.26×10^-32^ | 9.98×10^-38^ | 1.68×10^-13^ | 1.74×10^-15^ | - | 1.51×10^-2^ |
|  | 1358 | 2016 | JXA1 | NADC30 | 7.22×10^-51^ | 8.55×10^-36^ | 1.49×10^-51^ | 3.75×10^-19^ | 4.30×10^-21^ | - | 4.44×10^-16^ |
|  | 5672 | 8740 | JXA1 | NADC30 | 1.35×10^-116^ | 1.61×10^-115^ | 4.90×10-120 | 3.10×10^-41^ | 7.63×10^-43^ | - | 4.44×10^-16^ |
| SCcd17 | 1 | 824 | JXA1 | NADC30 | 1.26×10^-39^ | 4.02×10^-26^ | 8.16×10^-40^ | 1.20×10^-15^ | 5.90×10^-18^ | - | 4.44×10^-16^ |
|  | 5266 | 9675 | JXA1 | NADC30 | 4.59×10^-124^ | 1.40×10^-98^ | 7.02×10^-126^ | 8.68×10^-43^ | 2.24×10^-49^ | - | 4.44×10^-16^ |
|  | 12706 | 12819 | JXA1 | NADC30 | 2.65×10^-4^ | 1.05×10^-2^ | 1.86×10^-4^ | 4.71×10^-3^ | - | - | 2.70×10^-4^ |
|  | 12874 | 13133 | JXA1 | NADC30 | 1.84×10^-12^ | - | 4.83×10^-11^ | 7.54×10^-6^ | 1.66×10^-2^ | - | 2.00×10^-13^ |
| NMGTZJ3157-2310 | 1 | 1160 | JXA1 | NADC30 | 2.20×10^-10^ | 2.03×10^-15^ | 1.28×10^-23^ | 1.36×10^-5^ | 1.45×10^-15^ | - | 2.65×10^-12^ |
|  | 5680 | 9580 | JXA1 | NADC30 | 1.11×10^-6^ | 3.22×10^-26^ | 2.15×10^-21^ | 3.65×10^-18^ | 1.16×10^-10^ | - | 3.33×10^-5^ |
| HLJTZJ3143-2310 | 94 | 1320 | JXA1 | NADC30 | 2.17×10^-20^ | 5.11×10^-6^ | 2.54×10^-20^ | 3.28×10^-11^ | 1.60×10^-3^ | 2.04×10^-11^ | 1.33×10^-15^ |
|  | 1570 | 2168 | JXA1 | NADC30 | 1.78×10^-6^ | - | 1.57×10^-5^ | 3.32×10^-6^ | 1.81×10^-4^ | - | 5.78×10^-7^ |
|  | 5808 | 9618 | JXA1 | NADC30 | 2.73×10^-61^ | 1.73×10^-16^ | 1.70×10^-34^ | 1.36×10^-33^ | 4.85×10^-38^ | 4.76×10^-39^ | 8.88×10^-16^ |
|  | 13627 | 13861 | IA/2014/NADC34 | NADC30 | 1.86×10^-2^ | 1.14×10^-2^ | 1.66×10^-2^ | 6.59×10^-4^ | 1.70×10^-3^ | - | 5.68×10^-3^ |
| CHbj2103 | 53 | 2012 | JXA1 | NADC30 | 6.87×10^-60^ | 9.21×10^-36^ | 1.97×10^-58^ | 1.52×10^-21^ | 1.54×10^-12^ | 8.05×10^-29^ | 1.33×10^-15^ |
|  | 5497 | 5716 | JXA1 | NADC30 | 8.62×10^-12^ | 4.43×10^-7^ | 6.21×10^-12^ | 4.39×10^-4^ | 1.03×10^-2^ | - | 1.99×10^-8^ |
|  | 8366 | 8882 | JXA1 | NADC30 | 7.45×10^-25^ | 7.52×10^-12^ | 5.94×10^-23^ | 5.13×10^-12^ | 1.33×10^-13^ | 7.82×10^-12^ | 1.78×10^-15^ |
|  | 11963 | 13530 | QYYZ | NADC30 | 1.52×10^-69^ | 1.54×10^-51^ | 1.36×10^-65^ | 5.71×10^-22^ | 3.22×10^-2^ | 7.03×10^-31^ | 1.78×10^-15^ |
| CHbj2102 | 534 | 1082 | JXA1 | NADC30 | 1.36×10^-25^ | 3.53×10^-15^ | 3.17×10^-25^ | 1.49×10^-4^ | 3.87×10^-5^ | 2.17×10^-14^ | 2.60×10^-7^ |
|  | 1312 | 1812 | JXA1 | NADC30 | 1.19×10^-16^ | - | 3.71×10^-18^ | 8.64×10^-11^ | 4.89×10^-3^ | 2.66×10^-8^ | 1.33×10^-15^ |
|  | 5686 | 8882 | JXA1 | NADC30 | 1.32×10^-30^ | 8.40×10^-22^ | 3.85×10^-31^ | 7.05×10^-17^ | 8.50×10^-20^ | 2.86×10^-20^ | 1.19×10^-8^ |
|  | 11984 | 13529 | QYYZ | NADC30 | 7.00×10^-52^ | 1.37×10^-38^ | 1.18×10^-50^ | 5.93×10^-21^ | 6.99×10^-26^ | 4.16×10^-28^ | 1.33×10^-15^ |
| CHbj2101 | 540 | 1102 | JXA1 | NADC30 | - | 3.15×10^-15^ | 2.80×10^-25^ | 5.70×10^-14^ | 1.52×10^-3^ | 1.68×10^-14^ | 5.54×10^-7^ |
|  | 1312 | 1812 | JXA1 | NADC30 | 4.23×10^-15^ | - | 1.71×10^-16^ | 1.10×10-^10^ | 1.50×10^-2^ | 2.66×10^-8^ | 1.33×10^-15^ |
|  | 5686 | 8882 | JXA1 | NADC30 | 4.94×10^-30^ | 1.70×10^-20^ | 1.55×10^-31^ | 1.28×10^-16^ | 4.83×10^-20^ | 1.38×10^-19^ | 1.33×10^-15^ |
|  | 11984 | 13530 | QYYZ | NADC30 | 2.01×10^-60^ | 4.18×10^-44^ | 2.27×10^-57^ | 1.23×10^-25^ | 5.73×10^-3^ | 1.68×10^-30^ | 1.33×10^-15^ |
| SCN17 | 44 | 736 | VR2332 | NADC30 | 9.66×10^-31^ | 2.12×10^-25^ | 2.82×10^-30^ | 1.39×10^-12^ | 1.05×10^-13^ | 3.19×10^-15^ | 1.33×10^-15^ |
|  | 5274 | 6904 | VR2332 | NADC30 | 2.58×10^-84^ | 1.18×10^-84^ | 7.23×10^-89^ | 2.09×10^-27^ | 1.34×10^-27^ | 3.83×10^-36^ | 1.33×10^-15^ |
|  | 7798 | 8071 | VR2332 | NADC30 | 1.43×10^-9^ | 5.85×10^-4^ | 6.85×10^-10^ | 2.01×10^-6^ | 2.64×10^-6^ | 1.44×10^-6^ | 8.73×10^-9^ |
|  | 8431 | 11218 | VR2332 | NADC30 | 1.37×10^-99^ | 6.52×10^-107^ | 1.20×10^-105^ | 1.36×10^-37^ | 1.41×10^-36^ | 2.42×10^-50^ | 1.33×10^-15^ |
|  | 14060 | 14340 | JXA1 | NADC30 | 8.36×10^-22^ | 3.11×10^-14^ | 2.07×10^-22^ | 1.83×10^-10^ | 7.14×10^-11^ | 2.08×10^-4^ | 1.78×10^-15^ |
| SCTZJ3124-2309 | 6097 | 8060 | JXA1 | NADC30 | 2.86×10^-52^ | - | 1.77×10^-51^ | 2.42×10^-21^ | 4.63×10^-24^ | 1.21×10^-22^ | 1.78×10^-15^ |
|  | 10910 | 11498 | JXA1 | NADC30 | 1.69×10^-11^ | 7.15×10^-5^ | 1.70×10^-10^ | 1.57×10^-8^ | 6.95×10^-4^ | - | 2.56×10^-7^ |
|  | 13684 | 14196 | IA/2014/NADC34 | NADC30 | 1.24×10^-17^ | 8.30×10^-10^ | 7.53×10^-18^ | 2.07×10^-5^ | 2.28×10^-4^ | 2.27×10^-10^ | 1.33×10^-15^ |
| HLJTZJ2090-2107 | 1 | 385 | JXA1 | NADC30 | 1.31×10^-4^ | 1.14×10^-2^ | - | 1.72×10^-6^ | 1.46×10^-5^ | - | 2.47×10^-6^ |
|  | 628 | 1082 | JXA1 | NADC30 | 2.47×10^-24^ | 1.15×10^-13^ | 1.46×10^-22^ | 2.99×10^-7^ | 1.75×10^-5^ | 1.18×10^-11^ | 1.78×10^-15^ |
|  | 5606 | 6842 | JXA1 | NADC30 | 3.02×10^-32^ | 3.68×10^-18^ | 7.38×10^-33^ | 1.10×10^-16^ | 3.31×10^-19^ | 3.37×10^-17^ | 1.78×10^-15^ |
|  | 13683 | 14198 | IA/2014/NADC34 | NADC30 | 1.73×10^-17^ | 8.63×10^-10^ | 2.37×10^-18^ | 2.31×10^-8^ | 8.36×10^-10^ | 2.70×10^-11^ | 3.55×10^-15^ |
| HBFL-1604 | 5552 | 6772 | JXA1 | NADC30 | 6.37×10^-63^ | 9.10×10^-36^ | 3.21×10^-63^ | 8.72×10^-20^ | 1.59×10^-22^ | - | 5.55×10^-16^ |
| GX20210501 | 58 | 490 | JXA1 | NADC30 | 7.71×10^-3^ | - | 5.34×10^-3^ | 1.07×10^-6^ | 5.94×10^-6^ | - | 9.47×10^-10^ |
|  | 5554 | 6656 | JXA1 | NADC30 | 9.26×10^-41^ | 5.12×10^-15^ | 3.92×10^-40^ | 6.66×10^-16^ | 2.50×10^-19^ | - | 4.44×10^-16^ |
| GXHX20211106 | 5554 | 6564 | JXA1 | NADC30 | 7.53×10^-42^ | 6.73×10^-16^ | 9.80×10^-44^ | 6.80×10^-17^ | 2.27×10^-10^ | - | 6.99×10^-11^ |
| GXNN202004a | 5554 | 6526 | JXA1 | NADC30 | 5.77×10^-45^ | 2.17×10^-22^ | 8.93×10^-44^ | 9.31×10^-17^ | 2.04×10^-11^ | - | 4.25×10^-11^ |
| JLTZJ2050-2107 | 632 | 1066 | JXA1 | NADC30 | 6.15×10^-26^ | 1.36×10^-17^ | 7.05×10^-27^ | 1.39×10^-10^ | 3.39×10^-8^ | 4.46×10^-12^ | 1.33×10^-15^ |
|  | 1296 | 1574 | IA/2014/NADC34 | NADC30 | 4.70×10^-12^ | 2.45×10^-2^ | 2.72×10^-11^ | 9.15×10^-7^ | 8.73×10^-9^ | - | 4.38×10^-10^ |
|  | 5606 | 6896 | IA/2014/NADC34 | NADC30 | 3.42×10^-3^ | - | - | 3.41×10^-2^ | - | 1.04×10^-4^ | 1.26×10^-2^ |
|  | 7900 | 8860 | JXA1 | NADC30 | 3.62×10^-25^ | - | 1.05×10^-25^ | 1.06×10^-14^ | 9.01×10^-16^ | 2.43×10^-14^ | 8.88×10^-16^ |
|  | 12135 | 14727 | IA/2014/NADC34 | NADC30 | 1.83×10^-60^ | 3.42×10^-39^ | 2.28×10^-38^ | 7.93×10^-27^ | 2.16×10^-28^ | 1.04×10^-24^ | 8.88×10^-16^ |
| HLJTZJ3268-2312 | 496 | 702 | JXA1 | NADC30 | 1.60×10^-5^ | - | 2.03×10^-5^ | 2.01×10^-2^ | 1.82×10^-3^ | - | 3.79×10^-6^ |
|  | 1296 | 2153 | JXA1 | NADC30 | 2.31×10^-46^ | 2.59×10^-24^ | 2.46×10^-46^ | 1.72×10^-19^ | 3.29×10^-23^ | - | 4.44×10^-16^ |
|  | 5371 | 6936 | JXA1 | NADC30 | 1.42×10^-40^ | 1.21×10^-19^ | 1.14×10^-37^ | 2.26×10^-19^ | 1.38×10^-10^ | - | 3.33×10^-16^ |
|  | 7704 | 8043 | JXA1 | NADC30 | 1.84×10^-10^ | 1.21×10^-4^ | 3.07×10^-10^ | 1.51×10^-6^ | 8.56×10^-8^ | - | 4.14×10^-9^ |
| JL580 | 3416 | 4547 | JXA1 | NADC30 | 8.25×10^-79^ | 2.16×10^-55^ | 1.88×10^-79^ | 2.01×10^-26^ | 1.00×10^-28^ | - | 4.44×10^-16^ |
|  | 5188 | 7234 | JXA1 | NADC30 | 2.53×10^-65^ | 3.89×10^-52^ | 1.97×10^-67^ | 8.42×10^-31^ | 1.12×10^-32^ | - | 4.44×10^-16^ |
|  | 12604 | 13547 | JXA1 | NADC30 | 1.91×10^-68^ | 8.85×10^-69^ | 1.81×10^-70^ | 1.38×10^-24^ | 9.86×10^-25^ | - | - |
| CY1-1604 | 3412 | 7233 | JXA1 | NADC30 | 2.04×10^-176^ | 2.64×10^-167^ | 2.87×10^-179^ | 3.27×10^-51^ | 1.98×10^-32^ | - | 4.44×10^-16^ |
| SDbz16-2 | 5248 | 6823 | JXA1 | NADC30 | 4.37×10^-86^ | 1.47×10^-66^ | 6.98×10^-87^ | 9.76×10^-24^ | 2.78×10^-28^ | - | 5.55×10^-16^ |
| SXht2012 | 70 | 1507 | JXA1 | NADC30 | 1.50×10^-39^ | 8.57×10^-13^ | 9.34×10^-57^ | 7.95×10^-22^ | 4.27×10^-25^ | - | 4.44×10^-16^ |
|  | 5268 | 6838 | JXA1 | NADC30 | 1.98×10^-41^ | 7.13×10^-20^ | 1.16×10^-40^ | 1.12×10^-22^ | 2.21×10^-25^ | - | 1.43×10^-9^ |
| GXGG20210301 | 1 | 1501 | JXA1 | NADC30 | - | 4.01×10^-31^ | 1.46×10^-44^ | 2.03×10^-24^ | 1.13×10^-26^ | - | 2.22×10^-16^ |
|  | 5276 | 8024 | JXA1 | NADC30 | 1.60×10^-51^ | 5.76×10^-40^ | 8.80×10^-54^ | 1.28×10^-27^ | 3.63×10^-34^ | - | 4.09×10^-9^ |
|  | 8132 | 8808 | JXA1 | NADC30 | 8.81×10^-30^ | 4.72×10^-26^ | 2.87×10^-28^ | 6.60×10^-13^ | 3.08×10^-15^ | - | - |
|  | 10905 | 11360 | JXA1 | NADC30 | 1.32×10^-10^ | 1.27×10^-5^ | 6.52×10^-11^ | 3.56×10^-6^ | 1.98×10^-8^ | - | - |
| HLJTZJ2007-2106 | 1 | 1514 | JXA1 | NADC30 | 8.33×10^-46^ | 4.59×10^-12^ | 6.41×10^-45^ | 4.47×10^-22^ | 4.48×10^-9^ | 8.57×10^-23^ | 1.33×10^-15^ |
|  | 5308 | 8987 | JXA1 | NADC30 | 2.79×10^-66^ | 1.58×10^-28^ | 5.23×10^-69^ | 1.84×10^-26^ | 4.46×10^-39^ | 1.62×10^-39^ | 8.88×10^-16^ |
|  | 12012 | 13421 | IA/2014/NADC34 | NADC30 | 1.31×10^-51^ | 9.76×10^-45^ | 1.00×10^-52^ | 3.60×10^-24^ | 7.22×10^-16^ | 6.85×10^-30^ | 8.88×10^-16^ |
| HLJWK871-2308 | 55 | 1508 | JXA1 | NADC30 | 4.98×10^-42^ | - | 2.11×10^-38^ | 1.03×10^-20^ | 1.43×10^-9^ | - | 3.33×10^-16^ |
|  | 5257 | 8946 | JXA1 | NADC30 | 1.67×10^-63^ | 1.04×10^-10^ | 5.14×10^-66^ | 3.78×10^-32^ | 4.25×10^-37^ | - | 3.33×10^-16^ |
| LNTZJ3211-2311 | 60 | 1496 | JXA1 | NADC30 | 9.70×10^-44^ | 2.35×10^-12^ | 2.69×10^-42^ | 5.93×10^-21^ | 1.56×10^-22^ | - | 3.33×10^-16^ |
|  | 5250 | 8936 | JXA1 | NADC30 | 4.98×10^-60^ | - | 1.51×10^-60^ | 6.09×10^-33^ | 3.67×10^-14^ | - | 3.33×10^-16^ |
| HN-NY/2023 | 50 | 1380 | JXA1 | NADC30 | 1.81×10^-32^ | 2.25×10^-14^ | 7.66×10^-44^ | 7.13×10^-16^ | 4.47×10^-9^ | - | 2.20×10^-7^ |
|  | 5338 | 8103 | JXA1 | NADC30 | 1.08×10^-57^ | 9.06×10^-17^ | 1.21×10^-54^ | 4.84×10^-27^ | 2.28×10^-31^ | - | 3.33×10^-16^ |
| H×10NZMD-9 | 1 | 1501 | JXA1 | NADC30 | 2.65×10^-89^ | 7.12×10^-73^ | 1.16×10^-87^ | 1.38×10^-28^ | 6.19×10^-30^ | - | 4.44×10^-16^ |
|  | 5266 | 8031 | JXA1 | NADC30 | 6.19×10^-92^ | 1.04×10^-79^ | 4.97×10^-94^ | 1.77×10^-36^ | 5.35×10^-38^ | - | 4.44×10^-16^ |
| PRRSV/H60 | 1 | 2012 | JXA1 | NADC30 | 1.21×10^-73^ | 4.13×10^-61^ | 1.29×10^-74^ | 1.54×10^-30^ | 2.75×10^-35^ | - | - |
|  | 5266 | 8031 | JXA1 | NADC30 | 1.18×10^-97^ | 8.63×10^-56^ | 3.12×10^-98^ | 4.06×10^-29^ | 3.03×10^-35^ | - | 4.44×10^-16^ |
| SC-d | 1 | 1506 | JXA1 | NADC30 | 2.89×10^-83^ | 2.65×10^-63^ | 1.08×10^-83^ | 5.94×10^-28^ | 6.43×10^-30^ | - | 1.72×10^-2^ |
|  | 5272 | 8031 | JXA1 | NADC30 | 2.22×10^-85^ | 1.62×10^-58^ | 2.00×10^-86^ | 1.07×10^-33^ | 3.84×10^-37^ | - | 4.44×10^-16^ |
| HuBXW | 1 | 1520 | JXA1 | NADC30 | 4.07×10^-65^ | 2.50×10^-40^ | 2.20×10^-66^ | 4.45×10^-24^ | 5.21×10^-6^ | - | 6.14×10^-12^ |
|  | 5266 | 8031 | JXA1 | NADC30 | 7.55×10^-63^ | - | 1.13×10^-63^ | 2.26×10^-29^ | 1.42×10^-31^ | - | 3.33×10^-16^ |
| PRRSV/S043 | 1 | 1501 | JXA1 | NADC30 | 8.61×10^-71^ | 8.42×10^-44^ | 2.83×10^-67^ | 4.56×10^-25^ | 3.92×10^-5^ | - | 4.44×10^-16^ |
|  | 5266 | 8031 | JXA1 | NADC30 | 2.66×10^-76^ | 4.25×10^-43^ | 8.05×10^-78^ | 3.98×10^-31^ | 3.05×10^-34^ | - | 3.33×10^-16^ |
| PRRSV/H029 | 7834 | 8174 | JXA1 | NADC30 | 2.75×10^-22^ | 8.47×10^-14^ | 1.74×10^-19^ | 4.82×10^-9^ | 5.69×10^-6^ | - | 1.13×10^-3^ |
| HuN-XT-B | 1 | 1049 | JXA1 | NADC30 | 3.48×10^-36^ | - | 4.89×10^-35^ | 3.00×10^-21^ | 6.12×10^-20^ | - | 5.55×10^-16^ |
|  | 8067 | 8749 | JXA1 | NADC30 | 2.29×10^-38^ | 1.01×10^-32^ | 3.55×10^-36^ | 6.23×10^-15^ | 9.80×10^-16^ | - | - |
| GD1909 | 1 | 1281 | JXA1 | NADC30 | 4.62×10^-75^ | 6.42×10^-67^ | 2.24×10^-76^ | 2.78×10^-21^ | 1.72×10^-25^ | - | 4.44×10^-16^ |
|  | 4280 | 5845 | JXA1 | NADC30 | 3.27×10^-101^ | 4.60×10^-104^ | 1.15×10^-104^ | 1.04×10^-31^ | 5.10×10^-32^ | - | 4.44×10^-16^ |
|  | 7740 | 8020 | JXA1 | NADC30 | 7.40×10^-18^ | 3.22×10^-13^ | 9.09×10^-17^ | 2.71×10^-9^ | 4.05×10^-4^ | - | 2.78×10^-15^ |
| HNTZJ3182-2310 | 663 | 1273 | JXA1 | NADC30 | 5.45×10^-53^ | 3.94×10^-45^ | 1.61×10^-53^ | 6.52×10^-20^ | 2.01×10^-15^ | - | 5.80×10^-11^ |
|  | 12934 | 13082 | JXA1 | NADC30 | 2.33×10^-9^ | 3.49×10^-5^ | 4.40×10^-10^ | 2.04×10^-5^ | 1.11×10^-4^ | - | 1.69×10^-8^ |
| PRRSV2/CN/F5/2018 | 700 | 1711 | JXA1 | NADC30 | 1.90×10^-57^ | 9.18×10^-34^ | 2.03×10^-58^ | 2.99×10^-23^ | 1.43×10^-8^ | - | 5.55×10^-16^ |
| PRRSV2/CN/G7/2018 | 1 | 2012 | JXA1 | NADC30 | 7.95×10^-78^ | 5.35×10^-50^ | 8.23×10^-80^ | 3.60×10^-30^ | 1.01×10^-32^ | - | 1.55×10^-6^ |
|  | 12604 | 12742 | JXA1 | NADC30 | 2.98×10^-5^ | 3.11×10-3 | 1.65×10^-5^ | 9.15×10^-3^ | 2.19×10^-3^ | - | - |
| FJDJQ-2017 | 12171 | 13084 | QYYZ | NADC30 | 5.65×10-27 | - | 4.25×10^-26^ | 1.57×10^-9^ | 1.29×10^-11^ | 3.84×10^-9^ | 2.22×10^-15^ |
|  | 13778 | 15128 | QYYZ | NADC30 | 5.07×10^-27^ | - | 1.40×10^-23^ | 9.59×10^-16^ | 1.04×10^-16^ | 1.08×10^-10^ | 2.66×10^-15^ |
| PRRSV2/CN/G8/2018 | 694 | 2012 | JXA1 | NADC30 | 9.96×10^-90^ | 9.78×10^-82^ | 6.17×10^-91^ | 2.89×10^-32^ | 9.09×10^-21^ | 4.65×10^-35^ | 2.53×10^-2^ |
|  | 12170 | 12931 | QYYZ | NADC30 | 2.10×10^-25^ | 4.56×10^-10^ | 5.19×10^-25^ | 1.83×10^-13^ | 1.73×10^-15^ | 1.19×10^-15^ | 2.43×10^-3^ |
|  | 12982 | 13610 | JXA1 | NADC30 | 5.05×10^-16^ | - | 1.15×10^-19^ | 1.38×10^-9^ | 3.05×10^-4^ | 9.64×10^-4^ | 1.78×10^-15^ |
|  | 13775 | 15097 | QYYZ | NADC30 | 6.23×10^-28^ | - | 5.79×10^-25^ | 3.08×10^-16^ | 1.07×10^-16^ | 9.87×10^-10^ | 1.74×10^-3^ |
| PRRSV2/CN/G9/2018 | 694 | 2012 | JXA1 | NADC30 | 2.21×10^-93^ | 2.75×10^-84^ | 9.25×10^-95^ | 3.62×10^-32^ | 1.22×10^-32^ | 1.32×10^-36^ | 1.78×10^-15^ |
|  | 12170 | 13608 | QYYZ | NADC30 | 5.40×10^-28^ | - | 7.55×10^-27^ | 7.52×10^-17^ | 1.39×10^-18^ | 3.34×10^-13^ | 2.22×10^-15^ |
|  | 13938 | 15126 | QYYZ | NADC30 | 2.06×10^-29^ | - | 8.06×10^-23^ | 7.19×10^-16^ | 3.18×10^-8^ | 2.87×10^-8^ | 1.46×10^-3^ |
| Fujian-2014-18 | 758 | 1662 | JXA1 | NADC30 | 4.18×10^-43^ | 3.15×10^-13^ | 1.75×10^-42^ | 6.49×10^-20^ | 1.42×10^-7^ | 1.35×10^-15^ | 4.88×10^-15^ |
|  | 15432 | 605 | JXA1 | NADC30 | 2.10×10^-11^ | - | 3.36×10^-12^ | 1.03×10^-10^ | 4.94×10^-5^ | 1.87×10^-4^ | 2.22×10^-15^ |
| HNLCL15-1903 | 1 | 1662 | JXA1 | NADC30 | 2.59×10^-33^ | - | 2.38×10^-33^ | 7.38×10^-13^ | 2.21×10^-24^ | 4.78×10^-19^ | 2.49×10^-14^ |
|  | 11896 | 12880 | QYYZ | NADC30 | 8.99×10^-22^ | - | 1.72×10^-21^ | 1.20×10^-8^ | 1.78×10^-9^ | 1.67×10^-15^ | 7.37×10^-4^ |
|  | 13000 | 14766 | QYYZ | NADC30 | 2.57×10^-18^ | - | 1.53×10^-20^ | 1.07×10^-4^ | 9.47×10^-3^ | 3.43×10^-8^ | 1.78×10^-15^ |
| CH/SCNC-2/2020 | 1 | 1662 | JXA1 | NADC30 | 2.18×10^-31^ | - | 5.22×10^-23^ | 1.25×10^-32^ | 8.64×10^-7^ | - | 1.11×10^-16^ |
|  | 10908 | 11428 | JXA1 | NADC30 | 1.37×10^-4^ | - | 3.27×10^-4^ | 1.97×10^-6^ | 3.08×10^-4^ | - | 9.24×10^-6^ |
|  | 12281 | 13392 | JXA1 | NADC30 | 1.49×10^-14^ | 1.58×10^-7^ | 3.48×10^-16^ | 5.60×10^-10^ | 3.47×10^-7^ | - | 2.00×10^-14^ |
| SCya18 | 1 | 1662 | JXA1 | NADC30 | 9.71×10^-33^ | - | 3.73×10^-32^ | 1.35×10^-24^ | 6.84×10^-13^ | 1.72×10^-19^ | 1.78×10^-15^ |
|  | 11896 | 12880 | QYYZ | NADC30 | 7.03×10^-22^ | - | 8.67×10^-22^ | 2.06×10^-9^ | 5.39×10^-10^ | 2.12×10^-15^ | 5.25×10^-3^ |
| CHsx1401 | 10982 | 11562 | VR2332 | NADC30 | 6.59×10^-27^ | 4.05×10^-16^ | 1.43×10^-27^ | 1.07×10^-10^ | 5.62×10^-12^ | - | 5.55×10^-16^ |
| PRRSV/LN86 | 11012 | 11548 | VR2332 | NADC30 | 2.18×10^-24^ | 2.67×10^-13^ | 7.04×10^-25^ | 1.24×10^-11^ | 5.89×10^-4^ | - | 4.44×10^-16^ |
| LNCH-1604 | 13282 | 13386 | IA/2014/NADC34 | NADC30 | 1.32×10^-5^ | 4.71×10^-4^ | 8.20×10^-6^ | 1.36×10^-2^ | 4.62×10^-2^ | - | 3.46×10^-5^ |
| CH/SCYB-2/2019 | 7797 | 8097 | VR2332 | NADC30 | 4.95×10^-8^ | 6.77×10^-4^ | 2.55×10^-8^ | 9.47×10^-4^ | 3.53×10^-4^ | - | 3.94×10^-7^ |
| BL2019 | 5661 | 6336 | JXA1 | NADC30 | 4.89×10^-54^ | 8.62×10^-56^ | 1.73×10^-56^ | 4.50×10^-19^ | 1.07×10^-13^ | - | 5.55×10^-16^ |
|  | 7847 | 7933 | JXA1 | NADC30 | 1.40×10^-2^ | - | 2.31×10^-2^ | - | 2.78×10^-2^ | - | 2.69×10^-2^ |
| CH/SCCD-2/2018 | 5680 | 6351 | JXA1 | NADC30 | 1.82×10^-59^ | 2.15×10^-63^ | 2.55×10^-62^ | 2.96×10^-19^ | 1.34×10^-14^ | - | 5.55×10^-16^ |
|  | 7791 | 7932 | JXA1 | NADC30 | 6.79×10^-3^ | - | 1.66×10^-2^ | 3.43×10^-2^ | 1.47×10^-2^ | - | 2.36×10^-2^ |
| H×10NAN-XINX | 4608 | 6420 | VR2332 | NADC30 | 8.61×10-^137^ | 2.13×10^-143^ | 5.07×10^-143^ | 5.51×10^-35^ | 5.71×10^-35^ | - | 4.44×10^-16^ |
| HNJYH-1606 | 10839 | 12020 | JXA1 | NADC30 | 1.86×10^-66^ | 3.77×10^-63^ | 7.01×10^-68^ | 5.18×10^-20^ | 2.85×10^-19^ | - | 5.55×10^-16^ |
| HNjz15 | 13031 | 13450 | IA/2014/NADC34 | NADC30 | 6.49×10^-9^ | 3.21×10^-3^ | - | 1.15×10^-3^ | 4.66×10^-2^ | - | 1.01×10^-3^ |
| HLJTZJ3187-2310 | 11876 | 12954 | IA/2014/NADC34 | NADC30 | 1.74×10^-47^ | 5.75×10^-36^ | 6.98×10^-50^ | 7.77×10^-22^ | 3.51×10^-14^ | - | 2.18×10^-14^ |
|  | 13392 | 14198 | IA/2014/NADC34 | NADC30 | 4.76×10^-27^ | 3.70×10^-13^ | 1.79×10^-28^ | 8.54×10^-7^ | 3.00×10^-9^ | - | 1.40×10^-5^ |
| H×10B-108 | 11888 | 13714 | JXA1 | NADC30 | 3.82×10^-95^ | 3.60×10^-112^ | 1.04×10^-100^ | 1.74×10^-31^ | 1.77×10^-31^ | - | 5.55×10^-16^ |
| H×10NJY-2 | 1246 | 2136 | JXA1 | NADC30 | 2.88×10^-40^ | 1.47×10^-10^ | 1.34×10^-36^ | 7.86×10^-20^ | 3.09×10^-12^ | - | 5.55×10^-16^ |
|  | 11878 | 13089 | JXA1 | NADC30 | 9.30×10^-72^ | 3.68×10^-72^ | 2.65×10^-73^ | 1.51×10^-18^ | 2.30×10^-23^ | - | 4.44×10^-16^ |
|  | 13414 | 13714 | JXA1 | NADC30 | 1.07×10^-4^ | - | 6.72×10^-4^ | 7.61×10^-5^ | 4.64×10^-4^ | - | 1.84×10^-5^ |
| HNJYF-1606 | 7630 | 7984 | JXA1 | NADC30 | 1.77×10^-11^ | 2.95×10^-8^ | 8.99×10^-12^ | 6.49×10^-8^ | 2.23×10^-8^ | - | - |
|  | 12060 | 12510 | JXA1 | NADC30 | 5.92×10^-8^ | - | 8.30×10^-7^ | 7.55×10^-4^ | 4.89×10^-4^ | - | 2.12×10^-6^ |
|  | 12580 | 12746 | JXA1 | NADC30 | 4.27×10^-7^ | 2.49×10^-3^ | 2.11×10^-13^ | - | 5.86×10^-3^ | - | 3.64×10^-14^ |
| WUH6 | 11618 | 11974 | JXA1 | NADC30 | 5.45×10^-20^ | 4.94×10^-19^ | 1.36×10^-19^ | 1.04×10^-6^ | 7.65×10^-9^ | - | 6.66×10^-16^ |
| GDHZ | 11148 | 11534 | JXA1 | NADC30 | 1.66×10^-8^ | - | 8.95×10^-9^ | 2.07×10^-4^ | 9.83×10^-6^ | - | 6.73×10^-9^ |
| PRRSV2/CN/I9/2018 | 7710 | 8191 | JXA1 | NADC30 | 7.06×10^-10^ | - | 1.83×10^-8^ | 1.15×10^-8^ | 8.85×10^-6^ | 4.02×10^-2^ | 1.78×10^-11^ |
|  | 11586 | 12019 | JXA1 | NADC30 | 1.16×10^-13^ | 2.11×10^-4^ | 1.49×10^-14^ | 4.04×10^-6^ | 3.25×10^-7^ | 2.95×10^-5^ | 2.22×10^-15^ |
|  | 12054 | 13472 | QYYZ | NADC30 | 1.99×10^-38^ | 1.17×10^-14^ | 5.86×10^-38^ | 1.09×10^-19^ | 1.05×10^-7^ | 8.23×10^-15^ | 2.66×10^-15^ |
| qy 2104 | 12170 | 13718 | QYYZ | NADC30 | 1.37×10^-29^ | - | 3.65×10^-25^ | 6.34×10^-16^ | 1.33×10^-17^ | - | 1.10×10^-7^ |
|  | 13477 | 13598 | QYYZ | NADC30 | 1.09×10^-2^ | - | 6.08×10^-3^ | 2.79×10^-2^ | 1.54×10^-2^ | - | - |
| qy 2105 | 12170 | 13718 | QYYZ | NADC30 | 6.91×10^-30^ | - | 5.73×10^-25^ | 5.77×10^-16^ | 1.33×10^-17^ | - | 1.14×10^-7^ |
| GDhy-1809 | 12170 | 13718 | QYYZ | NADC30 | 7.66×10^-30^ | - | 6.28×10^-25^ | 5.77×10^-16^ | 1.33×10^-17^ | - | 1.17×10^-7^ |
|  | 14336 | 15126 | QYYZ | NADC30 | 3.17×10^-3^ | 6.69×10^-3^ | - | 3.18×10^-2^ | 4.19×10^-2^ | - | - |
| PRRSV2/CN/X4839/2017 | 4706 | 4962 | JXA1 | NADC30 | 6.61×10^-35^ | 2.44×10^-33^ | 8.04×10^-30^ | 1.28×10^-12^ | 1.60×10^-12^ | - | 6.66×10^-16^ |
| FJ1402 | 3872 | 5082 | JXA1 | NADC30 | 4.19×10^-110^ | 1.28×10^-109^ | 2.12×10^-112^ | 2.76×10^-28^ | 1.65×10^-28^ | - | 5.55×10^-16^ |
|  | 11433 | 13220 | JXA1 | NADC30 | 1.52×10^-100^ | 4.97×10^-99^ | 5.94×10^-101^ | 3.58×10^-29^ | 5.37×10^-33^ | - | 5.55×10^-16^ |
| FJM4 | 12604 | 13239 | JXA1 | NADC30 | 1.39×10^-43^ | 1.10×10^-25^ | 1.51×10^-44^ | 1.31×10^-14^ | 3.31×10^-16^ | - | 6.66×10^-16^ |
| FJL15 | 4420 | 5648 | JXA1 | NADC30 | 5.02×10^-104^ | 8.86×10^-104^ | 3.25×10^-106^ | 5.37×10^-29^ | 1.77×10^-29^ | - | 5.55×10^-16^ |
|  | 12604 | 13239 | JXA1 | NADC30 | 3.97×10^-34^ | 2.05×10^-15^ | 5.08×10^-34^ | 7.83×10^-15^ | 3.74×10^-15^ | - | 5.55×10^-16^ |
| PRRSV2/CN/N3/2017 | 1 | 1141 | JXA1 | NADC30 | 1.96×10^-7^ | 9.66×10^-4^ | - | 1.66×10^-4^ | 6.47×10^-4^ | - | 6.95×10^-6^ |
|  | 12952 | 13239 | JXA1 | NADC30 | 1.58×10^-19^ | 1.27×10^-14^ | 1.36×10^-20^ | 2.13×10^-6^ | 1.49×10^-6^ | - | 5.55×10^-16^ |
| PRRSV2/CN/X4833/2018 | 1 | 1022 | JXA1 | NADC30 | 6.72×10^-38^ | 1.90×10^-14^ | 1.66×10^-39^ | 5.03×10^-14^ | 3.23×10^-17^ | - | 5.55×10^-16^ |
|  | 12604 | 12896 | JXA1 | NADC30 | 1.07×10^-5^ | 1.09×10^-3^ | 4.27×10^-4^ | 6.60×10^-3^ | 2.09×10^-3^ | - | 9.38×10^-5^ |
|  | 12952 | 13239 | JXA1 | NADC30 | 4.62×10^-18^ | 5.55×10^-10^ | 2.31×10^-18^ | 2.23×10^-4^ | 6.57×10^-5^ | - | 5.55×10^-16^ |
|  | 13410 | 13480 | JXA1 | NADC30 | 1.09×10^-6^ | 1.90×10^-4^ | - | 9.55×10^-3^ | 3.89×10^-2^ | - | - |
| PRRSV2/CN/F7/2017 | 115 | 742 | JXA1 | NADC30 | 5.11×10^-20^ | 5.68×10^-9^ | 2.31×10^-20^ | 1.54×10^-10^ | 2.41×10^-11^ | - | 5.55×10^-16^ |
|  | 960 | 1136 | JXA1 | NADC30 | 8.62×10^-6^ | 7.89×10^-4^ | 5.58×10^-4^ | 1.93×10^-3^ | 2.56×10^-3^ | - | 8.32×10^-4^ |
|  | 12604 | 13239 | JXA1 | NADC30 | 5.67×10^-38^ | 5.38×10^-23^ | 6.12×10^-39^ | 5.39×10^-16^ | 9.87×10^-17^ | - | 5.55×10^-16^ |
|  | 13410 | 13480 | JXA1 | NADC30 | 1.25×10^-4^ | 1.07×10^-4^ | - | 1.41×10^-3^ | 7.57×10^-3^ | - | 6.48×10^-4^ |
| PRRSV2/CN/X9830/2018 | 115 | 734 | JXA1 | NADC30 | 1.84×10^-19^ | 1.33×10^-8^ | 7.31×10^-20^ | 3.05×10^-10^ | 1.04×10^-11^ | - | 5.55×10^-16^ |
|  | 12604 | 13239 | JXA1 | NADC30 | 3.38×10^-36^ | 2.95×10^-22^ | 3.26×10^-37^ | 3.20×10^-16^ | 5.36×10^-17^ | - | 5.55×10^-16^ |
|  | 14060 | 14232 | JXA1 | NADC30 | 2.13×10^-7^ | 5.62×10^-9^ | 2.16×10^-9^ | 4.08×10^-2^ | 6.52×10^-4^ | - | 3.07×10^-6^ |
| HBag-4 | 126 | 538 | JXA1 | NADC30 | 9.96×10^-13^ | 2.20×10^-7^ | 7.08×10^-13^ | 4.65×10^-6^ | 2.23×10^-5^ | - | 3.03×10^-11^ |
|  | 1850 | 2013 | JXA1 | NADC30 | 1.18×10^-22^ | 2.58×10^-16^ | 5.38×10^-21^ | 3.80×10^-10^ | 7.11×10^-11^ | - | 5.55×10^-16^ |
| BJ2021 | 5634 | 6387 | JXA1 | NADC30 | 1.38×10^-24^ | - | 1.87×10^-25^ | 2.80×10^-13^ | 3.57×10^-15^ | - | 5.55×10^-16^ |
|  | 12578 | 12744 | JXA1 | NADC30 | 5.21×10^-6^ | 8.70×10^-4^ | 5.00×10^-6^ | - | - | - | 3.22×10^-8^ |
| JSWA | 7808 | 8071 | VR2332 | NADC30 | 5.68×10^-8^ | 1.69×10^-5^ | 4.08×10^-7^ | 1.15×10^-3^ | 5.10×10^-4^ | - | 4.21×10^-5^ |
| CY2-1604 | 7797 | 8070 | VR2332 | NADC30 | 2.79×10^-10^ | 5.29×10^-5^ | 1.60×10^-10^ | 2.53×10^-7^ | 9.89×10^-7^ | - | 3.26×10^-9^ |
| PRRSV/S145 | 7797 | 8049 | VR2332 | NADC30 | 2.42×10^-7^ | - | 1.34×10^-7^ | 7.91×10^-4^ | 1.63×10^-5^ | - | 4.97×10^-7^ |
| PRRSV/S130 | 7797 | 8049 | VR2332 | NADC30 | 2.42×10^-7^ | - | 1.34×10^-7^ | 7.92×10^-4^ | 1.63×10^-5^ | - | 4.90×10^-7^ |
| HLJTZJ3205-2310 | 5326 | 8087 | JXA1 | NADC30 | 9.16×10^-69^ | 1.77×10^-13^ | 1.49×10^-68^ | 1.38×10^-26^ | 1.43×10^-30^ | 2.05×10^-29^ | 1.78×10^-15^ |
|  | 12138 | 14575 | IA/2014/NADC34 | NADC30 | 2.53×10^-43^ | 3.37×10^-33^ | 4.99×10^-44^ | 1.64×10^-19^ | 5.85×10^-12^ | 1.36×10^-24^ | 1.33×10^-15^ |

-: not significant
