# Supplementary material for: Genomic characteristics and epidemic trends of NADC30-like PRRSV in China
Source: Porcine Health Manag. 2025 May 28;11:30. doi: 10.1186/s40813-025-00444-7 (PMC12121172; doi:10.1186/s40813-025-00444-7)
Supplement: Supplementary file 5 — Supplementary Material 5: Table S4. Comparison of pathogenicity of different NADC30-like strains [file 40813_2025_444_MOESM5_ESM.docx]

| **TABLE S4 Comparison of pathogenicity of different NADC30-like strains** | | | | | | | |
| --- | --- | --- | --- | --- | --- | --- | --- |
| Infected PRRSV strain | Subtype | The days of inoculation (dpi) | Inoculated dose | Parameters of evaluation | Challenge group | Pathogenicity | Reference |
| SD-A19 | UR | 14 | 3×10^5^ TCID_50_ | Clinical symptoms | Mild clinical symptoms | Moderate | [60] |
|  |  |  |  | Days of fever | 4 days(≥40℃) |  |  |
|  |  |  |  | Pathological and histopathological lesions | Mild pathological changes |  |  |
|  |  |  |  | Viremia | - |  |  |
| SCCD22 | UR | 14 | 2×10^5^ TCID_50_ | Clinical symptoms | Mild clinical symptoms | Moderate | [24] |
|  |  |  |  | Days of fever | 8 days(≥40℃) |  |  |
|  |  |  |  | Pathological and histopathological lesions | Mild pathological changes |  |  |
|  |  |  |  | Viremia | Peaked at 5 dpi, longer than 14 days |  |  |
| HB17A | UR | 28 | 3×10^5^ TCID_50_ | Clinical symptoms | Mild clinical symptoms | Moderate | [61] |
|  |  |  |  | Days of fever | 7 days(≥40℃) |  |  |
|  |  |  |  | Pathological and histopathological lesions | Mild pathological changes |  |  |
|  |  |  |  | Viremia | Peaked at 14 dpi, longer than 28 days |  |  |
| SCya18 | IR | 14 | 4×10^5^ TCID_50_ | Clinical symptoms | Mild clinical symptoms | Moderate | [62] |
|  |  |  |  | Days of fever | 9 days(≥40℃) |  |  |
|  |  |  |  | Pathological and histopathological lesions | Mild pathological changes |  |  |
|  |  |  |  | Viremia | Peaked at 7 dpi, longer than 14 days |  |  |
| BL2019 | IR | 14 | 2×10^5^ TCID_50_ | Clinical symptoms | Obvious clinical symptoms, abortion and  weak fetus in gestating sows | Moderate | [63] |
|  |  |  |  | Days of fever | 8days(≥40℃) |  |  |
|  |  |  |  | Pathological and histopathological lesions | Obvious pathological changes |  |  |
|  |  |  |  | Viremia | Peaked at 5 dpi, longer than 14 days |  |  |
| FJ1402 | IR | 14 | 3×10^6^ TCID_50_ | Clinical symptoms | Mild clinical symptoms | Moderate | [12] |
|  |  |  |  | Days of fever | 2 days(≥40℃) |  |  |
|  |  |  |  | Pathological and histopathological lesions | Mild pathological changes |  |  |
|  |  |  |  | Viremia | Peaked at 10 dpi, longer than 14 days |  |  |
| HNjz15 | IR | 14 | 1×10^5^ TCID_50_ | Clinical symptoms | Mild clinical symptoms | Moderate | [11] |
|  |  |  |  | Days of fever | 7 days(≥40℃) |  |  |
|  |  |  |  | Pathological and histopathological lesions | Mild pathological changes |  |  |
|  |  |  |  | Viremia | Peaked at 6 dpi, longer than 14 days |  |  |

| FJZ03 | IR | 14 | 2×10^5^ TCID_50_ | Clinical symptoms | Mild clinical symptoms | Moderate | [64] |
| --- | --- | --- | --- | --- | --- | --- | --- |
|  |  |  |  | Days of fever | 9 days(≥40℃) |  |  |
|  |  |  |  | Pathological and histopathological lesions | Mild pathological changes |  |  |
|  |  |  |  | Viremia | Peaked at 6 dpi, longer than 14 days |  |  |
| SD17-36 | IR | 14 | 2×10^5^ TCID_50_ | Clinical symptoms | Mild clinical symptoms | Low | [65] |
|  |  |  |  | Days of fever | No fever |  |  |
|  |  |  |  | Pathological and histopathological lesions | Mild pathological changes |  |  |
|  |  |  |  | Viremia | - |  |  |
| FJWQ16 | IR | 14 | 2×10^5^ TCID_50_ | Clinical symptoms | Severe clinical symptoms,1/5 pigs died | High | [64] |
|  |  |  |  | Days of fever | 13 days(≥40℃) |  |  |
|  |  |  |  | Pathological and histopathological lesions | Obvious pathological changes |  |  |
|  |  |  |  | Viremia | Peaked at 6 dpi, longer than 14 days |  |  |
| ZJqz21 | IR | 49 | 1×10^5^ TCID_50_ | Clinical symptoms | Mild clinical symptoms | Moderate | [66] |
|  |  |  |  | Days of fever | 7 days(≥40℃) |  |  |
|  |  |  |  | Pathological and histopathological lesions | Mild pathological changes |  |  |
|  |  |  |  | Viremia | Peaked at 37 dpi, longer than 49 days |  |  |
| GXQZ20210403 | IR | 14 | 2×10^4^ TCID_50_ | Clinical symptoms | Severe clinical symptoms,1/5 pigs died | High | [55] |
|  |  |  |  | Days of fever | 6 days(≥40℃) |  |  |
|  |  |  |  | Pathological and histopathological lesions | Obvious pathological changes |  |  |
|  |  |  |  | Viremia | Peaked at 7 dpi, longer than 14 days |  |  |
| YC-2020 | IR | 30 | 2×10^4^ TCID_50_ | Clinical symptoms | Mild clinical symptoms | Low | [67] |
|  |  |  |  | Days of fever | No fever |  |  |
|  |  |  |  | Pathological and histopathological lesions | Obvious pathological changes |  |  |
|  |  |  |  | Viremia | Peaked at 8 dpi, longer than 30 days |  |  |
| SCN17 | IR | 14 | 4×10^5^ TCID_50_ | Clinical symptoms | Mild clinical symptoms | Moderate | [68] |
|  |  |  |  | Days of fever | 12 days(≥40℃) |  |  |
|  |  |  |  | Pathological and histopathological lesions | Mild pathological changes |  |  |
|  |  |  |  | Viremia | Peaked at 7 dpi, longer than 14 days |  |  |
| GXFCG20210401 | IR | 14 | 2×10^4^ TCID_50_ | Clinical symptoms | Mild clinical symptoms | Moderate | [55] |
|  |  |  |  | Days of fever | 5 days(≥40℃) |  |  |
|  |  |  |  | Pathological and histopathological lesions | Mild pathological changes |  |  |
|  |  |  |  | Viremia | - |  |  |
| SC-d | IR | 14 | 3×10^5^ TCID_50_ | Clinical symptoms | Mild clinical symptoms | Moderate | [60] |
|  |  |  |  | Days of fever | 10 days(≥40℃) |  |  |
|  |  |  |  | Pathological and histopathological lesions | Mild pathological changes |  |  |
|  |  |  |  | Viremia | - |  |  |
| JL580 | IR | 14 | 3×10^4^ TCID_50_ | Clinical symptoms | Severe pathological changes  5/5 pigs died | High | [20] |
|  |  |  |  | Days of fever | 3-13 days(≥40℃) |  |  |
|  |  |  |  | Pathological and histopathological lesions | Obvious pathological changes |  |  |
|  |  |  |  | Viremia | - |  |  |
